# Nicotine flux as a powerful tool for regulating nicotine delivery from e-cigarettes: Protocol of two complimentary randomized crossover clinical trials

**DOI:** 10.1371/journal.pone.0291786

**Published:** 2023-09-21

**Authors:** Ahmad El-Hellani, Elyana Hanna, Mehak Sharma, Reagan Blohowiak, Phillip Joseph, Tore Eid, Haleh Nadim, Rachel El-Hage, Rola Salman, Nareg Karaoghlanian, Ayomipo Adeniji, Sally Salam, Farid Talih, Martine Elbejjani, Alison Breland, Thomas Eissenberg, Alan Shihadeh, Stephen R. Baldassarri, Soha Talih

**Affiliations:** 1 Division of Environmental Health Sciences, College of Public Health, The Ohio State University, Columbus, Ohio, United States of America; 2 Center for Tobacco Research, The Ohio State University Comprehensive Cancer Center, Columbus, Ohio, United States of America; 3 Department of Mechanical Engineering, Maroun Semaan Faculty of Engineering and Architecture, American University of Beirut, Beirut, Lebanon; 4 Center for the Study of Tobacco Products, Virginia Commonwealth University, Richmond, Virginia, United States of America; 5 Section of Pulmonary, Critical Care, and Sleep Medicine, Yale School of Medicine, New Haven, Connecticut, United States of America; 6 Department of Laboratory Medicine, Yale School of Medicine, Washington, DC, United States of America; 7 Department of Chemistry, Faculty of Arts and Sciences, American University of Beirut, Beirut, Lebanon; 8 Clinical Psychiatry, Faculty of Medicine, American University of Beirut, Beirut, Lebanon; 9 Clinical Research Institute & Department of Internal Medicine, Faculty of Medicine, American University of Beirut, Beirut, Lebanon; 10 Department of Psychology, Virginia Commonwealth University, Richmond, Virginia, United States of America; 11 Yale Center for the Study of Tobacco Product Use and Addiction, Yale University, New Haven, Connecticut, United States of America; 12 Program in Addiction Medicine, Yale School of Medicine, Washington, DC, United States of America; UNITED KINGDOM

## Abstract

**Introduction:**

Electronic cigarette (EC) use has increased rapidly in the last decade, especially among youth. Regulating nicotine delivery from ECs could help curb youth uptake and leverage EC use in harm reduction yet is complicated by varying device and liquid variables that affect nicotine delivery. Nicotine flux, the nicotine emission rate, is a parameter that incorporates these variables and focuses on the performance rather than the design of an EC. Nicotine flux therefore could be a powerful regulatory tool if it is shown empirically to predict nicotine delivery and subjective effects related to dependence.

**Methods and analysis:**

This project consists of two complementary clinical trials. In Trial I, we will examine the relationship between nicotine flux and the rate and dose of nicotine delivery from ECs, hence, impacting abuse liability. It will also examine the extent to which this relationship is mediated by nicotine form (i.e., freebase versus protonated). At Yale School of Medicine (YSM), study participants will puff EC devices under conditions that differ by flux and form, while arterial blood is sampled in high time resolution. In Trial II, we will assess the relationship between nicotine flux, form, and subjective effects. At the American University of Beirut (AUB), participants will use EC devices with varying nicotine fluxes and forms, while dependency measures, such as the urge to use ECs, nicotine craving, and withdrawal symptoms, will be assessed. We will also monitor puffing intensity and real-time exposure to toxicants.

**Ethics and dissemination:**

The protocol of Trial I and Trial II was approved by YSM and AUB IRBs, respectively. We will disseminate study results through peer-reviewed publications and conference presentations.

**Trial registration:**

NCT05706701 for Trial I and NCT05430334 for Trial II.

## Introduction

### Background and rationale

The prevalence of electronic cigarette (EC) use worldwide is growing rapidly, especially among youth and young adults, some of whom have never used any other nicotine-delivering product [1–3]. For example, in 2022 in the United States, 14.1% of high school students and 3.7% of middle school students reported current use of ECs, with EC being the most commonly used tobacco product in this group [4]. Indeed, the appeal and prevalence of EC among youth are usually attributed to several factors including flavoring, customizability, and effective nicotine delivery [5–10]. While there is strong reason to believe that flavors contribute to EC appeal in youth, [11–13] sustained use could be attributed to efficient nicotine delivery [14]. Early EC devices (i.e., cig-a-like) were flavored yet delivered little or no nicotine to the user and their use was not sustained [15]. However, later product development led to efficient nicotine delivery from ECs that could exceed that of combustible cigarettes [14, 16]. Another innovation was using nicotine salts in EC liquids. Nicotine salts are less harsh than freebase nicotine, and make it easier for tobacco-naive users to inhale aerosols from high concentration liquids (5–6%) [17, 18]. The nearly unlimited combinations of operating variables (i.e., electrical power, liquid composition) generally increased the rate at which nicotine is emitted (i.e., “nicotine flux”; Shihadeh & Eissenberg, 2015) across EC products and increased abuse liability of this product class [19–22].

To mitigate risk, regulators considered capping EC liquid nicotine concentration. Such a policy became law in the European Union (EU) in 2014 and in Canada in 2021, with a limit of 20 mg/mL nicotine on EC liquids [23, 24]. In the USA, the states of Massachusetts and Utah have limited nicotine concentrations to 35 and 36 mg/mL, respectively. Both states’ approach was recently shown to yield a reduction in average nicotine strength purchased, yet with no impact on unit sales compared to states without nicotine concentration limits [25]. However, a major drawback of this approach is that users in a jurisdiction where nicotine concentration is limited may circumvent the intent of the regulation to control nicotine delivery from ECs by opting for higher-powered devices (i.e., open-system ECs) [26], and in the process increase their exposure to respiratory toxicants [27]. A more promising regulatory approach focuses on regulating nicotine flux, the rate at which an EC emits nicotine (e.g. micrograms per second) [28]. As previously demonstrated, nicotine flux is a performance parameter that accounts for the overlapping effects of liquid composition (including nicotine concentration), and device power [29].

Another factor that can impact EC nicotine delivery is the form of nicotine employed. Nicotine in tobacco products can exist in freebase and salt (protonated nicotine) forms. While early ECs employed freebase nicotine, the industry switched to protonated nicotine liquids that are far less harsh to inhale, making these products more appealing to novice users [30]. Products containing nicotine salts expose users to higher nicotine flux [17, 31]. Apart from sensory effects, the speed and dose of nicotine delivered to the brain and the subjective effects related to this delivery are likely mediated by nicotine form [32, 33].

This article summarizes the protocol of a project that aims to validate nicotine flux as a regulatory tool for nicotine delivery from ECs. To do so, this project should show empirically that nicotine flux correlates with nicotine delivery parameters and subjective effects related to nicotine dependence. Nicotine dependence, as with other drugs, is a function of the dose delivered, the speed of delivery, and subjective drug effects [34]. Smoking a combustible cigarette is an efficient and rapid nicotine delivery method [35]. A recent brain imaging study showed that under some conditions, EC delivered nicotine to the brain at a similar rate as a combustible cigarette [36]. Also, several studies have demonstrated that EC nicotine delivery to the user’s body depends on the combination of several operating variables [14]. Thus, nicotine flux, a parameter that encapsulates all operating variables of EC could also capture drug dependence variables such as nicotine delivery rate and dose, and this explanatory power makes it a potent regulatory tool. Moreover, flux could be predicted easily using a validated physics-based mathematical model [37].

### Objective and design

The project consists of two complementary randomized crossover clinical trials that assess the correlation between nicotine flux and form with nicotine delivery and EC abuse liability. In Trial I, we will examine the relationship between nicotine flux, nicotine form, and the rate and dose of nicotine delivery. In the clinical lab at Yale School of Medicine (YSM), participants will puff EC devices under conditions that differ by flux and form, while arterial blood is sampled in high-time resolution. The outcome will indicate the degree to which nicotine flux and form determine the speed and dose of EC nicotine delivery, and thus, contribute to abuse liability. In Trial II, we will assess the relationship between nicotine flux, nicotine form, and subjective effects. At the American University of Beirut (AUB), participants will use EC devices with varying nicotine fluxes and forms. Subjective effects, such as the urge to smoke, craving, and nicotine abstinence symptoms, will be assessed. Outcomes will indicate the degree to which nicotine flux and form influence subjective effects, puffing intensity, and exposure to toxicants. The latter will be assessed using real-time sampling during study sessions.

### Design considerations

#### Two complimentary trials

This project is based on designs, procedures, and outcome measures that have been used successfully in numerous peer-reviewed publications and extensive preliminary work on the two study sites [38–41].

#### Selection of flux levels

S1 Fig shows the extent to which ECs vary in terms of nicotine flux, and thus potentially abuse liability [42, 43], from the equivalent of a nicotine patch to double that of a combustible cigarette [44–49]. The nicotine fluxes selected for this project (8–35 mg/mL) are representative of ECs available in the global market, specifically the highly prevalent pod-based ECs [7, 50].

## Methods and analysis

The protocols of both clinical trials were prepared in accordance with the 33-item checklist of the Standard Protocol Items Recommendations for Interventional Trials (SPIRIT) [51].

### Patient and public involvement

Patients or the public were not involved in the design, conduct, reporting, or data dissemination.

### Participants

For both trials, all participants must be healthy and above 21 years of age (18 for Trial II as this is the legal minimum age for tobacco use in Lebanon). In addition, participants must be willing to provide informed consent, attend lab visits, and abstain from tobacco/nicotine as required. History of active cardiovascular disease, low/high blood pressure, seizures, regular use of prescription medication (except vitamins/birth control), and last month’s use of cocaine, opioids, benzodiazepines, or methamphetamines. Individuals who report using marijuana for >15 days in the last 30 days will be excluded. Women will be excluded if they are breastfeeding or test positive for pregnancy (by urine test) at screening. Participants intending to quit tobacco/nicotine use in the next 30 days will be excluded and referred to cessation treatment. Prior to each session, participants will be instructed to abstain from nicotine/tobacco and/or EC use and all participants will undergo an hour-long observation period prior to each study session during which no nicotine/tobacco product will be permitted [52]. We are recruiting current EC users at both sites defined as ≥ 3 days/week of EC use for at least the past 3 months.

### Study products

We are using the Subox Mini C EC device as this device has been proven to deliver nicotine efficiently to the blood in our previous work [53]. All necessary Subox Mini C devices and accessories were purchased from the US market. EC liquids will be prepared from a stock of analytical grade 30/70 PG/GL liquid. This ratio is prevalent in EC devices, including JUUL [43]. We predict with our mathematical model that for a Subox Mini C operating at 20 W with a 30/70 PG/VG liquid, nicotine concentrations of 2, 4, 7, and 10 mg/mL will provide fluxes of 8, 18, 27, and 35 μg/sec, respectively. Protonated nicotine EC liquids will be prepared by adding a stoichiometric ratio of benzoic acid to the stock solution of a given concentration. The predicted nicotine fluxes for the Subox Mini C will be empirically verified at AUB using smoking machine yields divided by total puff duration. For Trial I, EC liquids will be prepared at The Ohio State University in Dr. El-Hellani’s lab, while for Trial II, EC liquids were prepared in the AUB analytical lab. All EC liquids are stored in the dark at 5°C in sealed containers.

### Trial I methods

#### Recruitment and retention

**Fig 1:**
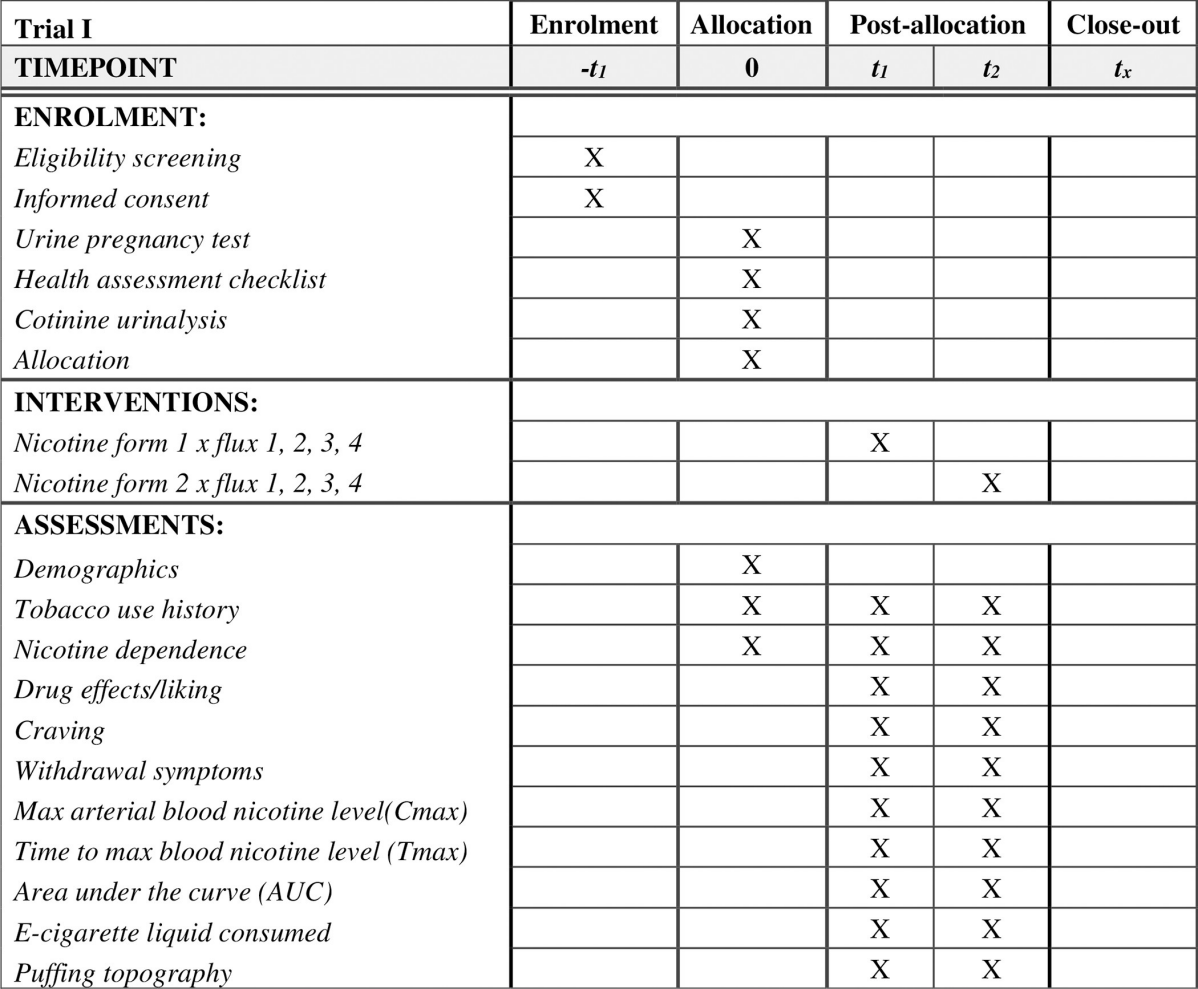
Standard protocol items: Recommendations for interventional trials (SPIRIT) recommended content for the schedule of enrolment, interventions, and assessments for Trial I.

For Trial I (currently recruiting), we estimated that we need 15 participants to detect the within-subject effect of nicotine flux*form on nicotine delivery measured in arterial blood. We restricted randomization to fluxes per each form (i.e., 100% freebase or 100% nicotine salt) (Fig 1). To maximize retention, each participant will be paid $800 for completing a total of two visits (see below). This payment is for the time and inconvenience associated with participation and the potential discomfort caused by arterial blood sampling. Recruitment started at 02/01/2023 and is expected to end by 01/31/2025.

#### Trial design

This study involves a 4 x 2 crossover experimental design of four nicotine fluxes: 8, 18, 27, and 35 μg/sec and two nicotine forms (i.e., freebase and protonated). The selected nicotine fluxes were chosen to represent potential normative regulatory standards.

The YSM has a human research unit equipped with the necessary clinical setup and equipment to withdraw arterial blood. It also has a research laboratory (directed by Dr. Tore Eid) to measure nicotine and nicotine metabolites in biological fluids using liquid chromatography-tandem mass spectrometry (LC-MS/MS, Waters Xevo TQ-XS, Waters Corporation, Milford, MA) [41]. Participants are being asked to puff on the Subox Mini EC device at designated fluxes and arterial blood samples will be collected at baseline and different time points during the vaping session. The participant will vape all four fluxes of one nicotine form in a randomized order for a total of 4 vaping sessions per visit. The participant’s puffing time and inter-puff interval will be controlled via an AUB-developed LabVape. The LabVape device is an ARL research instrument that regulates the electrical power to the EC during use, and which can be programmed to limit puff duration, minimum inter puff interval, and number of puffs drawn by a user. In addition, the participant’s puffing behavior will be monitored and verified using eTop, a technology developed by the AUB team to record puffing parameters.

#### Detailed study procedures

**Fig 2:**
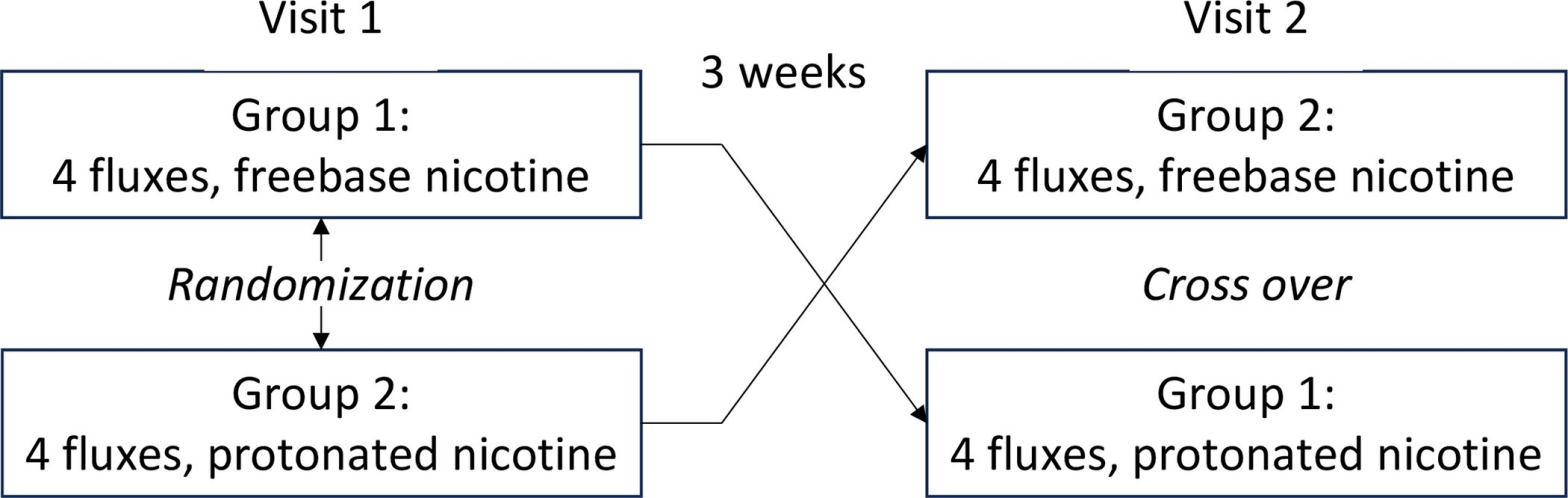
Trial I study design and restricted randomization of conditions.

The flux/form conditions will be tested by participants in two lab visits separated by two to three weeks to minimize carryover effects (Fig 2).

All sessions will be double-blind. Participants will use the EC device on the first visit with one nicotine form and four fluxes in random order. Participants will be instructed to attend the lab for a second visit, to test the four fluxes but with the other nicotine form. The second visit will allow us to isolate the effect of nicotine form on nicotine pharmacokinetics [54]. The order of nicotine form in the two visits will be counterbalanced across participants.

Participants will be instructed to use the Subox Mini C EC in four bouts separated by a 60 min washout period. This period was determined based on our previous arterial measurements on EC; it is deemed sufficient for the blood nicotine to return to baseline [41]. All EC use bouts will be directed; each bout will consist of 3 puffs in which puff duration is fixed to 3 sec and the inter-puff interval is fixed to 30 sec (CORESTA recommended method No 81) [55]. The puff duration and inter-puff interval will be fixed using LabVape. The eTop hardware/software will record the puffing topography to identify any deviation between directed and actual puffs drawn, for data analysis purposes. Participants will be trained to follow the puffing cues prior to sampling using an unpowered EC device.

For arterial blood sampling, a radial arterial line will be placed on the non-dominant side to provide access to blood samples during vaping sessions. Each blood sample (1.0–2.0cc) will be drawn manually by a trained nurse at every time point, transferred to a collecting tube (BD Vacutainer Serum Tube with Plus Serum/CAT with Clot Activator, Becton, Dickinson and Company, Franklin Lakes, NJ) and allowed to coagulate at room temperature for 30 to 60 minutes. The tube will be centrifuged and the serum will be transferred to a clean, polypropylene tube and stored in the freezer at -80°C until analysis. After thawing, nicotine, cotinine, and 3-OH cotinine will be measured in the serum using LC-MS/MS with deuterated internal standards [56]. Blood will be sampled 30 sec prior to the initial puff, 5, 15, and 25 sec after each puff, and 60 sec after the last puff of a bout. The obtained data will be used to calculate the pharmacokinetic parameters of nicotine delivery under each condition.

### Trial I measures

Measures of interest include demographic data about age, gender, race, socio-economic status, marital status, educational, and occupational levels will be assessed using a screening survey. Also, the collected measures include maximum arterial blood level nicotine concentration (Cmax), the rate of nicotine rise after the initial puff (dCi/dt), time to maximum arterial blood level nicotine concentration (Tmax), the area under the curve (AUC) for nicotine, liquid consumed (determined by EC gravimetric weight change, pre-post vaping bout), and puffing topography records (i.e., puff duration, flow rate, and inter-puff interval). AUC from 0 to 160 min for the four 3-puff directed bouts will be estimated using a non-compartmental model and trapezoidal rule. All measures will be corrected for baseline values by subtracting the blood nicotine concentrations from the initial value (at the start of each bout).

### Trial I data analysis plan and sample size calculations

To account for the repeated Cmax (adjusted for baseline at time zero) assessments design and to evaluate the extent of within-subject correlation of Cmax outcomes, we will first compute using linear mixed effect models, the intra-class correlation coefficients for the Cmax measures, first separately for each of the forms (protonated and freebase) and then for all Cmax measurements from the same participant. This step will help us understand the magnitude within-subject correlation of Cmax measures and to compare the within-subject correlation for the two main forms. Next, for each of the forms, we will investigate how Cmax relates to changing nicotine flux, by using linear mixed effect models (accounting for the within-subject design) and flux conditions as a predictor; we will investigate flux conditions both categorically and continuously to detect whether the change in Cmax follows a specific linear trend or whether some flux concentrations relate differently to Cmax. We will use model fit criteria to select the correlation structure and representation of flux concentrations that best describes the data. This investigation will be complemented by splines and locally weighted scatterplot smoothing (LOWESS) graphs to visualize plots of Cmax changes over the flux values. These analyses will be performed separately for the protonated and freebase conditions to assess similarities and differences in how the form of nicotine is influencing the Cmax-flux concentration relationship. Finally, we will integrate the form effect by using linear mixed models with both concentration and form as predictors as well as an interaction term between form*concentration to investigate whether different form-concentration combinations influence Cmax differently. The same sequence of analysis will be completed for the other outcomes of interest: dCi/dt, Tmax, and AUC, to evaluate how the different fluxes and forms relate to the rate of rise of nicotine, the time to Cmax and to cumulative nicotine exposure over time. We will investigate the relationship between participants’ data, such as sex, age, history of smoking, and baseline dependency scores with our pharmacokinetic outcomes. We will also look at whether some of these characteristics, namely sex, change the relationship between nicotine flux and the outcomes. In an additional analysis, we will evaluate whether nicotine fluxes are related to changes in puffing intensity. We will also repeat the main analyses adjusting for puffing intensity.

For sample size calculation, assuming an alpha level of 0.05 and an ICC of 0.4 (which conservatively assumes only a modest correlation between Cmax measures within the same participant), we estimated that we would need a total sample size of 15 subjects to detect with 90% power a medium effect size of 0.4. With an anticipated higher ICC and a higher relationship between flux and Cmax, as suggested by our prior work, this design will be well-powered to investigate the relationship of Cmax and flux concentrations for each form separately, and over 88% power to detect an effect size 0.4 for the between and within group comparisons [i.e., form*concentration]. We will continue recruitment until we achieve the targeted sample size.

### Trial II methods

#### Recruitment and retention

**Fig 3:**
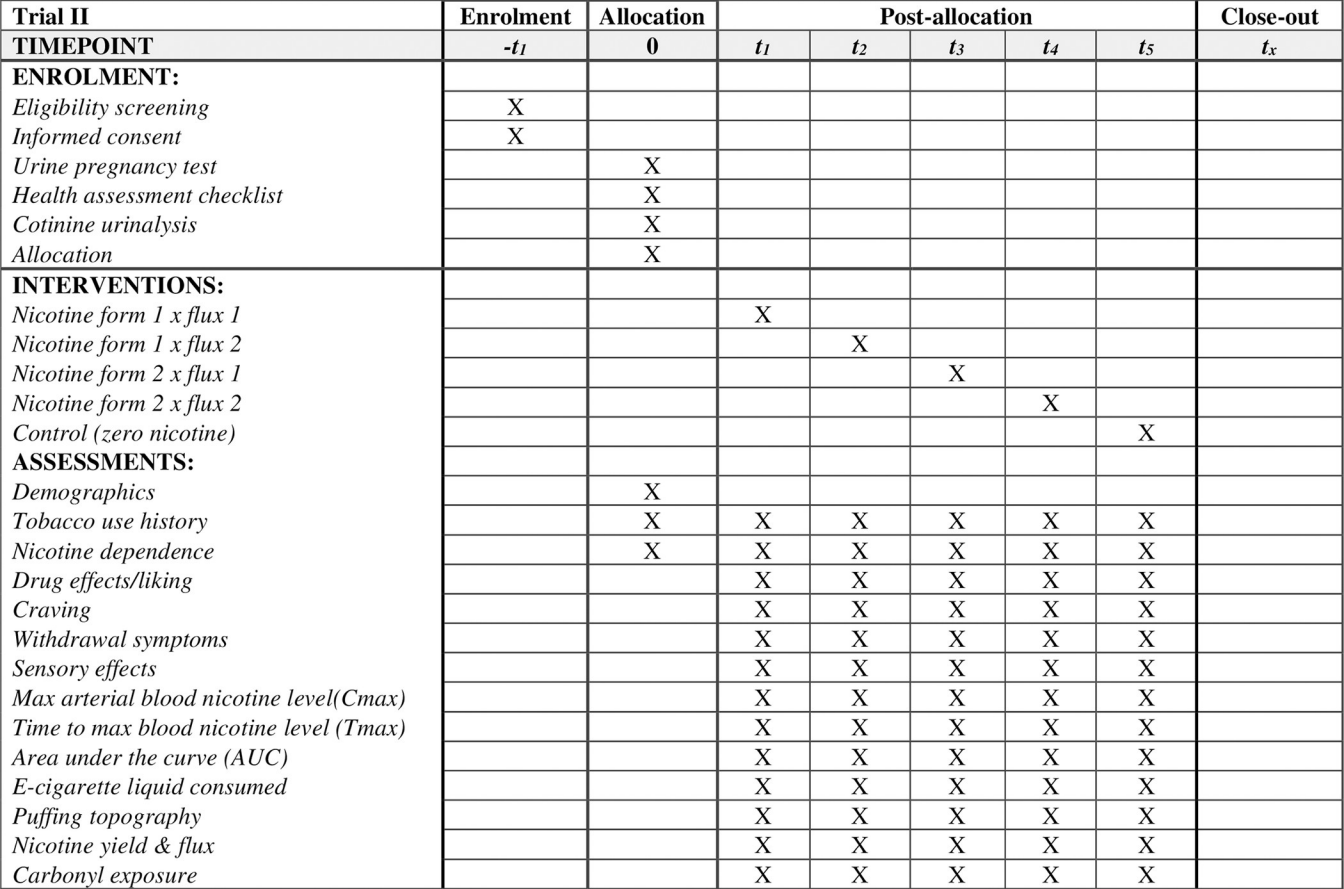
Standard protocol items: Recommendations for interventional trials (SPIRIT) recommended content for the schedule of enrolment, interventions, and assessments for Trial II.

For Trial II (currently recruiting), we estimated that we need 130 participants to detect the within-subject effect of nicotine flux*form on subjective effects related to dependence. We randomized flux*form for each participant and added a control placebo condition (Fig 3). To ensure retention, each participant will be paid $250 for a total of five visits (see below). The lower payment per session compared to Trial I is due to the absence of arterial blood sampling. Recruitment started at 07/13/2022 and is expected to end by 06/13/2024.

#### Trial design

Trial II will test the subjective effects of two nicotine fluxes (18 and 35 μg/sec) coupled with two ratios of nicotine form (100% freebase or 100% protonated). Additionally, a 0 mg/mL nicotine concentration condition will be used for comparison making a total of 5 conditions. Participants will be asked to puff on an EC device with the designated nicotine fluxes and forms, and subjective effects related to dependence will be measured. The EC device will be attached to a previously validated (NIDA 1R01DA025659) real-time in-situ sampling (RealTIME) device that unobtrusively samples the aerosol exiting the EC mouthpiece during each puff for subsequent chemical analysis; the device also records puffing topography (puff volume, duration, inter-puff interval). In this manner, mouth-level exposure to nicotine and pulmonary toxicants for each participant will be assessed. Liquid consumption for each participant use session will be also determined by pre- and post-weighing of the EC device.

#### Detailed study procedures

**Fig 4:**
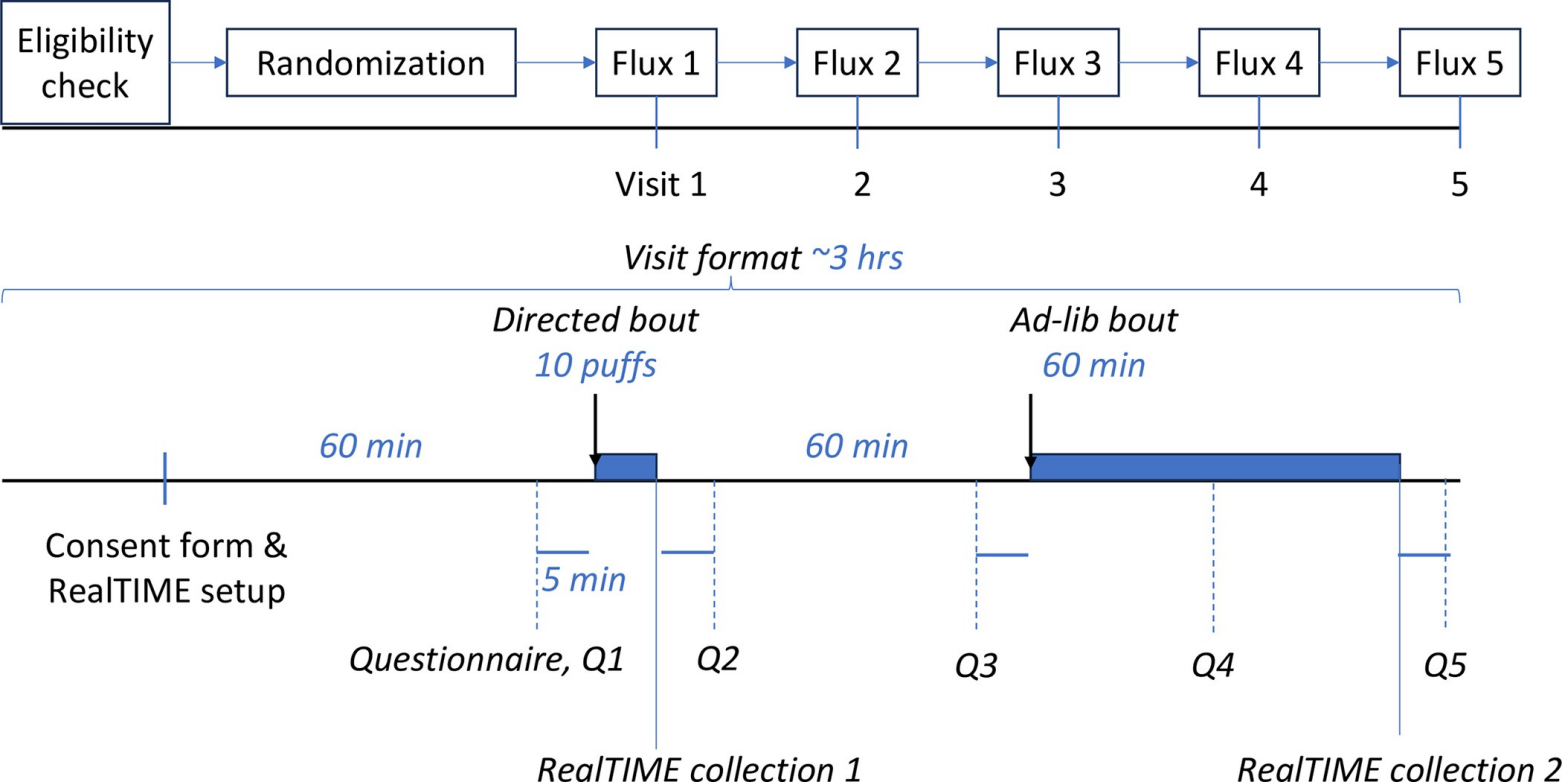
Trial II study design with time points to collect survey data and RealTIME samples.

Fig 4 summarizes the study visit design of Trial II. Each participant will attend the lab for five different visits that differ by nicotine flux and/or form in random order. All sessions will be double-blind for participants and research staff.

As in Trial I, before each visit, participants will be instructed to abstain from nicotine/tobacco and/or EC use. Following an hour-long observation period, participants will use Subox Mini C in a 10-puff directed (30sec inter-puff interval) bout [52, 57]. One hour after the first bout, participants will be instructed to puff on the device for 60-min ad libitum. Subjective measures (i.e., nicotine dependence, drug effects, product liking, and craving) will be administered 5 times/session: 5 min before and 5 min after the onset of the directed bout and 5 min before, halfway into, and 5 min after the ad libitum bout. Also, we will use RealTIME to trap a fraction of the aerosol emitted by the EC during each puff generated by the user, which allows us to examine the hypothesis that increasing flux (by solely increasing nicotine concentration not power; see [27]) will result in both reduced puffing intensity and reduced exposure to pulmonary toxicants.

### Trial II measures

In Trial II, the outcome measures include demographic data, subjective effects, puff topography records, amount of liquid consumed, nicotine (yield and flux), and carbonyl compound exposure. On the first visit to the clinic, participants will complete several surveys to assess their nicotine dependence. These include the PROMIS Nicotine Dependence Scale [58] which assesses the severity of nicotine dependence on cigarettes and the corresponding 4-item E-Cigarette Dependence Scale (EDS) which assesses dependence on ECs [59–61]. In addition, they will complete the Fagerström Test of Nicotine Dependence questionnaires [62]. Product liking, craving, and drug effects will be assessed on a subjective scale (0–100, not at all-extremely) immediately following product use. Product liking will be assessed by the Electronic Cigarette Specific Effects questionnaire [53], general labeled magnitude scale (gLMS) [63], and labeled hedonic scale (LHS) [64]. Craving (QSU) [65] and withdrawal symptoms (Minnesota Nicotine Withdrawal Scale) [66] will be assessed at baseline and following EC use. The Drug Effect Questionnaire (DEQ) will measure acute effects consisting of 7 items: drug strength, high, feeling stimulated, good effects, bad effects, wanting more drugs, and drug liking [56, 67].

### Trial II data analysis plan and sample size calculations

For the primary analysis of the flux-subjective effects relationship, we will first evaluate the intra- class correlation coefficients of subjective measures within each tested condition, then within forms (3 fluxes: 0, 18, and 35 ug/sec and 5 assessments for each condition), to inform the degree of correlation of subjective measures within the same flux condition and within the same form. We will then use a linear mixed effect model to assess how the subjective measures change across the 5 timepoints (i.e., the 5 measurements around the directed and ad libitum bouts) for each nicotine flux, across fluxes, and between forms. The analyses will look at each level separately and gradually build the significant levels/components of the model. They will also include a graphical representation of how subjective assessments change in time for each condition and across conditions (through splines and LOWESS plots of the subjective parameters in relation to time assessments for each flux condition, and in relation to the different concentrations for the various conditions tested for each form). The structure of correlations and modeling of the timing of the questionnaire for each condition will be assessed using model fit criteria. Similarly, predictors of interest, including sex, age, history of smoking, and baseline dependency scores will be investigated.

For sample size calculations, with alpha<0.05 and an ICC of 0.4, we estimated requiring 65 subjects to detect for each form, and with 90% power, a small effect size of 0.2 in the changes of subjective assessments for each form. To integrate both the repeated assessments for each form and the comparison between the two forms and with the 0 nicotine level condition, following a two-level design (the first level is the 5 repeated time point assessments within a condition, and the second level is the cluster of 5 conditions form/flux combinations per subject), we estimate a total sample size of 130 subjects to detect with 85% power an effect size of 0.5. We will continue recruitment until we achieve the targeted sample size.

### Research ethics approval

Institutional Review Board approval for Trial I and Trial II was obtained at YSM and AUB, respectively.

### Protocol amendments

Trial I was registered as NCT05706701 on ClinicalTrials.gov on Jan 31^st^, 2023, and last updated on Feb 16^th^, 2023. Trial II was registered as NCT05430334 on Jun 24^th^, 2022, and last updated on Aug 9^th^, 2023. Any amendment to the protocols will be approved by the corresponding institutional IRB and reflected in the registered protocol.

### Consent

All participants will be recruited under the guidelines of the AUB and Yale IRBs. The nature of the procedures, the risks, and the financial remuneration for participation in the study will be discussed with everyone before obtaining informed written consent by trained research staff under the supervision of Drs. Talih, Baldassarri, and El-Hellani (project leaders). The study team will always be available to address any questions that participants may have during the consent procedure and their participation in the study. Subjects are also “tested” on their knowledge of the study before participation.

### Confidentiality

The project leaders will be responsible for monitoring the data, assuring protocol compliance, and conducting the safety reviews at the specified frequency every 6 months. REDCap software will be used for secure data storage and analysis. Project leaders will prepare data safety monitoring reports twice a year, which include information on enrollment, participant retention rates, adverse events, preliminary analyses, and data safety. During the review process, the leaders will evaluate whether the study should continue unchanged, require modification/amendment, or close the enrollment. All subject information will be kept confidential and only members of the investigative team with appropriate IRB and HIPAA training will have access to the study data. Data will be maintained and secured in locked file cabinets or password-protected electronic media. A numbering code will be used to assign a unique identifier to each participant. This information is available only to study investigators.

## Supporting information

S1 Checklist Recommended items to address in a clinical trial protocol and related documents. (DOCX)

S1 Appendix Yale school of medicine IRB consent. (DOCX)

S2 Appendix Yale school of medicine IRB protocol. (DOCX)

S3 Appendix American University of Beirut consent. (PDF)

S4 Appendix American University of Beirut protocol. (PDF)

S1 Fig Nicotine flux from different generations of e-cigarettes (ECs) and other nicotine-delivering products. Generations of e-cigarettes were determined according to.
(TIF)

## References

[1] Besaratinia A, Tommasi S. Vaping: A growing global health concern. *EClinicalMedicine*. 2019;17:100208. doi: 10.1016/j.eclinm.2019.10.019 31891141 PMC6933142

[2] WHO. WHO report on the global tobacco epidemic 2021: addressing new and emerging products. Geneva; 2021. Contract No.: Licence: CC BY-NC-SA 3.0 IGO.

[3] CDC. QuickStats: Percentage Distribution of Cigarette Smoking Status Among Current Adult E-Cigarette Users, by Age Group—National Health Interview Survey, United States, 2021. *MMWR Morb Mortal Wkly Rep*. 2023;72:270. 10.15585/mmwr.mm7210a7. 36893067 PMC10010756

[4] Park-Lee E, Ren C, Cooper M, et al. Tobacco Product Use Among Middle and High School Students—United States, 2022. *MMWR Morb Mortal Wkly Rep*. 2022;71:1429–35. doi: 10.15585/mmwr.mm7145a1 36355596 PMC9707354

[5] Davis DR, Morean ME, Bold KW, et al. Cooling e-cigarette flavors and the association with e-cigarette use among a sample of high school students. *PLoS One*. 2021;16(9):e0256844. doi: 10.1371/journal.pone.0256844 34469460 PMC8409641

[6] Leventhal AM, Goldenson NI, Cho J, et al. Flavored E-cigarette Use and Progression of Vaping in Adolescents. *Pediatrics*. 2019;144(5). doi: 10.1542/peds.2019-0789 31659004 PMC6856781

[7] Barrington-Trimis JL, Leventhal AM. Adolescents’ Use of "Pod Mod" E-Cigarettes—Urgent Concerns. *N Engl J Med*. 2018;379(12):1099–102. doi: 10.1056/NEJMp1805758 30134127 PMC7489756

[8] Kong G, Bold KW, Morean ME, et al. Appeal of JUUL among adolescents. *Drug Alcohol Depend*. 2019;205:107691. doi: 10.1016/j.drugalcdep.2019.107691 31706249 PMC7307218

[9] Kinnunen JM, Ollila H, Minkkinen J, et al. Nicotine matters in predicting subsequent smoking after e-cigarette experimentation: A longitudinal study among Finnish adolescents. *Drug Alcohol Depend*. 2019;201:182–7. doi: 10.1016/j.drugalcdep.2019.04.019 31238240

[10] Camenga DR, Morean M, Kong G, et al. Appeal and Use of Customizable E-cigarette Product Features in Adolescents. *Tob Regul Sci*. 2018;4(2):51–60. doi: 10.18001/trs.4.2.5 33163582 PMC7643857

[11] Chaffee BW, Couch ET, Wilkinson ML, et al. Flavors Increase Adolescents’ Willingness to Try Nicotine and Cannabis Vape Products. *Drug and Alcohol Dependence*. 2023:109834. doi: 10.1016/j.drugalcdep.2023.109834 36963159 PMC10121941

[12] Tackett AP, Dai HD, Han D-H, et al. Appeal of e-cigarette flavors: Differences between never and ever use of combustible cigarettes. *Drug and Alcohol Dependence*. 2023;246:109849. doi: 10.1016/j.drugalcdep.2023.109849 37028103 PMC10161874

[13] Alhadyan SK, Sivaraman V, Onyenwoke RU. E-cigarette Flavors, Sensory Perception, and Evoked Responses. *Chem Res Toxicol*. 2022. doi: 10.1021/acs.chemrestox.2c00268 36480683

[14] Voos N, Goniewicz ML, Eissenberg T. What is the nicotine delivery profile of electronic cigarettes? *Expert Opin Drug Deliv*. 2019;16(11):1193–203. doi: 10.1080/17425247.2019.1665647 31495244 PMC6814574

[15] Eissenberg T. Electronic nicotine delivery devices: ineffective nicotine delivery and craving suppression after acute administration. *Tob Control*. 2010;19(1):87–8. doi: 10.1136/tc.2009.033498 20154061 PMC3208854

[16] Dawkins L, Kimber C, Puwanesarasa Y, et al. First- versus second-generation electronic cigarettes: predictors of choice and effects on urge to smoke and withdrawal symptoms. *Addiction*. 2015;110(4):669–77. doi: 10.1111/add.12807 25407505

[17] Hajek P, Pittaccio K, Pesola F, et al. Nicotine delivery and users’ reactions to Juul compared with cigarettes and other e-cigarette products. *Addiction*. 2020;115(6):1141–8. doi: 10.1111/add.14936 31994254 PMC7318270

[18] Eversole A, Budd S, Karaoghlanian N, et al. Interactive effects of protonated nicotine concentration and device power on ENDS nicotine delivery, puff topography, and subjective effects. *Exp Clin Psychopharmacol*. 2022. doi: 10.1037/pha0000576 35696157 PMC10082447

[19] Breland A, Maloney SF, Soule EK, et al. Abuse liability of electronic cigarettes in men who are experienced electronic cigarette users. *Exp Clin Psychopharmacol*. 2020;28(2):235–44. doi: 10.1037/pha0000305 31259592 PMC6938579

[20] Gades MS, Alcheva A, Riegelman AL, et al. The Role of Nicotine and Flavor in the Abuse Potential and Appeal of Electronic Cigarettes for Adult Current and Former Cigarette and Electronic Cigarette Users: A Systematic Review. *Nicotine & Tobacco Research*. 2022;24(9):1332–43. doi: 10.1093/ntr/ntac073 35305014 PMC9356694

[21] Lin C, Gaiha SM, Halpern-Felsher B. Nicotine Dependence from Different E-Cigarette Devices and Combustible Cigarettes among US Adolescent and Young Adult Users. *Int J Environ Res Public Health*. 2022;19(10). doi: 10.3390/ijerph19105846 35627381 PMC9140375

[22] El Hourani M, Shihadeh A, Talih S, et al. (Nicotine flux working group including A El-Hellani and TL Wagener). Comparison of nicotine emissions rate, ’nicotine flux’, from heated, electronic and combustible tobacco products: data, trends and recommendations for regulation. *Tob Control*. 2022. doi: 10.1136/tobaccocontrol-2021-056850 35086911 PMC9325916

[23] EU. Directive 2014/40/EU of the European Parliament and of the Council of 3 April 2014 on the approximation of the laws, regulations and administrative provisions of the Member States concerning the manufacture, presentation and sale of tobacco and related products and repealing Directive 2001/37/EC Text with EEA relevance. 2014. Contract No.: Document 32014L0040.

[24] Canada. Nicotine Concentration in Vaping Products Regulations. 2021. Contract No.: SOR/2021-123.

[25] Ali FRM, Schillo B, Crane E, et al. Evaluation of Statewide Restrictions on E-Cigarette Nicotine Strength- United States, 2017–2022. *Addiction*. 2023. doi: 10.1111/add.16206 37039371

[26] Eissenberg T, Soule E, Shihadeh A. ’Open-System’ electronic cigarettes cannot be regulated effectively. *Tobacco Control*. 2021;30(2):234–5. doi: 10.1136/tobaccocontrol-2019-055499 32184338 PMC7848783

[27] Talih S, Salman R, El-Hage R, et al. Might limiting liquid nicotine concentration result in more toxic electronic cigarette aerosols? *Tob Control*. 2021;30(3):348–50. doi: 10.1136/tobaccocontrol-2019-055523 32522818 PMC9281877

[28] Shihadeh A, Eissenberg T. Electronic cigarette effectiveness and abuse liability: predicting and regulating nicotine flux. *Nicotine Tob Res*. 2015;17(2):158–62. doi: 10.1093/ntr/ntu175 25180079 PMC4837999

[29] Eissenberg T, Shihadeh A. Nicotine flux: a potentially important tool for regulating electronic cigarettes. *Nicotine Tob Res*. 2015;17(2):165–7. doi: 10.1093/ntr/ntu208 25332456 PMC4838002

[30] Duell AK, Pankow JF, Peyton DH. Nicotine in tobacco product aerosols: ‘It’s déjà vu all over again’. *Tobacco Control*. 2020;29(6):656–62. doi: 10.1136/tobaccocontrol-2019-055275 31848312 PMC7591799

[31] Pierce JP, Leas EC, Strong DR. Biochemical Validation of Dependence on JUUL and Other E-Cigarettes Among Youth. *Pediatrics*. 2023;151(4). doi: 10.1542/peds.2022-059158 36942497

[32] Han S, Liu C, Chen H, et al. Pharmacokinetics of freebase nicotine and nicotine salts following subcutaneous administration in male rats. *Drug Testing and Analysis*.n/a(n/a). doi: 10.1002/dta.3363 36059224

[33] Benowitz NL. The Central Role of pH in the Clinical Pharmacology of Nicotine: Implications for Abuse Liability, Cigarette Harm Reduction and FDA Regulation. *Clin Pharmacol Ther*. 2022;111(5):1004–6. doi: 10.1002/cpt.2555 35220591 PMC9035094

[34] Allain F, Minogianis EA, Roberts DC, et al. How fast and how often: The pharmacokinetics of drug use are decisive in addiction. *Neurosci Biobehav Rev*. 2015;56:166–79. doi: 10.1016/j.neubiorev.2015.06.012 26116543

[35] Berridge MS, Apana SM, Nagano KK, et al. Smoking produces rapid rise of [11C]nicotine in human brain. *Psychopharmacology (Berl)*. 2010;209(4):383–94. doi: 10.1007/s00213-010-1809-8 20232056

[36] Solingapuram Sai KK, Zuo Y, Rose JE, et al. Rapid Brain Nicotine Uptake from Electronic Cigarettes. *J Nucl Med*. 2020;61(6):928–30. doi: 10.2967/jnumed.119.230748 31676729 PMC8008769

[37] Talih S, Balhas Z, Salman R, et al. Transport phenomena governing nicotine emissions from electronic cigarettes: model formulation and experimental investigation. *Aerosol Sci Technol*. 2017;51(1):1–11. doi: 10.1080/02786826.2016.1257853 28706340 PMC5502764

[38] El-Hellani A, El-Hage R, Baalbaki R, et al. Free-Base and Protonated Nicotine in Electronic Cigarette Liquids and Aerosols. *Chem Res Toxicol*. 2015;28(8):1532–7. doi: 10.1021/acs.chemrestox.5b00107 26158618 PMC4920054

[39] Talih S, Salman R, El-Hage R, et al. Effect of free-base and protonated nicotine on nicotine yield from electronic cigarettes with varying power and liquid vehicle. *Sci Rep*. 2020;10(1):16263. doi: 10.1038/s41598-020-73385-6 33004992 PMC7530983

[40] Talih S, Balhas Z, Eissenberg T, et al. Effects of User Puff Topography, Device Voltage, and Liquid Nicotine Concentration on Electronic Cigarette Nicotine Yield: Measurements and Model Predictions. *Nicotine & Tobacco Research*. 2014;17(2):150–7. doi: 10.1093/ntr/ntu174 25187061 PMC4837998

[41] Baldassarri SR, Hillmer AT, Anderson JM, et al. Use of Electronic Cigarettes Leads to Significant Beta2-Nicotinic Acetylcholine Receptor Occupancy: Evidence From a PET Imaging Study. *Nicotine Tob Res*. 2018;20(4):425–33. doi: 10.1093/ntr/ntx091 28460123 PMC5896427

[42] El-Hellani A, Salman R, El-Hage R, et al. Nicotine and Carbonyl Emissions From Popular Electronic Cigarette Products: Correlation to Liquid Composition and Design Characteristics. *Nicotine Tob Res*. 2018;20(2):215–23. doi: 10.1093/ntr/ntw280 27798087 PMC5896517

[43] Talih S, Salman R, El-Hage R, et al. Characteristics and toxicant emissions of JUUL electronic cigarettes. *Tob Control*. 2019;28(6):678–80. doi: 10.1136/tobaccocontrol-2018-054616 30745326 PMC7341718

[44] Goel R, Trushin N, Reilly SM, et al. A Survey of Nicotine Yields in Small Cigar Smoke: Influence of Cigar Design and Smoking Regimens. *Nicotine Tob Res*. 2018;20(10):1250–7. doi: 10.1093/ntr/ntx220 29059441 PMC6121918

[45] Bodnar JA, Morgan WT, Murphy PA, et al. Mainstream smoke chemistry analysis of samples from the 2009 US cigarette market. *Regul Toxicol Pharmacol*. 2012;64(1):35–42. doi: 10.1016/j.yrtph.2012.05.011 22683394

[46] Rickert WS, Robinson JC, Bray DF, et al. Characterization of tobacco products: a comparative study of the tar, nicotine, and carbon monoxide yields of cigars, manufactured cigarettes, and cigarettes made from fine-cut tobacco. *Prev Med*. 1985;14(2):226–33. doi: 10.1016/0091-7435(85)90038-6 4048085

[47] Salman R, Talih S, El-Hage R, et al. Free-Base and Total Nicotine, Reactive Oxygen Species, and Carbonyl Emissions From IQOS, a Heated Tobacco Product. *Nicotine & Tobacco Research*. 2019;21(9):1285–8. doi: 10.1093/ntr/nty235 30476301 PMC6698952

[48] Bekki K, Inaba Y, Uchiyama S, et al. Comparison of Chemicals in Mainstream Smoke in Heat-not-burn Tobacco and Combustion Cigarettes. *J uoeh*. 2017;39(3):201–7. doi: 10.7888/juoeh.39.201 28904270

[49] Shihadeh A, Saleh R. Polycyclic aromatic hydrocarbons, carbon monoxide, "tar", and nicotine in the mainstream smoke aerosol of the narghile water pipe. *Food Chem Toxicol*. 2005;43(5):655–61. doi: 10.1016/j.fct.2004.12.013 15778004

[50] Felicione NJ, Fix BV, McNeill A, et al. Characteristics and changes over time of nicotine vaping products used by vapers in the 2016 and 2018 ITC Four Country Smoking and Vaping Surveys. *Tobacco Control*. 2022;31(e1):e66–e73. doi: 10.1136/tobaccocontrol-2020-056239 33753550 PMC8455705

[51] Chan AW, Tetzlaff JM, Altman DG, et al. SPIRIT 2013 statement: defining standard protocol items for clinical trials. *Ann Intern Med*. 2013;158(3):200–7. doi: 10.7326/0003-4819-158-3-201302050-00583 23295957 PMC5114123

[52] Spindle TR, Talih S, Hiler MM, et al. Effects of electronic cigarette liquid solvents propylene glycol and vegetable glycerin on user nicotine delivery, heart rate, subjective effects, and puff topography. *Drug Alcohol Depend*. 2018;188:193–9. doi: 10.1016/j.drugalcdep.2018.03.042 29778773 PMC7193252

[53] Hiler M, Karaoghlanian N, Talih S, et al. Effects of electronic cigarette heating coil resistance and liquid nicotine concentration on user nicotine delivery, heart rate, subjective effects, puff topography, and liquid consumption. *Exp Clin Psychopharmacol*. 2020;28(5):527–39. doi: 10.1037/pha0000337 31855003 PMC9159736

[54] Solingapuram Sai KK, Rose JE, Mukhin AG. Effect Of Electronic Cigarette Liquid Ph On Retention Of 11c-Nicotine In A Respiratory Tract Model. *Nicotine & Tobacco Research*. 2023. doi: 10.1093/ntr/ntad039 36905343 PMC10256878

[55] CORESTA. CORESTA Recommended Method N 81. Routine Analytical Machine for E-cigarette Aerosol Generation and Collection-Definitions and Standard Conditions 2015 [Available from: https://www.coresta.org/routine-analytical-machine-e-cigarette-aerosol-generation-and-collection-definitions-and-standard.

[56] Sofuoglu M, Herman AI, Nadim H, et al. Rapid nicotine clearance is associated with greater reward and heart rate increases from intravenous nicotine. *Neuropsychopharmacology*. 2012;37(6):1509–16. doi: 10.1038/npp.2011.336 22334123 PMC3327855

[57] Lopez AA, Hiler MM, Soule EK, et al. Effects of Electronic Cigarette Liquid Nicotine Concentration on Plasma Nicotine and Puff Topography in Tobacco Cigarette Smokers: A Preliminary Report. *Nicotine Tob Res*. 2016;18(5):720–3. doi: 10.1093/ntr/ntv182 26377515 PMC5896822

[58] Edelen MO. The PROMIS smoking assessment toolkit—background and introduction to supplement. *Nicotine Tob Res*. 2014;16 Suppl 3(Suppl 3):S170–4. doi: 10.1093/ntr/ntu086 25118225 PMC4189410

[59] Morean ME, Krishnan-Sarin S, S SOM. Assessing nicotine dependence in adolescent E-cigarette users: The 4-item Patient-Reported Outcomes Measurement Information System (PROMIS) Nicotine Dependence Item Bank for electronic cigarettes. *Drug Alcohol Depend*. 2018;188:60–3. doi: 10.1016/j.drugalcdep.2018.03.029 29753155 PMC6983293

[60] Morean ME, Krishnan-Sarin S, Sussman S, et al. Psychometric Evaluation of the E-cigarette Dependence Scale. *Nicotine Tob Res*. 2019;21(11):1556–64. doi: 10.1093/ntr/ntx271 29301008 PMC6821314

[61] Morean ME, Krishnan-Sarin S, Sussman S, et al. Psychometric evaluation of the Patient-Reported Outcomes Measurement Information System (PROMIS)Nicotine Dependence Item Bankfor use with electronic cigarettes. *Nicotine Tob Res*. 2020;22(11):2123. doi: 10.1093/ntr/ntz095 31254382 PMC7593357

[62] Heatherton TF, Kozlowski LT, Frecker RC, et al. The Fagerström Test for Nicotine Dependence: a revision of the Fagerström Tolerance Questionnaire. *Br J Addict*. 1991;86(9):1119–27. doi: 10.1111/j.1360-0443.1991.tb01879.x 1932883

[63] Rosbrook K, Green BG. Sensory Effects of Menthol and Nicotine in an E-Cigarette. *Nicotine Tob Res*. 2016;18(7):1588–95. doi: 10.1093/ntr/ntw019 26783293 PMC4902888

[64] Lim J, Wood A, Green BG. Derivation and evaluation of a labeled hedonic scale. *Chem Senses*. 2009;34(9):739–51. doi: 10.1093/chemse/bjp054 19833660 PMC2762053

[65] Tiffany ST, Drobes DJ. The development and initial validation of a questionnaire on smoking urges. *Br J Addict*. 1991;86(11):1467–76. doi: 10.1111/j.1360-0443.1991.tb01732.x 1777741

[66] Hughes JR, Hatsukami D. Signs and symptoms of tobacco withdrawal. *Arch Gen Psychiatry*. 1986;43(3):289–94. doi: 10.1001/archpsyc.1986.01800030107013 3954551

[67] Morean ME, de Wit H, King AC, et al. The drug effects questionnaire: psychometric support across three drug types. *Psychopharmacology (Berl)*. 2013;227(1):177–92. doi: 10.1007/s00213-012-2954-z 23271193 PMC3624068

