# Supplementary material for: Nicotine flux as a powerful tool for regulating nicotine delivery from e-cigarettes: Protocol of two complimentary randomized crossover clinical trials
Source: PLoS One. 2023 Sep 21;18(9):e0291786. doi: 10.1371/journal.pone.0291786 (PMC10513228; doi:10.1371/journal.pone.0291786)
Supplement: S1 Appendix — (DOCX) [file pone.0291786.s002.docx]

### COMPOUND AUTHORIZATION AND CONSENT FOR PARTICIPATION IN A RESEARCH STUDY

**YALE UNIVERSITY SCHOOL OF MEDICINE**

**Study Title:** **Nicotine flux, a potentially powerful tool for regulating nicotine delivery from electronic cigarettes**

**Principal Investigator (the person who is responsible for this research):**

Stephen Baldassarri, MD, MHS

300 Cedar St, TAC 455-S, New Haven, CT 06520

Phone Number: (203) 824-9215

**Research Study Summary:**

# • We are asking you to join a research study.

# • The purpose of this research study is to understand factors that affect nicotine delivery from e-cigarettes.

# • Study procedures will include vaping e-cigarettes, arterial line placement, blood draws, written assessments.

# • 3 visits are required, including 1 screening visit.

# • These visits will take 13 hours total.

# • There are some risks from participating in this study. These risks are related to e-cigarette use, arterial line placement, blood drawing, and data privacy. The risks are described in detail below.

# • This study can benefit you and others by advancing your knowledge of the factors that influence nicotine delivery from e-cigarettes.

• The alternative is not to participate.

# • Taking part in this study is your choice. You can choose to take part, or you can choose not to take part in this study. You can also change your mind at any time. Whatever choice you make, you will not lose access to your medical care or give up any legal rights or benefits.

# • If you are interested in learning more about the study, please continue reading, or have someone read to you, the rest of this document. Take as much time as you need before you make your decision. Ask the study staff questions about anything you do not understand. Once you understand the study, we will ask you if you wish to participate; if so, you will have to sign this form.

Description for Study Participants

# The purpose of this research study is to understand factors that affect nicotine delivery from e-cigarettes. We want to include you in this study because you are healthy and currently use nicotine e-cigarettes. 15 participants will be enrolled. This study will involve 2 sessions, during which time you will vape e-cigarettes and will have blood drawn. The benefits, risks, and alternatives of participating in this study are described in detail below. You will be compensated appropriately for your time spent completing each part of the study as described below. A Research Assistant or the Principal Investigator will also describe the study procedures to you verbally in detail to make sure you fully understand the study.

**Why is this study being offered to me?**

We are asking you to take part in a research study because you are experienced with e-cigarette use or vaping. Since this study involves use of e-cigarettes, you are a candidate for participation.

**Who is paying for the study?**

National Institutes of Health / National Institute on Drug Abuse

**Who is providing other support for the study?**

N/A.

**What is the study about?**

The purpose of this study is to understand factors that affect nicotine delivery from e-cigarettes.

**What are you asking me to do and how long will it take?**

If you agree to take part in this study, the steps below will occur.

First, you will attend a screening visit that will last less than 1 hour. We will assess your use of e-cigarettes, other nicotine products, and your overall health for safe participation and to get a baseline for future health checks. The following study procedures will take place:

- Health questionnaires: These will establish any health risks prior to participating.
- Spirometry: This is a routine procedure that involves blowing forcefully into a tube and measuring airflow. It tests whether the airways are open or narrowed.
- Physical Exam: This is a routine procedure performed by a study physician that assesses for any major abnormalities in the heart, lungs, abdomen, extremities, and skin.
- Collection of smoking and vaping history
- Women will receive pregnancy testing at screening and before each subsequent study visit

Next, we ask you to come for a study visit on 2 different days, approximately 1-4 weeks apart. Each day will last approximately 6 hours, and you will be paid for your participation after completion of all study procedures.

After you sign informed consent, you will come to the Human Research Unit, which is our dedicated space for research studies. We will secure your valuables and help you get settled into your room. We will ask you to abstain from nicotine use for 1 hour prior to your visit. You will meet the Research Nurse, who will take care of you throughout the day, monitor your progress, and make sure things run smoothly.

Next, one of our study physicians will place an arterial catheter into your non-dominant wrist (radial artery). The arterial catheter will allow us to draw very small amounts of your blood after vaping different types of e-cigarettes. Once the catheter has been placed, blood can be drawn frequently and in small amounts without causing any pain or discomfort. The arterial catheter is about 2 inches long and looks like a regular IV tube, but it is inserted into an artery, not a vein. The blood flow in the arteries can tell us about your blood pressure. If an arterial catheter is in place, we can measure your blood pressure continuously. Thanks to an arterial catheter, we can draw blood quickly, more than once, and without causing you pain. Here is what happens when an arterial line is placed:

- We will clean the skin with betadine solution (contains iodine). It will reduce the risk of an infection.
- We will numb your skin with a local anesthetic so that you feel less pain when the catheter is inserted. You will probably just feel pressure but may also feel pain. It would be similar to the pain you feel with an IV.
- We will flush the catheter often during your scan with saline (a salt solution) to make sure it does not clog.
- After we remove the catheter, we will apply pressure to your skin for a minimum of 15 minutes to prevent bleeding under the skin.
- We will apply a pressure dressing (coban) and clear dressing (tegaderm). You will need to keep it clean and dry. Do not exercise too much and do not lift heavy objects weighing more than 5 pounds. Avoid making the same movements for 48 hours.

You may remove the pressure dressing at bedtime and the clear dressing after 48 hours, but do not put your hand and wrist in water for a full 72 hours. Since the catheter is in for a minimal period, there is a low risk of infection.

Once the arterial catheter is placed, we are ready to get started with the experiment. We will ask you to vape 4 different nicotine e-cigarettes (2, 4, 7 and 10mg/ml), taking 3 puffs at a time. Each series of 3 e-cig puffs is a “bout.” 11 - 22 cc of your blood will be drawn from the arterial line during and after each “bout,” and there will be roughly an hour in between the 4 “bouts.” The nicotine level in blood will be measured. After each bout, the Research Assistant will administer assessments. For example, she will ask you questions about how much you like that particular e-cigarette, how it makes you feel, and whether you have any further desire to use the e-cigarette.

After all of the bouts are complete, the Research Nurse will remove the arterial line and will hold pressure for several minutes to prevent bleeding and will then apply a dressing to the skin that can be removed after about 12 hours. You will be discharged from the research unit to return home. You will visit a 2^nd^ time and repeat this process. You will then receive your payment for participating and completing the study. This study does not store samples for future research and biospecimens will not be used for commercial profit. The research will not include whole genome sequencing or genetic testing.

**What are my responsibilities as a participant in this study?**

- **Attend a screening visit and 2 lab visits**
- **Complete all study procedures**
- **Report any adverse events to the study team**

**What are the risks and discomforts of participating?**

***Risks Associated with Use of an Arterial Line.***

**Important: You cannot take part in the study, if you have ever had a bleeding disorder or are taking medication to thin your blood.**

**The insertion of the arterial line may be painful and you can get bruises. The arterial puncture may cause a spasm, a temporary tightening (constriction) of the muscles in the wall of the artery. You may get a clot and your blood flow will slow down for a little while. You can get a hematoma (swelling of blood within the tissues). The site can bleed or get inflamed (become red, swollen, hot, and painful). These feelings will go away after some time, usually 24 to 72 hours after the procedure. Rarely, you may experience blocking of the artery or nerve damage to the insertion site. The insertion site may not heal as fast or you may get infection. This is why an experienced health care provider will insert the arterial line and a trained nurse will look after for you. Check your wrist and arm every day for two days after the study visit with the arterial line. Seek immediate medical attention (i.e., call your primary care physician’s office and/or, go to a local Hospital or Urgent Care Center for an immediate evaluation if you notice any of the following:**

- **You feel a lot of pain**
- **Your wrist or arm is tender, swollen, or red**
- **You see some blood or other fluids coming out of the injection site**
- **The color of your skin changes**
- **Your arms feel numb**
- **You feel pins and needles in your arm**
- **Your arm that had the catheter does not feel as strong.**

**After you seek medical attention, please call the Study Team and Prinicipal Investigator, Dr. Stephen Baldassarri, at 203-824-9215 to report an adverse reaction and any care sought and received.**

**Tell us if you have had a bad reaction to lidocaine, novocain, or other drugs used to numb the skin in the past. You may experience a rare allergic reaction to the medicine used to numb your skin prior to placement of the arterial catheter. Severe allergic reactions can be life threatening. Some things that happen during an allergic reaction are:**

- **rash**
- **hives**
- **having a hard time breathing**
- **wheezing when you breathe**
- **sudden drop in blood pressure**
- **swelling around the face, mouth, lips, tongue, throat, or eyes**
- **fast pulse**
- **sweating**

**If you have any of the above allergy related side effects or symptoms, your study doctor will assess you and treat these symptoms.**

**Do not take aspirin and other anti-inflammatory drugs (such as Motrin or Aleve) for 7-10 days before arterial line placement and 7-10 days after the study visit.**

**Contact the Principal Investigator at 203-824-9215 with any concerns.**

***Risks Associated with Blood Drawing***

You should not donate blood for at least 8 weeks after the study. The total volume of blood collected during this study will be 44 - 88 cc (9 - 18 teaspoons). This amount of blood is safe for healthy persons.

***Risks Associated with E-Cigarette Use***

Over various Committee reviews of e-cigarette studies during EVALI and COVID-19, the IRB along with the PI’s, has developed standard criteria for risk considerations in these studies where the lungs may be uniquely affected.

- Risk of the device overheating, fire, or explosion to the device. This is extremely rare. Staff are educated on proper device inspection, operation, charging/recharging, storage, and final disposition.
- Although there is much research devoted to EVALI, the exact cause of this lung injury remains unknown. Therefore, we ask you to abstain from using tobacco and THC-containing products from unknown sources, as they may not comply with standards set by Good Manufacturing Practices and could contain contaminants. The symptoms of EVALI can include cough, shortness of breath, chest pain, nausea, vomiting, stomach pain, diarrhea, fever, chills, or weight loss. If you feel sick or experience any of these symptoms, contact the study team as soon as possible. In this study we monitor for the symptoms of EVALI, which is a direct benefit for those who may be at risk for developing this condition. There are no other direct benefits of this study.
- We will assess your health at the intake to make sure you are healthy prior to participating and will continue to monitor your health closely during the study. If you experience any symptoms (such as abdominal pain, nausea, vomiting, diarrhea, cough, shortness of breath, chest pain) or other concerns, please let us know and let your doctor know promptly (right away).   Go to the emergency room if your symptoms increase.  You can stop the study at any point. If you feel any discomfort or need to stop for any reason, please let the research team know.
- E-cigarette liquid contain other chemicals besides nicotine including propylene glycol/vegetable glycol/vegetable glycerin. At this time, we do not know the risks associated with the propylene glycol/ vegetable glycerin that may in the fillers in the liquids used in this study.  Benzoic acid is also present and is part of commercially available e-liquids.
- It is important to note that there may be unforeseen risks (such as allergic reactions). Some research has indicated that in large doses propylene glycol and vegetable glycerin can be harmful. However, if you experience any side effects, you can stop the session at any point. Research staff will monitor e-cigarette use during the lab session. If you feel any discomfort or need to stop for any reason, please let the researcher know. The strength and concentration levels of nicotine may cause potential forseeable unpleasant adverse reactions.
- The risks linked to the use of e-liquid flavors may be related to a regular and longer-term use. They include the potential for burns to and scarring of the respiratory system. There are also risks related to the chemicals used in the flavors and the effect of heat on the flavors. Another possible risk of the flavored e-cigarettes is a higher potential for nicotine addiction and continued use of the e-cigarettes. In this study, the use is limited to [24 puffs] of a flavored cigarette.

***The CDC has warned against vaping e-cigarettes***

There have been recent reported cases of severe lung (pulmonary) illness linked to “vaping” or e-cigarette use. These cases included symptoms such as coughing, shortness of breath, chest pain, fever, fatigue, nausea, vomiting, diarrhea, and/or abdominal pain. Some patients reported symptoms to have occurred over a few days and some reported to have occurred over a few weeks. Vaping-related disorders have ranged from mild to severe with hospitalization, intensive care with breathing machines and in some cases death.  In most cases, but not all, people experiencing these symptoms were using cannabidiol (CBD) or marijuana (THC) e-liquids, and/or were using e-cigarette devices and e-liquids that were mixed at home or purchased off market (such as purchasing an e-liquid or device on the street, not from a licensed retailer).

The Center for Disease Control ([www.cdc.gov](https://nam12.safelinks.protection.outlook.com/?url=http%3A%2F%2Fwww.cdc.gov%2F&data=04%7C01%7Cstephen.baldassarri%40yale.edu%7C7a683a4d33304fcce38a08d9f01ab290%7Cdd8cbebb21394df8b4114e3e87abeb5c%7C0%7C0%7C637804820067002900%7CUnknown%7CTWFpbGZsb3d8eyJWIjoiMC4wLjAwMDAiLCJQIjoiV2luMzIiLCJBTiI6Ik1haWwiLCJXVCI6Mn0%3D%7C3000&sdata=tDZsPSXjrZY52JC2AJYmMaJkBLb3BmjTn8BC8JKUuLA%3D&reserved=0)) has issued the following information on vaping:

- The use of e-cigarettes is unsafe for kids, teens, and young adults.
- Most e-cigarettes contain nicotine. Nicotine is highly addictive and can harm adolescent brain development, which continues into the early to mid-20s.[^*^](https://nam12.safelinks.protection.outlook.com/?url=https%3A%2F%2Fwww.cdc.gov%2Ftobacco%2Fbasic_information%2Fe-cigarettes%2FQuick-Facts-on-the-Risks-of-E-cigarettes-for-Kids-Teens-and-Young-Adults.html%23one&data=04%7C01%7Cstephen.baldassarri%40yale.edu%7C7a683a4d33304fcce38a08d9f01ab290%7Cdd8cbebb21394df8b4114e3e87abeb5c%7C0%7C0%7C637804820067002900%7CUnknown%7CTWFpbGZsb3d8eyJWIjoiMC4wLjAwMDAiLCJQIjoiV2luMzIiLCJBTiI6Ik1haWwiLCJXVCI6Mn0%3D%7C3000&sdata=rC%2Bz9szwbofFzVgVzyAMo04O67jqRlU7uuNCRB6O8Kc%3D&reserved=0)
- E-cigarettes can contain other harmful substances besides nicotine.
- Young people who use e-cigarettes may be more likely to smoke cigarettes in the future.
- Adults who do not currently use tobacco products should not start using e-cigarettes.
- If you do use e-cigarette products, you should not buy these products off the street (for example, e-cigarette or vaping products with THC or other cannabinoids).
- You should not modify e-cigarette products or add any substances to these products that are not intended by the manufacturer.
- Adult smokers who are attempting to quit should use evidence-based treatments, including counseling and FDA-approved medications. If you need help quitting tobacco products, including e-cigarettes, contact your doctor or other medical provider.

To date, no e-cigarette has been approved as a cessation device or authorized to make a modified risk claim, and more research is needed to understand the potential risks and benefits these products may offer adults who use tobacco products.

COVID-19, Smoking and Vaping: Although scientific evidence is incomplete, some studies have suggested that use of e-cigarettes may add to your risk of getting COVID -19 and may contribute to the severity of illness if you do get the virus. Smokers and e-cigarette users have to take their face masks off when they smoke or vape. So even between puffs, you may be unknowingly infected with the coronavirus, you might exhale contagious droplets and aerosols into the air, which could be inhaled by others nearby. Secondhand cigarette smoke is known to cause health problems, and although there isn’t yet scientific proof that it can spread the coronavirus and cause COVID-19, it may be possible. Smoking or vaping inside is even riskier. In a closed environment, infectious droplets and particles can build up in the air, putting others in the room at risk if there’s no ventilation.”

**How will I know about new risks or important information about the study?**

We will tell you if we learn any new information that could change your mind about taking part in this study. There will be no clinically relevant research results to disclose.

**How can the study possibly benefit me?**

This study may benefit you by advancing your knowledge of the factors that influence nicotine delivery from e-cigarettes.

**How can the study possibly benefit other people?**

The benefits to science and other people may include a better understanding of the factors that influence nicotine delivery from e-cigarettes.

**Are there any costs to participation?**

You will not have to pay for taking part in this study. The only costs include transportation and your time coming to the study visits. The lab will cover parking costs associated with the projected number of hours necessary to complete the study.

**Will I be paid for participation?**

You will be paid for taking part in this study. You will receive $800 for completing the two sessions. Payment will be provided upon completion of the 2^nd^ session. In the event of partial completion of the study procedures due to technical problems or significant discomfort (i.e. from arterial line placement or e-cigarette abstinence), participants will be compensated up to $15/hour of participation per PI discretion. If interested, participants may choose to receive partial payment of up to $15/hour of participation for the first study visit at the conclusion of the day. The remaining sum would be paid at the conclusion of the second study visit day.

What are my choices if I decide not to take part in this study?

You are free to choose whether to take part in this study or not. If you choose not to take part in this study but are interested in exploring other studies, we would be happy to refer you as appropriate. If you decide you no longer want to use nicotine products, we are happy to connect you with the appropriate treatment resources for support.

**How will you keep my data safe and private?**

We will keep information we collect about you confidential. We will share it with others if you agree to it or when we have to do it because U.S. or State law requires it. For example, we will tell somebody if you we learn that you are hurting a child or an older person. There is the possibility that the Food and Drug Administration may inspect the records.

When we publish the results of the research or talk about it in conferences, we will not use your name. If we want to use your name, we would ask you for your permission.

We will also share information about you with other researchers for future research but we will not use your name or other identifiers. We will not ask you for any additional permission. Identifiers might be removed from the identifiable private information or identifiable biospecimens and that, after such removal, the information or biospecimens could be used for future research studies or distributed to another investigator for future research studies without additional informed consent from you or your legally authorized representative

This research is covered by a Certificate of Confidentiality from the National Institutes of Health. The researchers with this Certificate may not disclose or use information, documents, or biospecimens that may identify you in any federal, state, or local civil, criminal, administrative, legislative, or other action, suit, or proceeding, or be used as evidence, for example, if there is a court subpoena, unless you have consented for this use. Information, documents, or biospecimens protected by this Certificate cannot be disclosed to anyone else who is not connected with the research except, if there is a federal, state, or local law that requires disclosure (such as to report child abuse or communicable diseases but not for federal, state, or local civil, criminal, administrative, legislative, or other proceedings, see below); if you have consented to the disclosure, including for your medical treatment; or if it is used for other scientific research, as allowed by federal regulations protecting research subjects.

**What Information Will You Collect About Me in this Study?**

The information we are asking to use and share is called “Protected Health Information.” It is protected by a federal law called the Privacy Rule of the Health Insurance Portability and Accountability Act (HIPAA). In general, we cannot use or share your health information for research without your permission. If you want, we can give you more information about the Privacy Rule. Also, if you have any questions about the Privacy Rule and your rights, you can speak to Yale Privacy Officer at 203-432-5919.

The specific information about you and your health that we will collect, use, and share includes:

- Research study records
- Medical and laboratory records of only those services provided in connection with this Study.
- Records about phone calls made as part of this research
- Records about your study visits

**How will you use and share my information?**

We will use your information to conduct the study described in this consent form.

We may share your information with:

- The U.S. Department of Health and Human Services (DHHS) agencies
- Representatives from Yale University, the Yale Human Research Protection Program and the Institutional Review Board (the committee that reviews, approves, and monitors research on human participants), who are responsible for ensuring research compliance. These individuals are required to keep all information confidential.
- The U.S. Food and Drug Administration (FDA) This is done so that the FDA can review information about the e-cigarette products involved in this research. The information may also be used to meet the reporting requirements of drug regulatory agencies.
- The study sponsor or manufacturer of study drug/device
- Health care providers who provide services to you in connection with this study.
- Laboratories and other individuals and organizations that analyze your health information in connection with this study, according to the study plan.
- Principal Investigator of the study
- Co-Investigators and other investigators
- Collaborators at American University of Beirut
- Study Coordinator and Members of the Research Team
- Data and Safety Monitoring Boards and others authorized to monitor the conduct of the Study

We will do our best to make sure your information stays private. But, if we share information with people who do not have to follow the Privacy Rule, your information will no longer be protected by the Privacy Rule. Let us know if you have questions about this. However, to better protect your health information, agreements are in place with these individuals and/or companies that require that they keep your information confidential.

**Why must I sign this document?**

By signing this form, you will allow researchers to use and disclose your information described above for this research study. This is to ensure that the information related to this research is available to all parties who may need it for research purposes. You always have the right to review and copy your health information in your medical record.

**What if I change my mind?**

The authorization to use and disclose your health information collected during your participation in this study will never expire. However, you may withdraw or take away your permission at any time. You may withdraw your permission by telling the study staff or by writing to Stephen Baldassarri, Yale School of Medicine, 300 Cedar St, TAC 455-S, New Haven, CT 06520.

If you withdraw your permission, you will not be able to stay in this study but the care you get from your doctor outside this study will not change. No new health information identifying you will be gathered after the date you withdraw. Information that has already been collected may still be used and given to others until the end of the research study to ensure the integrity of the study and/or study oversight.

**Who will pay for treatment if I am injured or become ill due to participation in the study?**

If you are injured while on study, seek treatment and contact the study doctor as soon as you are able. Yale School of Medicine and Yale-New Haven Hospital do not provide funds for the treatment of research-related injury. If you are injured as a result of your participation in this study, treatment will be provided. You or your insurance carrier will be expected to pay the costs of this treatment. No additional financial compensation for injury or lost wages is available. You do not give up any of your legal rights by signing this form.”

**What if I want to refuse or end participation before the study is over?**

Taking part in this study is your choice. You can choose to take part, or you can choose not to take part in this study. You also can change your mind at any time. Whatever choice you make, you will not lose access to your medical care or give up any legal rights or benefits.

We would still treat you with standard therapy or, at your request, refer you to a clinic or doctor who can offer this treatment. Not participating or withdrawing later will not harm your relationship with your own doctors or with this institution.

To withdraw from the study, you can call a member of the research team at any time and tell them that you no longer want to take part. There are no consequences for deciding to withdraw from the study.

The researchers may withdraw you from participating in the research if necessary. Circumstances in which this may occur are if you have difficulty tolerating or performing any of the study procedures or if you are verbally or physically abusive to study personnel.

**What will happen with my data if I stop participating?**

If you stop participating, you may request for your data to be destroyed. If you allow us to keep your data, we will continue protecting the privacy of your data and will include it in our analysis. If you allow us to keep your data, blood samples already collected will be analyzed

**Who should I contact if I have questions?**

Please feel free to ask about anything you don't understand.

If you have questions later or if you have a research-related problem, you can call the Principal Investigator, Stephen Baldassarri, MD, MHS, at (203) 824-9215.

If you have questions about your rights as a research participant, or you have complaints about this research, you call the Yale Institutional Review Boards at (203) 785-4688 or email [hrpp@yale.edu](mailto:hrpp@yale.edu).

A description of this clinical trial will be available on [*http://www.ClinicalTrials.gov*](http://www.ClinicalTrials.gov)*,* as required by U.S. Law. This Web site will not include information that can identify you. At most, the Web site will include a summary of the results. You can search this Web site at any time.

**Authorization and Permission**

Your signature below indicates that you have read this consent document and that you agree to be in this study.

We will give you a copy of this form.

| Participant Printed Name |  | Participant Signature |  | Date |
| --- | --- | --- | --- | --- |
| Person Obtaining Consent Printed Name |  | Person Obtaining Consent Signature |  | Date |
