# Supplementary material for: Nicotine flux as a powerful tool for regulating nicotine delivery from e-cigarettes: Protocol of two complimentary randomized crossover clinical trials
Source: PLoS One. 2023 Sep 21;18(9):e0291786. doi: 10.1371/journal.pone.0291786 (PMC10513228; doi:10.1371/journal.pone.0291786)
Supplement: S2 Appendix — (DOCX) [file pone.0291786.s003.docx]

Nicotine flux, a potentially powerful tool for regulating nicotine delivery from electronic cigarettes

Protocol Number

2000031973

National Clinical Trial (NCT)

NCT05706701

Principal Investigator

Stephen Baldassarri

Sponsor

NIDA

Funded by

National Institute on Drug Abuse

Protocol Version

8 March 2023

Confidentiality Statement:

Study Personnel

| **Principal Investigator** | Stephen Baldassarri  Email: stephen.baldassarri@yale.edu |
| --- | --- |

# Synopsis

| Study Description Electronic nicotine delivery systems (ENDS) heat and vaporize a nicotine-containing liquid to produce an aerosol that can deliver nicotine to the blood and the brain. ENDS are considered tobacco products in the US and are under the regulatory authority of the Food and Drug Administration. ENDS use has increase rapidly in the last decade, especially among youth: over 20% of US high school students are current ENDS users, and there is evidence of nicotine dependence in this population. Federal legislation has been proposed that would restrict ENDS liquid nicotine concentration to make ENDS "significantly less addictive and appealing to youth." However, these and other efforts to curb addiction by limiting nicotine liquid concentration are unlikely to succeed because nicotine emissions from ENDS depend on multiple variables. For example, where nicotine concentration is limited, users can turn to a higher power ENDS device to attain more nicotine. To achieve the intended public health aims, regulations targeting addiction must focus on nicotine delivery, not nicotine concentration. While nicotine delivery cannot be regulated directly, the rate at which an ENDS emits nicotine, the "nicotine flux" can be regulated and, importantly, predicted from first-principles based on knowledge of a few device design and operating variables. However, to date there is no empirical evidence demonstrating the relationship between flux and delivery, nor between flux and the physiological and subjective effects that support nicotine dependence. Closing this gap is essential for providing an effective, first-principles framework for regulating ENDS. In Aim1, we will examine the relationship between nicotine flux, nicotine form, and the rate and dose of nicotine delivery. In the clinical lab at Yale University, participants will puff on ENDS devices under conditions that differ by flux and form, while arterial blood is sampled in high time resolution. The outcome will indicate the degree to which nicotine flux and form determine the speed and dose of ENDS nicotine delivery, and thus, abuse liability. In Aim2, we will assess the relationship between nicotine flux, form, and subjective effects. At the American University of Beirut, participants will use ENDS devices with varying nicotine fluxes and forms. Dependency measures, such as urge to smoke, craving, and abstinence, will be assessed. The outcome will indicate the degree to which nicotine flux/form influence subjective effects related to dependency, puffing intensity, and exposure to toxicants. In summary, this project will provide the empirical evidence needed for public health agencies to use nicotine flux as an encompassing and convenient construct to regulate nicotine delivery from ENDS. |
| --- |
| Objectives Study Aim: Examine the influence of nicotine flux and nicotine form on the rate and dose of nicotine delivery to arterial blood. At the Yale School of Medicine, we will measure arterial nicotine concentrations over discrete time-periods (baseline, 3x post-puff, 60 sec post-use) in a within-subject study with 15 participants who will undergo 2 ENDS use sessions that differ by nicotine form (protonated, freebase). In each session, participants will draw three 3-sec puffs with four randomly ordered nicotine fluxes (9, 18, 27, 35 μg/sec). We will compute the nicotine flux with our mathematical model and verify it using our puffing robot. We hypothesize that computed nicotine flux will predict the rate of nicotine rise and maximum nicotine concentration observed in arterial blood, and that protonated nicotine will result in higher delivery. |
| Endpoints Arterial blood nicotine levels at various time points following acute ENDS use. |
| Study Population ENDS users |
| Accrual Ceiling 15 subjects |
| Phase Early Phase 1 |
| Description of Sites/Facilities Enrolling Participants Human Research Unit (HRU) |
| Description of Study Intervention Aim1 study design and method. The purpose of Aim1 at YSM is to examine the influence of nicotine flux, and nicotine form, on the rate and dose of nicotine delivery obtained from arterial blood measurements. This study involves a 4 x 2 crossover experimental design of four nicotine fluxes: 9, 18, 27, 35 μg/sec, and two nicotine forms (i.e., free-base and protonated). These nicotine fluxes are within the range reported for ENDS (3.1-111µg/sec) (El Hourani, 2022). Also, in Aim1, we will isolate the effect of form on the pharmacokinetics of nicotine delivery by controlling for puffing behavior. We will hold puff topography constant by controlling puff duration, inter-puff interval, and number of puffs using the LabVape PTL and confirm the data using eTop.  The flux/form conditions will be tested by participants in two lab visits separated by one to four weeks to minimize carryover effects. All sessions will be double-blinded. In the first visit, participants will use the ENDS device with one nicotine form and four fluxes in random order. Participants will be instructed to attend the lab for a second visit, to test the four fluxes but with the other nicotine form. The second visit will allow us to isolate the effect of nicotine form on nicotine delivery. The order of nicotine form in the two visits will be counter-balanced across participants. Outcome measures include arterial blood nicotine delivery, and puff topography. Below we provide the methodological details on device/liquid selection, preparation, flux validation, clinical lab procedures, and data analysis. Participants will be instructed to use the **Subox mini C ENDS device connected to the LabVape PTL (which limits puff duration to 3 seconds)** in four bouts separated by a 60min resting period. The e-liquids vaped are as follows: Propylene glycol(PG)/Glycerol (VG), 30/70 ratio by volume. Nicotine at different concentrations: (2, 4, 7 and 10mg/mL) Benzoic acid: ~1:1 molar ratio with nicotine. All these ingredients will be used to prepare the needed e-liquids to conduct the study. For more details, please review the ITP application.  This resting period was determined based on our previous arterial measurements on ENDS; it is deemed sufficient for the blood nicotine to go back to baseline.1 All bouts will be directed; each bout will consist of 3 puffs in which puff duration is fixed to 3sec, as in 50, and inter-puff interval fixed to 30sec (CORESTA recommended method Nº 81).123 The puff duration and inter-puff interval will be fixed using the **LabVape** PTL. A puff topography device (eTop) will record the puffing topography to identify any deviation between directed and actual puffs drawn and to measure the puffing flow rate. Participants will be trained to follow the puffing cues prior to sampling using an unpowered ENDS device. For arterial blood sampling, a radial arterial line will be placed on the non-dominant side to provide access to blood samples during vaping sessions. Each blood sample (0.5cc) will be drawn manually by a trained nurse at every time point and stored in the freezer at -25°C. Blood nicotine samples will be assayed using LCMS/ MS with deuterated internal standards, as in 114. Blood will be sampled 30sec prior to the initial puff, 5, 15, and 25sec after each puff, and 60sec after the last puff of a bout. The obtained data will be used to calculate pharmacokinetic parameters of nicotine delivery under each condition (Cmax, Tmax, dCi/dt, and AUC). AUC from 0 to 160min for the four 3-puff directed bouts will be estimated using a non-compartmental model and trapezoidal rule. All measures will be corrected for baseline values by subtracting the blood nicotine concentrations by the initial value (at the start of each bout). |
| Study Duration 3 years |
| Participant Duration 2 days |
| End of Study Definition Enrollment of all study subjects and completion of all study procedures. |

Table of Contents

[Synopsis](#_Toc2)

[Study Description](#_Toc3)

[Objectives](#_Toc4)

[Endpoints](#_Toc5)

[Study Population](#_Toc6)

[Accrual Ceiling](#_Toc7)

[Phase](#_Toc8)

[Description of Sites/Facilities Enrolling Participants](#_Toc9)

[Description of Study Intervention](#_Toc10)

[Study Duration](#_Toc11)

[Participant Duration](#_Toc12)

[Schema](#_Toc13)

[Schedule of Activities (SoA)](#_Toc14)

[End of Study Definition](#_Toc15)

[1 - Statement of Compliance](#_Toc16)

[1.1 Statement of Compliance](#_Toc17)

[2 - Introduction](#_Toc18)

[2.1 Study Rationale](#_Toc19)

[2.2 Background](#_Toc20)

[2.3 Risk/Benefit Assessment](#_Toc21)

[2.3.1 Known Potential Risks](#_Toc22)

[2.3.2 Known Potential Benefits](#_Toc23)

[2.3.3 Assessment of Potential Risks and Benefits](#_Toc24)

[3 - Objectives and Endpoints](#_Toc25)

[3.1 Objectives and Endpoints](#_Toc26)

[4 - Study Design](#_Toc27)

[4.1 Overall Design](#_Toc28)

[4.2 Scientific Rationale for Study Design](#_Toc29)

[4.3 Justification for Dose](#_Toc30)

[4.4 End of Study Definition](#_Toc31)

[5 - Study Population](#_Toc32)

[5.1 Inclusion Criteria](#_Toc33)

[5.2 Exclusion Criteria](#_Toc34)

[5.3 Lifestyle Considerations](#_Toc35)

[5.4 Screen Failures](#_Toc36)

[5.5 Strategies for Recruitment and Retention](#_Toc37)

[6 - Study Intervention](#_Toc38)

[6.1.1 Study Intervention Description](#_Toc39)

[6.1.2 Dosing and Administration](#_Toc40)

[6.2 Preparation/Handling/Storage/Accountability](#_Toc41)

[6.2.1 Acquisition and accountability](#_Toc42)

[6.2.2 Formulation, Appearance, Packaging, and Labeling](#_Toc43)

[6.2.3 Product Storage and Stability](#_Toc44)

[6.2.4 Preparation](#_Toc45)

[6.3 Measures to Minimize Bias: Randomization and Blinding](#_Toc46)

[6.4 Study Intervention Compliance](#_Toc47)

[6.5 Concomitant Therapy](#_Toc48)

[6.5.1 Rescue Medicine](#_Toc49)

[7 - Study Intervention Discontinuation and Participant Discontinuation/Withdrawal](#_Toc50)

[7.1 Discontinuation of Study Intervention](#_Toc51)

[7.2 Participant Discontinuation/Withdrawal from the Study](#_Toc52)

[7.3 Lost to Follow-Up](#_Toc53)

[8 - Study Assessments and Procedures](#_Toc54)

[8.1 Efficacy Assessments](#_Toc55)

[8.2 Safety and Other Assessments](#_Toc56)

[8.3 Adverse Events and Serious Adverse Events](#_Toc57)

[8.3.1 Definition of Adverse Events (AE)](#_Toc58)

[8.3.2 Definition of Serious Adverse Events (SAE)](#_Toc59)

[8.3.3 Classification of an Adverse Event](#_Toc60)

[8.3.3.1 Severity of Event](#_Toc61)

[8.3.3.2 Relationship to Study Intervention](#_Toc62)

[8.3.3.3 Expectedness](#_Toc63)

[8.3.4 Time Period and Frequency for Event Assessment and Follow-Up](#_Toc64)

[8.3.5 Adverse Event Reporting](#_Toc65)

[8.3.6 Serious Adverse Event Reporting](#_Toc66)

[8.3.7 Reporting Events to Participants](#_Toc67)

[8.3.8 Events of Special Interest](#_Toc68)

[8.3.9 Reporting of Pregnancy (if applicable)](#_Toc69)

[8.4 Unanticipated Problems](#_Toc70)

[8.4.1 Definition of Unanticipated Problems (UP)](#_Toc71)

[8.4.2 Unanticipated Problem Reporting](#_Toc72)

[8.4.3 Reporting Unanticipated Problems to Participants](#_Toc73)

[9 - Statistical Considerations](#_Toc74)

[9.1 Statistical Hypotheses](#_Toc75)

[9.2 Sample Size Considerations](#_Toc76)

[9.3 Populations for Analyses](#_Toc77)

[9.4 Statistical Analyses](#_Toc78)

[9.4.1 General Approach](#_Toc79)

[9.4.2 Analysis of the Primary Efficacy Endpoint(s)](#_Toc80)

[9.4.3 Analysis of the Secondary Endpoint(s)](#_Toc81)

[9.4.4 Safety Analyses](#_Toc82)

[9.4.5 Baseline Descriptive Analyses (if applicable)](#_Toc83)

[9.4.6 Planned Interim Analyses (if applicable)](#_Toc84)

[9.4.7 Sub-Group Analyses](#_Toc85)

[9.4.8 Tabulation of Individual Participant Data](#_Toc86)

[9.4.9 Exploratory Analyses](#_Toc87)

[10 - Supporting Documentation and Operational Considerations](#_Toc88)

[10.1 Regulatory, Ethical, and Study Oversight Considerations](#_Toc89)

[10.1.1 Informed Consent Process](#_Toc90)

[10.1.1.1 Consent/Assent and Other Informational Documents Provided to Participants](#_Toc91)

[10.1.1.2 Consent Procedures and Documentation](#_Toc92)

[10.1.2 Study Discontinuation and Closure](#_Toc93)

[10.1.3 Data Confidentiality and Participant Policy](#_Toc94)

[10.1.4 Future Use of Stored Human Specimens and Data](#_Toc95)

[10.1.5 Safety Oversight](#_Toc96)

[10.1.6 Key Roles and Study Governance](#_Toc97)

[10.1.7 Clinical Monitoring](#_Toc98)

[10.1.8 Quality Assurance and Quality Control](#_Toc99)

[10.1.9 Data Handling and Record Keeping](#_Toc100)

[10.1.9.1 Data Collection and Management Responsibilities](#_Toc101)

[10.1.9.2 Study Records Retention](#_Toc102)

[10.1.10 Protocol Deviations](#_Toc103)

[10.1.11 Publication and Data Sharing Policy](#_Toc104)

[10.1.12 Conflict of Interest Policy](#_Toc105)

[10.2 Additional Considerations](#_Toc106)

[10.3 Protocol Amendment History](#_Toc107)

[10.4 Abbreviations](#_Toc108)

# 1 - Statement of Compliance

## 1.1 Statement of Compliance

The trial will be carried out in accordance with International Conference on Harmonization Good Clinical Practice (ICH GCP) and the following:

- - United States (US) Code of Federal Regulations (CFR) applicable to clinical studies (45 CFR Part 46, 21 CFR Part 50, 21 CFR Part 56, 21 CFR Part 312, and/or 21 CFR Part 812)

National Institutes of Health (NIH)-funded investigators and clinical trial site staff who are responsible for the conduct, management, or oversight of NIH-funded clinical trials have completed Human Subjects Protection and ICH GCP Training.

The protocol, informed consent form(s), recruitment materials, and all participant materials will be submitted to the Institutional Review Board (IRB) for review and approval. Approval of both the protocol and the consent form must be obtained before any participant is enrolled. Any amendment to the protocol will require review and approval by the IRB before the changes are implemented to the study. In addition, all changes to the consent form will be IRB-approved; a determination will be made regarding whether a new consent needs to be obtained from participants who provided consent, using a previously approved consent form.

# 2 - Introduction

## 2.1 Study Rationale

Nicotine flux, the rate of nicotine emitted from ENDS, encapsulates several use-factors that impact nicotine emissions including power and liquid composition. Importantly, nicotine flux may serve as a measure of the rate of nicotine delivery, a key determinant of nicotine dependence. Nicotine flux therefore presents a potentially powerful regulatory lever, if it can be shown empirically to predict nicotine delivery and subjective effects relevant to dependence.

## 2.2 Background

Electronic nicotine delivery systems (ENDS) heat and vaporize a nicotine-containing liquid to produce an aerosol that when inhaled can deliver nicotine to the bloodstream and the brain.1 ENDS are considered tobacco products in the US and are under the regulatory authority of the Food and Drug Administration (FDA).2 ENDS use has increased rapidly in the last decade, especially among youth: over 20% of US high school students are current ENDS users, and there is evidence of nicotine dependence in this population.3 In 2019 federal legislation was proposed that would restrict ENDS liquid nicotine concentration to make ENDS "significantly less addictive and appealing to youth."4 However, these and other efforts to curb addiction by limiting nicotine liquid concentration are unlikely to succeed because nicotine emissions from ENDS depend on multiple variables in combination, including device power and design, liquid composition, and user puffing behavior.5 For example, where nicotine concentration is limited, users can readily circumvent the intent of the limit by resorting to a higher power ENDS device.6 Therefore to achieve the intended effect, regulations targeting addiction must focus on nicotine delivery, not nicotine concentration. While nicotine delivery cannot be regulated directly, the rate at which an ENDS emits nicotine, the "nicotine flux" (reported as micro grams/second), can be characterized and regulated.7,8 Apart from providing an encompassing performance metric for characterizing ENDS emissions, flux represents the potential speed at which nicotine can reach the brain, a key factor for reinforcement and addiction.9-11 We have previously demonstrated that flux can be accurately and precisely predicted by computer simulation from knowledge of a few device design and operating variables, even before a product has left the drawing board.12 But while nicotine flux provides a theoretically intuitive regulatory approach, there is no empirical evidence demonstrating a relationship between flux and the speed and dose of nicotine delivered to an ENDS user, nor to the subjective effects associated with addiction. Closing these knowledge gaps is crucial for deploying flux in nicotine regulation. Another factor that influences nicotine delivery is nicotine form (i.e. protonated/freebase ratio).13,14 The nicotine ratios of ENDS liquids vary widely,15 and influence sensory experience and pharmacokinetics.16,17 Importantly, form is readily incorporated in a nicotine flux framework, i.e., protonated and freebase nicotine flux. The speed of nicotine delivery to the brain can be assessed accurately by monitoring arterial blood nicotine concentration while a participant puffs a tobacco product. Unlike venous blood concentration, arterial blood provides a high-fidelity picture of the pharmacokinetics of delivery that is not masked by metabolic factors. Measuring time-resolved arterial blood nicotine concentration while participants use ENDS provides an accurate means to validate nicotine flux as a predictor of the rate and dose of nicotine delivery to the brain. Furthermore, subjective effects of nicotine can be readily measured using validated instruments pre- and postadministration.

Therefore, our specific aims are to: 1. Examine the influence of nicotine flux and nicotine form on the rate and dose of nicotine delivery to arterial blood. At the Yale School of Medicine, we will measure arterial nicotine concentrations over discrete time-periods (baseline, 3xpost-puff, 60sec post-use) in a within-subject study with 15 participants who will undergo 2 ENDS use sessions that differ by nicotine form (protonated, freebase). In each session, participants will draw three 3-sec puffs with four randomly ordered nicotine fluxes (9, 18, 27, 35 μg/sec). The puff duration will be controlled using the LabVape PTL. We will compute the nicotine flux with our mathematical model and verify it using our puffing robot. We hypothesize that computed nicotine flux will predict the rate of nicotine rise and maximum nicotine concentration observed in arterial blood, and that protonated nicotine will result in higher delivery. 2. Assess the influence of nicotine flux and nicotine form on subjective effects. At the American University of Beirut, we will assess subjective effects (e.g. product liking, nicotine craving) and puffing topography for 130 participants who will undergo 5 ENDS use sessions consisting of 2 bouts (10 puffs + 60min ad libitum) with 2 fluxes (18, 35μg/s) x 2 forms (protonated, freebase) and a 0 nicotine condition. In addition, we will use a state-of-the-art device (NIDA 1R01DA025659) to sample in situ a fraction of the aerosol generated during each puff to verify delivered nicotine flux and form, and measure exposure to pulmonary toxicants (carbonyl species). We hypothesize that increasing nicotine flux and protonated nicotine will result in greater reductions of nicotine craving, and lower puffing intensity and pulmonary toxicant exposure.

Summary. Regulating ENDS products to minimize addiction is a public health priority. A promising approach is one that limits the rate at which a device delivers nicotine. Using trans-disciplinary analytical and clinical lab methods these studies will link flux, a readily regulated product performance parameter, to acute physiological and subjective effects associated with addiction in ENDS users. These studies will provide regulators a powerful, empirically tested product performance-based parameter to limit the risk profile of ENDS products.

## 2.3 Risk/Benefit Assessment

### 2.3.1 Known Potential Risks

***Risks Associated with Use of an Arterial Line.***

There are small risks associated with the placement of an arterial catheter. Certain individuals may feel light-headed during arterial catheter placement. Arterial catheter placement may be associated with mild-to-moderate pain, hematoma, inflammation, bleeding, or bruising at the puncture site. If any of these, or other, symptoms occur and do not diminish within 24 to 72 hours after the arterial line removal, or in the event that they worsen, subjects will be advised to seek immediate medical attention (i.e., call your primary care physician’s office and/or, go to a local Hospital or Urgent Care Center for an immediate evaluation. In rare instances blocking of the artery, tearing of the artery, arterial leakage, poor healing, nerve damage or infection at the catheter insertion site, may occur.

Risks of radial artery cannulation are minimized by having the procedure performed by an experienced health care provider. The health care provider would be a physician or an Advanced Practice Provider (APP): for example, a physician assistant (PA) or an advanced practice registered nurse (APRN) with experience in placement of arterial catheters.

Pain is minimized by local anesthesia. Bleeding is prevented by local pressure applied for a minimum of 15 minutes after catheter removal. Subjects will have their hand and finger blood supply examined after arterial cannulation and again following catheter removal. Also, subjects will be asked to abstain from anticoagulants for 7-10 days prior to arterial line insertion and 7-10 days following arterial line removal. Subjects will be provided a 24-hour emergency physician telephone number to call if they encounter pain, discoloration, numbness, tingling, coolness, hematoma, inflammation, or any other unusual symptoms in the wrist or hand, or fever, chills or drainage from the vascular puncture sites, following the procedure. In addition, if an emergency arises at the time of cannulation or scanning, 911 will be called, and the subject will be sent to the Emergency Department for evaluation and treatment. Nurses will provide the subjects an instruction sheet documenting problems to watch for and procedures to follow should such problems occur. Infection is avoided by adequate cleansing of the skin prior to intravascular line insertion.

***Risks Associated with Blood Drawing***

Drawing blood and inserting an intravenous line (IV) into an arm vein are safe and standard medical procedures. Sometimes a bruise will occur at the puncture site and rarely a blood clot or infection will occur in the vein. You should not donate blood for at least 8 weeks after the study. The total volume of blood collected during this study will be up to 44-88 cc. This amount of blood is safe for healthy persons.

***Risks Associated with E-Cigarette Use***

Over various Committee reviews of e-cigarette studies during EVALI and COVID-19, the IRB along with the PI’s, has developed standard criteria for risk considerations in these studies where the lungs may be uniquely affected.

- Risk of the device overheating, fire, or explosion to the device. This is extremely rare. Staff are educated on proper device inspection, operation, charging/recharging, storage, and final disposition.
- We will assess the participant’s health at the intake to make sure she/he is healthy prior to participating and will continue to monitor their health closely during the study. If they experience any symptoms (such as abdominal pain, nausea, vomiting, diarrhea, cough, shortness of breath, chest pain) or other concerns, they will be advised to let their doctor know promptly (right away).   They will go to the emergency room if their symptoms increase.  They can stop the study at any point. If they feel any discomfort or need to stop for any reason, they will be instructed to let the research team know.
- E-cigarette liquid contain others chemicals besides nicotine including propylene glycol/vegetable glycol/vegetable glycerin. At this time, we do not know the risks associated with the propylene glycol/ vegetable glycerin that may in the fillers in the liquids used in this study.  Benzoic acid is also present and is part of commercially available e-liquids.
- It is important to note that there may be unforeseen risks (such as allergic reactions). Some research has indicated that in large doses propylene glycol and vegetable glycerin can be harmful. However, if they experience any side effects, they can stop the session at any point. Research staff will monitor e-cigarette use during the lab session. If they feel any discomfort or need to stop for any reason, they will be advised to let the researcher know.

***The CDC has warned against vaping e-cigarettes***

There have been recent reported cases of severe lung (pulmonary) illness linked to “vaping” or e-cigarette use. These cases included symptoms such as coughing, shortness of breath, chest pain, fever, fatigue, nausea, vomiting, diarrhea, and/or abdominal pain. Some patients reported symptoms to have occurred over a few days and some reported to have occurred over a few weeks. Vaping-related disorders have ranged from mild to severe with hospitalization, intensive care with breathing machines and in some cases death.  In most cases, but not all, people experiencing these symptoms were using cannabidiol (CBD) or marijuana (THC) e-liquids, and/or were using e-cigarette devices and e-liquids that were mixed at home or purchased off market (such as purchasing an e-liquid or device on the street, not from a licensed retailer).

The Center for Disease Control ([www.cdc.gov](https://nam12.safelinks.protection.outlook.com/?url=http%3A%2F%2Fwww.cdc.gov%2F&data=04%7C01%7Cstephen.baldassarri%40yale.edu%7C7a683a4d33304fcce38a08d9f01ab290%7Cdd8cbebb21394df8b4114e3e87abeb5c%7C0%7C0%7C637804820067002900%7CUnknown%7CTWFpbGZsb3d8eyJWIjoiMC4wLjAwMDAiLCJQIjoiV2luMzIiLCJBTiI6Ik1haWwiLCJXVCI6Mn0%3D%7C3000&sdata=tDZsPSXjrZY52JC2AJYmMaJkBLb3BmjTn8BC8JKUuLA%3D&reserved=0)) has issued the following information on vaping:

·         The use of e-cigarettes is unsafe for kids, teens, and young adults.

·         Most e-cigarettes contain nicotine. Nicotine is highly addictive and can harm adolescent brain development, which continues into the early to mid-20s.[^*^](https://nam12.safelinks.protection.outlook.com/?url=https%3A%2F%2Fwww.cdc.gov%2Ftobacco%2Fbasic_information%2Fe-cigarettes%2FQuick-Facts-on-the-Risks-of-E-cigarettes-for-Kids-Teens-and-Young-Adults.html%23one&data=04%7C01%7Cstephen.baldassarri%40yale.edu%7C7a683a4d33304fcce38a08d9f01ab290%7Cdd8cbebb21394df8b4114e3e87abeb5c%7C0%7C0%7C637804820067002900%7CUnknown%7CTWFpbGZsb3d8eyJWIjoiMC4wLjAwMDAiLCJQIjoiV2luMzIiLCJBTiI6Ik1haWwiLCJXVCI6Mn0%3D%7C3000&sdata=rC%2Bz9szwbofFzVgVzyAMo04O67jqRlU7uuNCRB6O8Kc%3D&reserved=0)

·         E-cigarettes can contain other harmful substances besides nicotine.

·         Young people who use e-cigarettes may be more likely to smoke cigarettes in the future.

·         Adults who do not currently use tobacco products should not start using e-cigarettes.

·         If you do use e-cigarette products, you should not buy these products off the street (for example, e-cigarette or vaping products with THC or other cannabinoids).

·         You should not modify e-cigarette products or add any substances to these products that are not intended by the manufacturer.

·         Adult smokers who are attempting to quit should use evidence-based treatments, including counseling and FDA-approved medications. If you need help quitting tobacco products, including e-cigarettes, contact your doctor or other medical provider.

COVID-19, Smoking and Vaping: Although scientific evidence is incomplete, some studies have suggested that use of e-cigarettes may add to your risk of getting COVID -19 and may contribute to the severity of illness if you do get the virus. Smokers and e-cigarette users have to take their face masks off when they smoke or vape. So even between puffs, you may be unknowingly infected with the coronavirus, you might exhale contagious droplets and aerosols into the air, which could be inhaled by others nearby. Secondhand cigarette smoke is known to cause health problems, and although there isn’t yet scientific proof that it can spread the coronavirus and cause COVID-19, it may be possible. Smoking or vaping inside is even riskier. In a closed environment, infectious droplets and particles can build up in the air, putting others in the room at risk if there’s no ventilation.

Risk minimizing procedures:

- - *In this study we monitor for the symptoms of EVALI, which is a direct benefit for those already smoking/vaping and may be at risk for developing this condition.*
  - *As with all participants- Participants who report recent THC vaping (within the last 90 days) will be monitored for EVALI / COVID-19 symptoms, at screening and the Health Assessment checklist at screening and at each visit.*
  - *If a participant indicates EVALI symptoms (i.e. cough, shortness of breath, chest pain, nausea, vomiting, stomach pain, diarrhea, fever, chills, or weight loss), the researcher will assess if the symptom is related and proximal to a non-EVALI / COVID- cause (e.g. Nausea/vomiting related to food poisoning, Shortness of breath related to seasonal allergies). If a reasonable & proximal cause is identified, participant will continue with*

*session and ongoing monitoring. Otherwise, the participant will be deemed ineligible (if identified at intake) or withdrawn (at lab sessions).*

- - *We will consult with study nurse and/or study pulmonologist for any cases in which the reasonable/proximal cause is not apparent or unclear.*
  - Provide participants who report vaping THC will be provided education material about EVALI from <https://www.yalemedicine.org/conditions/evali>
  - *All participants will be offered smoking cessation materials at the end of participation.*

### 2.3.2 Known Potential Benefits

This study is of no direct benefit to participants who are currently smoking All participants in this study may derive subjective benefit from volunteering to take part in a study for the advancement of scientific knowledge. They will have direct consultation with experts in tobacco product use in the study team.

### 2.3.3 Assessment of Potential Risks and Benefits

There is a balance between potential risks and benefits.

# 3 - Objectives and Endpoints

## 3.1 Objectives and Endpoints

The study objective is to measure arterial nicotine concentrations over discrete time-periods (baseline, 3xpost-puff, 60sec post-use) in a within-subject study with 15 participants who will undergo 2 ENDS use sessions that differ by nicotine form (protonated, freebase). In each session, participants will draw three 3-sec puffs with four randomly ordered nicotine fluxes (9, 18, 27, 35 μg/sec). We will compute the nicotine flux with our mathematical model and verify it using our puffing robot. We hypothesize that computed nicotine flux will predict the rate of nicotine rise and maximum nicotine concentration observed in arterial blood, and that protonated nicotine will result in higher delivery.

# 4 - Study Design

## 4.1 Overall Design

Aim1 study design and method. The purpose of Aim1 at YSM is to examine the influence of nicotine flux, and nicotine form, on the rate and dose of nicotine delivery obtained from arterial blood measurements. This study involves a 4 x 2 crossover experimental design of four nicotine fluxes: 9, 18, 27, 35 μg/sec and two nicotine forms (i.e., free-base and protonated). These nicotine fluxes are within the range reported for ENDS (3.1-111µg/sec) (El Hourani, 2022). Also, in Aim1, we will isolate the effect of form on the pharmacokinetics of nicotine delivery by controlling for puffing behavior. We will hold puff topography constant by controlling puff duration, inter-puff interval, and number of puffs using the PTL technology and confirm the data using eTop.

The flux/form conditions will be tested by participants in two lab visits separated by one to four weeks to minimize carryover effects. All sessions will be double-blinded. In the first visit, participants will use the ENDS device with one nicotine form and four fluxes in random order. Participants will be instructed to attend the lab for a second visit, to test the four fluxes but with the other nicotine form. The second visit will allow us to isolate the effect of nicotine form on nicotine delivery. The order of nicotine form in the two visits will be counter-balanced across participants. Outcome measures include arterial blood nicotine delivery, and puff topography. Below we provide the methodological details on device/liquid selection, preparation, flux validation, clinical lab procedures, and data analysis. Participants will be instructed to use the Subox mini C ENDS device connected to the LabVape PTL (which limits puff duration to 3 seconds) in four bouts separated by a 60min resting period. This period was determined based on our previous arterial measurements on ENDS; it is deemed sufficient for the blood nicotine to go back to baseline.1 All bouts will be directed; each bout will consist of 3 puffs in which puff duration is fixed to 3sec, as in 50, and inter-puff interval fixed to 30sec (CORESTA recommended method Nº 81).123 The puff duration and inter-puff interval will be fixed using the PTL. A puff topography device (eTop) will record the puffing topography to identify any deviation between directed and actual puffs drawn, for data analysis purposes. Participants will be trained to follow the puffing cues prior to sampling using an unpowered ENDS device. For arterial blood sampling, a radial arterial line will be placed on the non-dominant side to provide access to blood samples during vaping sessions. Each blood sample (0.5cc) will be drawn manually by a trained nurse at every time point and stored in the freezer at -25°C. Blood nicotine samples will be assayed using LCMS/ MS with deuterated internal standards, as in 114. Blood will be sampled 30sec prior to the initial puff, 5, 15, and 25sec after each puff, and 60sec after the last puff of a bout. The obtained data will be used to calculate pharmacokinetic parameters of nicotine delivery under each condition (Cmax, Tmax, dCi/dt, and AUC). AUC from 0 to 160min for the four 3-puff directed bouts will be estimated using a non-compartmental model and trapezoidal rule. All measures will be corrected for baseline values by subtracting the blood nicotine concentrations by the initial value (at the start of each bout). Each study visit will last approximately 6 hours. If a bout fails for technical reasons, there will be a repeat of that bout, with the permission of the participant.

## 4.2 Scientific Rationale for Study Design

As described above, the study design will allow for testing of various nicotine fluxes in an efficient manner that maximizes time for the study participants and investigators.

## 4.3 Justification for Dose

The doses administered reflect commercially available products and will inform regulatory strategies.

## 4.4 End of Study Definition

Enrollment of all study subjects and completion of all study procedures.

# 5 - Study Population

## 5.1 Inclusion Criteria

All the participants must be:

- Above 21 years of age
- Use ENDS at least 3 months and at least 3 times a week.
- Be willing to provide informed consent, attend the lab, and abstain from tobacco/nicotine as required.
- Have a normal Allen test

## 5.2 Exclusion Criteria

- Any significant current medical condition such as neurological, cardiovascular, endocrine, renal, or hepatic pathology that would increase risk or would interfere with/mimic tobacco abstinence
- Untreated, unresolved active pulmonary or cardiovascular conditions (i.e. chest pain, dyspnea, acute infection, recurring bronchitis, and reactive airway disorder)
- Breast-feeding or Pregnant (by urinalysis at screening).
- Vaping less than 3 months and less than 3 times per week
- Taking anticoagulants and blood thinners
- Known hypersensitivity to propylene glycol
- History of environmental – bronchospastic allergies, multiple chemical sensitivities, or other airway sensitivities that require the use of an epi pen or that in the investigator’s view would make it risky for participation.
- Not fully vaccinated for COVID 19.
- Has current symptoms as identified by the Health Assessment Checklist including cough, shortness of breath, chest pain, nausea, vomiting, stomach pain, diarrhea, fever, chills, or weight loss)
- Participants intending to quit tobacco/nicotine use in the next 30 days will be excluded and referred to cessation treatment.
- Abnormal Allen Test (impaired collateral circulation)
- Positive pregnancy test at any study visit
- Infection of skin or soft tissue at insertion site (erythema, swelling, ulceration)
- Peripheral vascular disease
- Coronary artery disease/advance atherosclerosis
- Raynaud’s phenomenon
- Coagulopathy (hereditary bleeding disorders, advanced liver disease)
- Thromboangiitis obliterans
- COPD/emphysema/chronic bronchitis
- Allergy to lidocaine or anesthetics
- Inability to tolerate blood draws for any reason
- Additional Screening Procedures: The screening visit will take about 1 hour. Each assessment will take between 1-3 minutes to complete. Significant changes and/or abnormalities in these assessments during the study period may warrant exclusion at the discretion of the PI.
  - Participants will be asked to answer the 4-item PROMIS physical function scale and will be excluded if they report greater than “without any difficulty” on any single item as a screening tool to instead of completing an ECG – consistent with other approved studies HIC 2000023007, 2000032211.
  - Spirometry (baseline and before each visit)
  - Cotinine Urinalysis to confirm vaping
  - Physical Exam
  - Collection of smoking and vaping history
  - Application of the self-reported signs and symptoms health questionnaire with exclusion of those with chronic symptoms that would interfere with monitoring of vaping complications (baseline and * before each visit). Participants will be excluded and referred for medical treatment if they indicate dyspnea or cough symptoms are “severe.”
  - PROMIS Dyspnea Severity Item Pool
  - PROMIS Dyspnea Characteristics
  - PROMIS Fatigue Short Form
  - Functional Assessment of Chronic Illness Therapy (FACIT) Cough item

*This will be reviewed, ongoing, by PI and study physician and positive findings of a change in symptom or single severe symptom will trigger clinical referral to a treating physician or ED.

## 5.3 Lifestyle Considerations

All participants will undergo a one-hour observation period prior to each study session during which no nicotine/tobacco product will be permitted.

## 5.4 Screen Failures

Screen failures are defined as participants who consent to participate in the clinical trial but are not subsequently randomly assigned to the study intervention or entered in the study. A minimal set of screen failure information is required to ensure transparent reporting of screen failure participants, to meet the Consolidated Standards of Reporting Trials (CONSORT) publishing requirements and to respond to queries from regulatory authorities. Minimal information includes demography, screen failure details, eligibility criteria, and any serious adverse event (SAE).

Individuals who do not meet the criteria for participation in this trial (screen failure) because of changes in ENDS status may be rescreened. Rescreened participants should be assigned the same participant number as for the initial screening.

## 5.5 Strategies for Recruitment and Retention

All research subjects will be recruited under guidelines of the Yale University Institutional Review Board (Human Investigation Committee). Subjects will be recruited through a variety of advertising methods (study flyers, posters, internet/web postings, radio, newspaper, and public access TV) as well as through referrals from other research groups/clinicians at the Connecticut Mental Health Center, the Yale-New Haven Health System, the Yale Tobacco Center of Regulatory Science, and the Yale Translational Brain Imaging Program. Potential subjects will be encouraged to contact our study recruitment line for inclusion in our study. Subjects are recruited through our recruitment phone line. A member of our research staff will describe the study to participants who call, answer any questions the potential subjects have, and then complete a phone screening questionnaire to determine the subject's eligibility for an in-person screening visit. If an individual appears to meet enrollment criteria and is interested in participating, a face-to-face interview is conducted by the study staff and study physician. A release of information is obtained for review of any available historical and clinical data. A written authorization form is also obtained from each subject, permitting the research team to use, create, or disclose the subject's PHI for research purposes. The nature of the project, procedures, relative risks and benefits, and alternatives to participation in the project are discussed with the individual. Following this discussion, the individual is given a copy of the consent form to review, and any questions are answered. The process of informed consent will be obtained in accordance with local IRB standards by study personnel who have participated in institutionally approved training in human subject protection. Upon obtaining voluntary, written, informed consent, medical and psychiatric screening procedures will be used to confirm study eligibility. Subjects are free to discontinue their participation in the research at any time by requesting this verbally or in writing. Retention of subjects will be maximized by maintaining close contact with study subjects through multiple forms of communication (i.e. phone, text, e-mail) throughout the course of the study. Subjects will also be compensated for their time as both an inducement to participate and complete study procedures (by amounts / methods deemed appropriate and approved by our local IRB). Participants will receive $800 after completing the two lab sessions. In the event of partial completion of the study procedures due to technical problems or significant discomfort (i.e. from arterial line placement or e-cigarette abstinence), participants will be compensated up to $15/hour of participation per PI discretion. If interested, participants may choose to receive partial payment of up to $15/hour of participation for the first study visit at the conclusion of the day. The remaining sum would be paid at the conclusion of the second study visit day.

# 6 - Study Intervention

### 6.1.1 Study Intervention Description

In Aim1, the users will be asked to puff on an ENDS device at designated fluxes, and arterial blood samples will be collected at baseline, followed by 5, 15, and 25sec after each puff, and 60sec after the last puff of a bout. There are 30 seconds between puffs. The four nicotine fluxes: 9, 18, 27, 35 μg/sec. The users' puffing time and inter-puff interval will be controlled via the ARL Puff Time Limiter (PTL). In addition, the users' puffing behaviors will be monitored and verified using eTop. Both state-of-the-art technologies (PTL and eTop) will be described in the analytical lab work section. The ENDS liquids to be used in this study will be prepared in the analytical lab at AUB, and the nicotine flux generated from the ENDS devices will be verified using the AUB Aerosol Lab Vaping Instrument (ALVIN). We describe these instruments and methods in more detail in the analytical section below.

OUTCOME MEASURES. In the Yale clinical lab, outcome measures include maximum arterial blood level nicotine concentration (Cmax), the rate of nicotine rise after the initial puff (dCi/dt), time to maximum arterial blood level nicotine concentration (Tmax), area under the curve (AUC) for nicotine, liquid consumed (determined by ENDS gravimetric weight change, pre-post), and puffing topography records.

### 6.1.2 Dosing and Administration

In each session, participants will draw three 3-sec puffs with four randomly ordered nicotine fluxes (9, 18, 27, 35 μg/sec). Each bout of 3 puffs is separated by 1 hour to allow nicotine blood levels to return to baseline.

## 6.2 Preparation/Handling/Storage/Accountability

### 6.2.1 Acquisition and accountability

Products will be acquired through research collaborators or other vendors. The PI or other research team members will be accountable for receipt and storage of supplies.

### 6.2.2 Formulation, Appearance, Packaging, and Labeling

ENDS DEVICE, LIQUID, PREPARATION AND CONDITIONING. We will use the Subox Mini C ENDS device connected to the LabVape PTL (which limits puff duration to 3 seconds) because this device has proven to efficiently deliver nicotine to blood in our previous work. All necessary Subox Mini C devices will be shipped from the US to AUB. Liquids will be prepared from a stock of analytical grade 30/70 PG/VG liquid. This ratio is prevalent in ENDS devices, including JUUL. We predict with our mathematical model that for a Subox Mini C operating at 20W with a 30/70 PG/VG liquid, nicotine concentrations of 2, 4, 7, and 10 mg/mL will provide fluxes of 9, 18, 27, 35 μg/sec, respectively. Protonated nicotine liquids will be prepared by adding benzoic acid to the stock liquid of a given concentration. The predicted nicotine fluxes for the Subox Mini C will be verified at AUB using ALVIN.

### 6.2.3 Product Storage and Stability

All liquids will be stored in the dark at 5^0^ C in sealed containers.

### 6.2.4 Preparation

E-liquids will be prepared according to nicotine concentrations predicted by the mathematical model and verified with ALVIN at the AUB analytical lab. A stringent device conditioning protocol will be used to ensure reproducibility in all studies, as we have reported in our publications.

## 6.3 Measures to Minimize Bias: Randomization and Blinding

The flux/form conditions will be tested by participants in two lab visits separated by one to four weeks to minimize carryover effects. All sessions will be double-blinded. In the first visit, participants will use the ENDS device with one nicotine form and four fluxes in random order. Participants will be instructed to attend the lab for a second visit, to test the four fluxes but with the other nicotine form (free base or protonated). The second visit will allow us to isolate the effect of nicotine form on nicotine delivery. The order of nicotine form in the two visits will be counter-balanced across participants.

## 6.4 Study Intervention Compliance

Adherence to the protocol will be assessed by direct observation of study subjects completing the required tasks. Task completion will be recorded in REDCap.

## 6.5 Concomitant Therapy

Not applicable.

### 6.5.1 Rescue Medicine

Not applicable.

# 7 - Study Intervention Discontinuation and Participant Discontinuation/Withdrawal

## 7.1 Discontinuation of Study Intervention

The study intervention will be discontinued if:

-Any serious adverse events occur

-The study subject chooses to discontinue the intervention

-The PI or research staff chooses to discontinue the intervention

## 7.2 Participant Discontinuation/Withdrawal from the Study

Participants are free to withdraw from participation in the study at any time upon request.

An investigator may discontinue or withdraw a participant from the study for the following reasons:

- Pregnancy
- Significant study intervention non-compliance
- If any clinical adverse event (AE), laboratory abnormality, or other medical condition or situation occurs such that continued participation in the study would not be in the best interest of the participant
- Disease progression which requires discontinuation of the study intervention
- If the participant meets an exclusion criterion (either newly developed or not previously recognized) that precludes further study participation
- Participant unable to receive the study intervention> for 4 weeks.

The reason for participant discontinuation or withdrawal from the study will be recorded in the database. Subjects who sign the informed consent form and are randomized but do not receive the study intervention may be replaced. Subjects who sign the informed consent form, and are randomized and receive the study intervention, and subsequently withdraw, or are withdrawn or discontinued from the study, may be replaced.

## 7.3 Lost to Follow-Up

A participant will be considered lost to follow-up if he or she fails to return for their scheduled visits and is unable to be contacted by the study site staff.

The following actions must be taken if a participant fails to return to the clinic for a required study visit:

- The site will attempt to contact the participant and reschedule the missed visit and counsel the participant on the importance of maintaining the assigned visit schedule and ascertain if the participant wishes to and/or should continue in the study.
- Before a participant is deemed lost to follow-up, the investigator or designee will make every effort to regain contact with the participant (where possible, 3 telephone calls and, if necessary, a certified letter to the participant's last known mailing address or local equivalent methods). These contact attempts should be documented in the participant's medical record or study file.
- Should the participant continue to be unreachable, he or she will be considered to have withdrawn from the study with a primary reason of lost to follow-up.

# 8 - Study Assessments and Procedures

## 8.1 Efficacy Assessments

HISTORY/DEMOGRAPHICS. Demographic data including age, gender, race, socio-economic status, marital status, educational, and occupational levels will be assessed using a screening survey. The collected information will be used to ascertain eligibility. The PROMIS Global Health measure (10 items) will provide an assessment of a participant's assessment of his/her health (physical, emotional, and social)

OUTCOME MEASURES. In the Yale clinical lab, outcome measures include maximum arterial blood level nicotine concentration (Cmax), the rate of nicotine rise after the initial puff (dCi/dt), time to maximum arterial blood level nicotine concentration (Tmax), area under the curve (AUC) for nicotine, liquid consumed (determined by ENDS gravimetric weight change, pre-post), and puffing topography records.

DIAGNOSIS AND DEPENDENCE MEASURES. In the first visit to the clinic, participants will complete several surveys to assess their nicotine dependence. These includes the PROMIS Nicotine Dependence Scale that assesses the severity of nicotine dependence on cigarettes and the corresponding 4-item E-Cigarette Dependence Scale (EDS) that assesses dependence on ENDS. In addition, they will complete the Fagerstrom Test of Nicotine Dependence questionnaires.

DRUG EFFECTS. Product liking, craving, and the drug effects will be assessed on a subjective scale (0-100, not at all-extremely) immediately following product use. Craving (QSU) and withdrawal symptoms (Minnesota Nicotine Withdrawal Scale) will be assessed at baseline and following ENDS use. The Drug Effect Questionnaire (DEQ) will measure acute effects consisting of seven items: drug strength, high, feeling stimulated, good effects, bad effects, wanting more drugs, and drug liking.

## 8.2 Safety and Other Assessments

Safety will be assessed at each stage of the study by the RA and RN using the health assessments checklist. There will be ongoing review by the PI and study physician with positive findings of a change in symptom or single severe symptom triggering clinical referral to treating physician or ED. The RA will ensure that the enrolled study subjects are eligible and sign informed consent. The study RN will be present with the study subject during all procedures, and the study subject's vital signs (HR, BP) will be measured by continuous monitoring. Any adverse events will be documented as described below.

## 8.3 Adverse Events and Serious Adverse Events

### 8.3.1 Definition of Adverse Events (AE)

Adverse event means any untoward medical occurrence associated with the use of an intervention in humans, whether or not considered intervention-related (21 CFR 312.32 (a)).

### 8.3.2 Definition of Serious Adverse Events (SAE)

An adverse event (AE) or suspected adverse reaction is considered "serious" if, in the view of either the investigator or sponsor, it results in any of the following outcomes: death, a life-threatening adverse event, inpatient hospitalization or prolongation of existing hospitalization, a persistent or significant incapacity or substantial disruption of the ability to conduct normal life functions, or a congenital anomaly/birth defect. Important medical events that may not result in death, be life-threatening, or require hospitalization may be considered serious when, based upon appropriate medical judgment, they may jeopardize the participant and may require medical or surgical intervention to prevent one of the outcomes listed in this definition. Examples of such medical events include allergic bronchospasm requiring intensive treatment in an emergency room or at home, blood dyscrasias or convulsions that do not result in inpatient hospitalization, or the development of drug dependency or drug abuse.

## 8.3.3 Classification of an Adverse Event

### 8.3.3.1 Severity of Event

For adverse events (AEs) not included in the protocol defined grading system, the following guidelines will be used to describe severity.

- Mild — Events require minimal or no treatment and do not interfere with the participant's daily activities.
- Moderate — Events result in a low level of inconvenience or concern with the therapeutic measures. Moderate events may cause some interference with functioning.
- Severe — Events interrupt a participant's usual daily activity and may require systemic drug therapy or other treatment. Severe events are usually potentially life-threatening or incapacitating. Of note, the term "severe" does not necessarily equate to "serious".

### 8.3.3.2 Relationship to Study Intervention

All adverse events (AEs) must have their relationship to study intervention assessed by the clinician who examines and evaluates the participant based on temporal relationship and his/her clinical judgment. The degree of certainty about causality will be graded using the categories below. In a clinical trial, the study product must always be suspect.

- **Related** — The AE is known to occur with the study intervention, there is a reasonable possibility that the study intervention caused the AE, or there is a temporal relationship between the study intervention and event. Reasonable possibility means that there is evidence to suggest a causal relationship between the study intervention and the AE.
- **Not Related** — There is not a reasonable possibility that the administration of the study intervention caused the event, there is no temporal relationship between the study intervention and event onset, or an alternate etiology has been established.

OR

- **Definitely Related** — There is clear evidence to suggest a causal relationship, and other possible contributing factors can be ruled out. The clinical event, including an abnormal laboratory test result, occurs in a plausible time relationship to study intervention administration and cannot be explained by concurrent disease or other drugs or chemicals. The response to withdrawal of the study intervention (dechallenge) should be clinically plausible. The event must be pharmacologically or phenomenologically definitive, with use of a satisfactory rechallenge procedure if necessary.
- **Probably Related** — There is evidence to suggest a causal relationship, and the influence of other factors is unlikely. The clinical event, including an abnormal laboratory test result, occurs within a reasonable time after administration of the study intervention, is unlikely to be attributed to concurrent disease or other drugs or chemicals, and follows a clinically reasonable response on withdrawal (dechallenge). Rechallenge information is not required to fulfill this definition.
- **Potentially Related** — There is some evidence to suggest a causal relationship (e.g., the event occurred within a reasonable time after administration of the trial medication). However, other factors may have contributed to the event (e.g., the participant's clinical condition, other concomitant events). Although an AE may rate only as "possibly related" soon after discovery, it can be flagged as requiring more information and later be upgraded to "probably related" or "definitely related", as appropriate.
- **Unlikely to be related** — A clinical event, including an abnormal laboratory test result, whose temporal relationship to study intervention administration makes a causal relationship improbable (e.g., the event did not occur within a reasonable time after administration of the study intervention) and in which other drugs or chemicals or underlying disease provides plausible explanations (e.g., the participant's clinical condition, other concomitant treatments).
- **Not Related** — The AE is completely independent of study intervention administration, and/or evidence exists that the event is definitely related to another etiology. There must be an alternative, definitive etiology documented by the clinician.

### 8.3.3.3 Expectedness

The PI and study team will be responsible for determining whether an adverse event (AE) is expected or unexpected. An AE will be considered unexpected if the nature, severity, or frequency of the event is not consistent with the risk information previously described for the study intervention.

### 8.3.4 Time Period and Frequency for Event Assessment and Follow-Up

The occurrence of an adverse event (AE) or serious adverse event (SAE) may come to the attention of study personnel during study visits and interviews of a study participant presenting for medical care, or upon review by a study monitor.

All AEs including local and systemic reactions not meeting the criteria for SAEs will be captured on the appropriate case report form (CRF). Information to be collected includes event description, time of onset, clinician's assessment of severity, relationship to study product (assessed only by those with the training and authority to make a diagnosis), and time of resolution/stabilization of the event. All AEs occurring while on study must be documented appropriately regardless of relationship. All AEs will be followed to adequate resolution.

Any medical condition that is present at the time that the participant is screened will be considered as baseline and not reported as an AE. However, if the study participant's condition deteriorates at any time during the study, it will be recorded as an AE.

Changes in the severity of an AE will be documented to allow an assessment of the duration of the event at each level of severity to be performed. AEs characterized as intermittent require documentation of onset and duration of each episode.

The PI, study coordinator, or research assistant will record all reportable events with start dates occurring any time after informed consent is obtained until 7 (for non-serious AEs) or 30 days (for SAEs) after the last day of study participation. At each study visit, the investigator will inquire about the occurrence of AE/SAEs since the last visit. Events will be followed for outcome information until resolution or stabilization.

### 8.3.5 Adverse Event Reporting

**Plan for reporting UPIRSOs (including Adverse Events) to the IRB**

The principal investigator will report the following types of events to the IRB: Any incident, experience or outcome that meets ALL 3 of the following criteria:

1. Is unexpected (in terms of nature, specificity, severity, or frequency) given (a) the research procedures described in the protocol-related documents, such as the IRB-approved protocol and informed consent document and (b) the characteristics of the subject population being studied; AND

2. Is related or possibly related to participation in the research (possibly related means there is a reasonable possibility that the incident, experience, or outcome may have been caused by the procedures involved in the research); AND

3. Suggests that the research places subjects or others at greater risk of harm (including physical, psychological, economic, legal, or social harm) than was previously known or recognized.

Unanticipated Problems Involving Risks to Subjects or Others (UPIRSOs) may be medical or non-medical in nature, and include — but are not limited to — serious, unexpected, and related adverse events and unanticipated adverse device effects. These UPIRSOs/SAEs will be reported to the IRB in accordance with Yale IRB Policy 710, using the appropriate forms found on the website. All related events involving risk but not meeting the prompt reporting requirements described in IRB Policy 710 should be reported to the IRB in summary form at the time of continuing review. If appropriate, such summary may be a simple brief statement that events have occurred at the expected frequency and level of severity as previously documented. In lieu of a summary of external events, a current DSMB report can be submitted for research studies that are subject to oversight by a DSMB (or other monitoring entity that is monitoring the study on behalf of an industry sponsor).

### 8.3.6 Serious Adverse Event Reporting

The study clinician will immediately report to the sponsor any serious adverse event, whether or not considered study intervention related, including those listed in the protocol or investigator brochure and must include an assessment of whether there is a reasonable possibility that the study intervention caused the event. Study endpoints that are serious adverse events (e.g., all-cause mortality) must be reported in accordance with the protocol unless there is evidence suggesting a causal relationship between the study intervention and the event (e.g., death from anaphylaxis). In that case, the investigator must immediately report the event to the sponsor.

All serious adverse events (SAEs) will be followed until satisfactory resolution or until the site investigator deems the event to be chronic or the participant is stable. Other supporting documentation of the event may be requested by the Data Coordinating Center (DCC)/study sponsor and should be provided as soon as possible.

The study sponsor will be responsible for notifying the Food and Drug Administration (FDA) of any unexpected fatal or life-threatening suspected adverse reaction as soon as possible, but in no case later than 7 calendar days after the sponsor's initial receipt of the information. In addition, the sponsor must notify FDA and all participating investigators in an Investigational New Drug (IND) safety report of potential serious risks, from clinical trials or any other source, as soon as possible, but in no case later than 15 calendar days after the sponsor determines that the information qualifies for reporting.

### 8.3.7 Reporting Events to Participants

Not applicable.

### 8.3.8 Events of Special Interest

Not applicable.

### 8.3.9 Reporting of Pregnancy (if applicable)

Pregnancy status will be directly communicated verbally to all study subjects who receive a pregnancy test prior to study procedures.

## 8.4 Unanticipated Problems

### 8.4.2 Unanticipated Problem Reporting

The investigator will report unanticipated problems (UPs) to the reviewing Institutional Review Board (IRB) and to the Data Coordinating Center (DCC)/lead principal investigator (PI). The UP report will include the following information:

- Protocol identifying information: protocol title and number, PI's name, and the IRB project number;
- A detailed description of the event, incident, experience, or outcome;
- An explanation of the basis for determining that the event, incident, experience, or outcome represents an UP;
- A description of any changes to the protocol or other corrective actions that have been taken or are proposed in response to the UP.

To satisfy the requirement for prompt reporting, UPs will be reported using the following timeline:

UPs will be promptly reported to the IRB within 5 business days and to the DCC/study sponsor in accordance with policy and regulatory requirements.

### 8.4.3 Reporting Unanticipated Problems to Participants

Participants will be individually informed about unanticipated problems.

# 9 - Statistical Considerations

## 9.1 Statistical Hypotheses

We hypothesize that computed nicotine flux will predict the rate of nicotine rise and maximum nicotine concentration observed in arterial blood, and that protonated nicotine will result in higher delivery.

## 9.2 Sample Size Considerations

Assuming an alpha level of 0.05 and an ICC of 0.4 (which conservatively assumes only modest correlation between Cmax measures within the same participant), we estimated that we would need a total sample size of 15 subjects to detect with 90% power a medium effect size of 0.4. With an anticipated higher ICC and a higher relationship between flux and Cmax, as suggested by our prior work, this design will be well-powered to investigate the relationship of Cmax and flux concentrations for each form separately, and over 88% power to detect an effect size 0.4 for the between and within group comparisons [i.e., form*concentration]. We will continue recruitment until we achieve the targeted sample size.

## 9.3 Populations for Analyses

E-Cigarette Users

## 9.4 Statistical Analyses

To account for the repeated Cmax (adjusted for baseline at time zero) assessments design and to evaluate the extent of within-subject correlation of Cmax outcomes, we will first compute using linear mixed effect models, the intra-class correlation coefficients for the Cmax measures, first separately for each of the forms (protonated and free base) and then for all Cmax measurements from the same participant. This step will help us understand the magnitude within-subject correlation of Cmax measures and to compare the within-subject correlation for the two main forms. Next, for each of the forms, we will investigate how Cmax relates to changing nicotine flux, by using linear mixed effect models (accounting for the within-subject design) and flux conditions as a predictor; we will investigate flux conditions both categorically and continuously to detect whether the change in Cmax follows a specific linear trend or whether some flux concentrations relate differently to Cmax. We will use model fit criteria to select the correlation structure and representation of flux concentrations that best describes the data. This investigation will be complemented by splines and locally weighted scatterplot smoothing (LOWESS) graphs to visualize plots of Cmax changes over the flux values. These analyses will be performed separately for the protonated and freebase conditions to assess similarities and differences in how the form of nicotine is influencing the Cmax-flux concentration relationship. Finally, we will integrate the form effect by using linear mixed models with both concentration and form as predictors as well as an interaction term between form*concentration to investigate whether different form-concentration combinations influence Cmax differently. The same sequence of analysis will be completed for the other outcomes of interest: dCi/dt, Tmax and AUC, to evaluate how the different fluxes and forms relate to rate of rise of nicotine, the time to Cmax and to cumulative nicotine exposure over time. We will investigate the relationship of participants' characteristics, such as sex, age, history of smoking, and baseline dependency scores with our pharmacokinetics outcomes. We will also look at whether some of these characteristics, namely sex, change the relationship between nicotine flux and the outcomes. In additional analysis, we will evaluate whether nicotine fluxes are related to changes in puffing intensity. We will also repeat the main analyses adjusting for puffing intensity.

### 9.4.1 General Approach

As above.

### 9.4.2 Analysis of the Primary Efficacy Endpoint(s)

As above.

### 9.4.3 Analysis of the Secondary Endpoint(s)

N/A

### 9.4.4 Safety Analyses

Safety events will be analyzed descriptively in aggregate.

### 9.4.5 Baseline Descriptive Analyses (if applicable)

N/A

### 9.4.6 Planned Interim Analyses (if applicable)

N/A

### 9.4.7 Sub-Group Analyses

N/A

### 9.4.8 Tabulation of Individual Participant Data

N/A

### 9.4.9 Exploratory Analyses

N/A

# 10 - Supporting Documentation and Operational Considerations

## 10.1 Regulatory, Ethical, and Study Oversight Considerations

### 10.1.1 Informed Consent Process

Subjects who are determined eligible for this protocol will be contacted and this protocol will be described to them. If they are interested in participating, subjects will be scheduled for study dates and will be given the consent form on their first visit. The consent form and protocol will be explained to them in person and they will be given the opportunity to read the consent form and sign it.

### 10.1.1.1 Consent/Assent and Other Informational Documents Provided to Participants

Consent forms describing in detail the study intervention, study procedures, and risks are given to the participant and written documentation of informed consent is required prior to starting intervention/administering study intervention. The following consent materials are submitted with this protocol: Compound Authorization & Consent For Participation in a Research Project.

### 10.1.1.2 Consent Procedures and Documentation

Consent forms will be Institutional Review Board (IRB)-approved and the participant/legally authorized representative (LAR) will be asked to read and review the document. The principal investigator and co-investigator will explain the research study to the participant and answer any questions that may arise. This conversation will take place in a private room. Assent will not be conducted. A verbal explanation will be provided in terms suited to the participant's comprehension of the purposes, procedures, and potential risks of the study and of their rights as research participants. Participants/families/LAR will have the opportunity to carefully review the written consent form and ask questions prior to signing. The participants/family/LAR should have the opportunity to discuss the study with their family or surrogates, or think about it prior to agreeing to participate. The participant will sign the informed consent document prior to any procedures being done specifically for the study. Participants/families/LAR must be informed that participation is voluntary and that they may withdraw from the study at any time, without prejudice. A copy of the informed consent document will be given to the participants/families/LAR for their records. eConsent will be completed using YCCI’s REDCap. The consent process will be completed by a research assistant or the PI in-person during the initial screening visit. Participants will be given as much time as they require to review the consent form and decide whether to sign consent.

### 10.1.2 Study Discontinuation and Closure

This study may be temporarily suspended or prematurely terminated if there is sufficient reasonable cause. Written notification, documenting the reason for study suspension or termination, will be provided by the suspending or terminating party to study participants, investigators, and funding agency. If the study is prematurely terminated or suspended, the Principal Investigator (PI) will promptly inform study participants, the Institutional Review Board (IRB), and sponsor and will provide the reason(s) for the termination or suspension. Study participants will be contacted, as applicable, and be informed of changes to study visit schedule.

Circumstances that may warrant termination or suspension include, but are not limited to:

- Determination of unexpected, significant, or unacceptable risk to participants
- Demonstration of efficacy that would warrant stopping
- Insufficient compliance to protocol requirements
- Data that are not sufficiently complete and/or evaluable
- Determination that the primary endpoint has been met
- Determination of futility

Study may resume once concerns about safety, protocol compliance, and data quality are addressed, and satisfy the sponsor, IRB and/or Food and Drug Administration (FDA).]

### 10.1.3 Data Confidentiality and Participant Policy

Participant confidentiality and privacy is strictly held in trust by the participating investigators, their staff, and the sponsor(s) and their interventions. This confidentiality is extended to cover testing of biological samples and genetic tests in addition to the clinical information relating to participants. Therefore, the study protocol, documentation, data, and all other information generated will be held in strict confidence. No information concerning the study or the data will be released to any unauthorized third party without prior written approval of the sponsor.

All research activities will be conducted in as private a setting as possible.

The study monitor, other authorized representatives of the sponsor, representatives of the Institutional Review Board (IRB), regulatory agencies or pharmaceutical company supplying study product may inspect all documents and records required to be maintained by the investigator, including but not limited to, medical records (office, clinic, or hospital) and pharmacy records for the participants in this study. The clinical study site will permit access to such records.

The study participant's contact information will be securely stored at each clinical site for internal use during the study. At the end of the study, all records will continue to be kept in a secure location for as long a period as dictated by the reviewing IRB, Institutional policies, or sponsor requirements.

Study participant research data, which is for purposes of statistical analysis and scientific reporting, will be transmitted to and stored at the Yale School of Medicine. This will not include the participant's contact or identifying information. Rather, individual participants and their research data will be identified by a unique study identification number. The study data entry and study management systems used by clinical sites and by Yale School of Medicine research staff will be secured and password protected. At the end of the study, all study databases will be de-identified and archived at the Yale School of Medicine.

Certificate of Confidentiality (if applicable)

To further protect the privacy of study participants, a Certificate of Confidentiality will be issued by the National Institutes of Health (NIH). This certificate protects identifiable research information from forced disclosure. It allows the investigator and others who have access to research records to refuse to disclose identifying information on research participation in any civil, criminal, administrative, legislative, or other proceeding, whether at the federal, state, or local level. By protecting researchers and institutions from being compelled to disclose information that would identify research participants, Certificates of Confidentiality help achieve the research objectives and promote participation in studies by helping assure confidentiality and privacy to participants.

### 10.1.4 Future Use of Stored Human Specimens and Data

Not Applicable.

### 10.1.5 Safety Oversight

Safety oversight will be under the direction of a Data and Safety Monitoring Board (DSMB) composed of individuals with the appropriate expertise, including nicotine and tobacco use. Members of the DSMB should be independent from the study conduct and free of conflict of interest, or measures should be in place to minimize perceived conflict of interest. The DSMB will meet at least yearly to assess safety and efficacy data on each arm of the study. The DMSB will operate under the rules of an approved charter that will be written and reviewed at the organizational meeting of the DSMB. At this time, each data element that the DSMB needs to assess will be clearly defined. The DSMB will provide its input to the study investigators.

**Data and Safety Monitoring Board (DSMB)**

The Yale TCORS has previously established a DSMB to provide the highest protection for study participants. Given the close collaboration and overlapping safety concerns between Aim 1 of the proposed study and those currently being conducted by TCORS, we will utilize the existing DSMB for the proposed study. The composition of the DSMB has been constructed following NIH guidelines and includes the following members who also served on the DSMB in TCORS-1:

| **Member** | **Affiliation** | **Role** |
| --- | --- | --- |
| Tony George, MD | Prof. Psychiatry, U. of Toronto | DSMB Chair |
| Hanga Galfalvy, PhD | Assoc. Prof. Psychiatry, Columbia U. | Statistician |
| Thomas Brandon, PhD | Prof. Psychology, U. South FL | Content Expert |

*Procedures*: The Core, in collaboration with the PIs, will prepare twice yearly DSMB reports, which include information on enrollment, participant retention rates and adverse events, and preliminary analyses as appropriate. The DSMB oversees an initial protocol review and then ongoing reviews. These reports are supplemented by rapid notification of all studies’ serious adverse events. The PIs will meet with the DSMB to review each study. The DSMB will meet initially in an open session, attended by the PIs. Then, a closed session is held in which the DSMB Chair conducts a review of all issues and puts these to vote. The purview of the DSMB includes, but is not limited to, assessments of data quality and timeliness, participant recruitment, subject retention, safety and efficacy data, and protocol compliance. The DSMB also considers advances occurring elsewhere and their impact on the potential risks and benefits of the study. Following the DSMB meeting, a report of the meeting, including recommendations will be prepared and submitted to the PIs, to the IRBs, and to the funding agency.

The principal investigator will be responsible for monitoring the data, assuring protocol compliance, and conducting the safety reviews at the specified frequency, which must be conducted at a minimum of every 6 months (including when reapproval of the protocol is sought). REDCap will be used for secure data storage and analysis. During the review process, the principal investigator (monitor) will evaluate whether the study should continue unchanged, require modification/amendment, or close to enrollment. Either the principal investigator, the IRB, or study sponsor have the authority to stop or suspend the study or require modifications. The PI will review safety data, after every test day, during weekly research team meetings, and will suspend or modify the study (with IRB approval) if indicated. The IRB will be duly informed if there are any reasons to warrant “holding” the study. A review of the study will be submitted to the IRB annually. Although we have assessed the proposed study as one of greater than minimal risk, the potential exists for anticipated and/or unanticipated adverse events, serious or otherwise, to occur since it is not possible to predict with certainty the absolute risk in any given individual or in advance of first-hand experience with the proposed study methods.

### 10.1.6 Key Roles and Study Governance

| Principal Investigator |
| --- |
| Stephen Baldassarri, MD, MHS |
| Yale School of Medicine |
| 300 Cedar St, TAC 455-S |
| 914-589-9086 |
| stephen.baldassarri@yale.edu |

### 10.1.7 Clinical Monitoring

Clinical site monitoring is conducted to ensure that the rights and well-being of trial participants are protected, that the reported trial data are accurate, complete, and verifiable, and that the conduct of the trial is in compliance with the currently approved protocol/amendment(s), with International Conference on Harmonisation Good Clinical Practice (ICH GCP), and with applicable regulatory requirement(s).

- Monitoring will be conducted by the Yale TCORS Data Safety Monitoring Board at annual intervals.

### 10.1.8 Quality Assurance and Quality Control

Each clinical site will perform internal quality management of study conduct, data and biological specimen collection, documentation and completion. An individualized quality management plan will be developed to describe a site's quality management.

Quality control (QC) procedures will be implemented beginning with the data entry system and data QC checks that will be run on the database will be generated. Any missing data or data anomalies will be communicated to the site(s) for clarification/resolution.

Following written Standard Operating Procedures (SOPs), the monitors will verify that the clinical trial is conducted and data are generated and biological specimens are collected, documented (recorded), and reported in compliance with the protocol, International Conference on Harmonization Good Clinical Practice (ICH GCP), and applicable regulatory requirements (e.g., Good Laboratory Practices (GLP), Good Manufacturing Practices (GMP)).

The investigational site will provide direct access to all trial related sites, source data/documents, and reports for the purpose of monitoring and auditing by the sponsor, and inspection by local and regulatory authorities.

## 10.1.9 Data Handling and Record Keeping

### 10.1.9.1 Data Collection and Management Responsibilities

Data collection is the responsibility of the clinical trial staff at the site under the supervision of the site investigator. The investigator is responsible for ensuring the accuracy, completeness, legibility, and timeliness of the data reported.

All source documents should be completed in a neat, legible manner to ensure accurate interpretation of data.

Hardcopies of the study visit worksheets will be provided for use as source document worksheets for recording data for each participant enrolled in the study. Data recorded in the electronic case report form (eCRF) derived from source documents should be consistent with the data recorded on the source documents.

Clinical data (including adverse events (AEs), concomitant medications, and expected adverse reactions data) and clinical laboratory data will be entered into REDCap, a 21 CFR Part 11-compliant data capture system provided by the Yale School of Medicine. The data system includes password protection and internal quality checks, such as automatic range checks, to identify data that appear inconsistent, incomplete, or inaccurate. Clinical data will be entered directly from the source documents.

### 10.1.9.2 Study Records Retention

HHS funded study documents should be retained for a minimum of 3 years after the termination of the study. Study documents will be retained for a period of at least 4 years after the date on which the investigation is terminated or completed or the date that the records are no longer considered necessary as described in the draft guidance document, Use of Investigational Tobacco Products: Guidance for Industry and Investigators.” These documents should be retained for a longer period, however, if required by local regulations. No records will be destroyed without the written consent of the sponsor, if applicable. It is the responsibility of the sponsor to inform the investigator when these documents no longer need to be retained.

### 10.1.10 Protocol Deviations

A protocol deviation is any noncompliance with the clinical trial protocol, International Conference on Harmonisation Good Clinical Practice (ICH GCP), or Manual of Procedures (MOP) requirements. The noncompliance may be either on the part of the participant, the investigator, or the study site staff. As a result of deviations, corrective actions are to be developed by the site and implemented promptly.

These practices are consistent with ICH GCP:

- 4.5 Compliance with Protocol, sections 4.5.1, 4.5.2, and 4.5.3
- 5.1 Quality Assurance and Quality Control, section 5.1.1
- 5.20 Noncompliance, sections 5.20.1, and 5.20.2.

It is the responsibility of the site investigator to use continuous vigilance to identify and report deviations within 7 working days of identification of the protocol deviation, or within 14 working days of the scheduled protocol-required activity. All deviations must be addressed in study source documents, reported to NIDA Program Official and Yale School of Medicine. Protocol deviations must be sent to the reviewing Institutional Review Board (IRB) per their policies. The site investigator is responsible for knowing and adhering to the reviewing IRB requirements. Further details about the handling of protocol deviations will be included in the MOP.

### 10.1.11 Publication and Data Sharing Policy

This study will be conducted in accordance with the following publication and data sharing policies and regulations:

National Institutes of Health (NIH) Public Access Policy, which ensures that the public has access to the published results of NIH funded research. It requires scientists to submit final peer-reviewed journal manuscripts that arise from NIH funds to the digital archive PubMed Central upon acceptance for publication.

This study will comply with the NIH Data Sharing Policy and Policy on the Dissemination of NIH-Funded Clinical Trial Information and the Clinical Trials Registration and Results Information Submission rule. As such, this trial will be registered at ClinicalTrials.gov, and results information from this trial will be submitted to ClinicalTrials.gov. The ClinicalTrials.gov ID number for this study is NCT05706701. In addition, every attempt will be made to publish results in peer-reviewed journals. Data from this study may be requested from other researchers 5 years after the completion of the primary endpoint by contacting the Principal Investigator, Dr. Soha Talih. All data collected at YSM will be shared with AUB as part of the subcontract agreement between the two organizations.

### 10.1.12 Conflict of Interest Policy

The independence of this study from any actual or perceived influence, such as by the pharmaceutical industry, is critical. Therefore, any actual conflict of interest of persons who have a role in the design, conduct, analysis, publication, or any aspect of this trial will be disclosed and managed. Furthermore, persons who have a perceived conflict of interest will be required to have such conflicts managed in a way that is appropriate to their participation in the design and conduct of this trial. The study leadership in conjunction with the NIH has established policies and procedures for all study group members to disclose all conflicts of interest and will establish a mechanism for the management of all reported dualities of interest.

## 10.2 Additional Considerations

None

## 10.3 Protocol Amendment History

| Version | Date | Description of Change | Brief Rationale |
| --- | --- | --- | --- |
|  |  |  |  |
|  |  |  |  |
|  |  |  |  |
|  |  |  |  |
|  |  |  |  |
|  |  |  |  |
|  |  |  |  |
|  |  |  |  |
|  |  |  |  |
|  |  |  |  |
|  |  |  |  |
|  |  |  |  |
|  |  |  |  |
|  |  |  |  |
|  |  |  |  |
|  |  |  |  |
|  |  |  |  |
|  |  |  |  |
|  |  |  |  |
|  |  |  |  |
|  |  |  |  |
|  |  |  |  |
|  |  |  |  |
|  |  |  |  |

## 10.4 Abbreviations

| AE | Adverse Event |
| --- | --- |
| ANCOVA | Analysis of Covariance |
| CFR | Code of Federal Regulations |
| CLIA | Clinical Laboratory Improvement Amendments |
| CMP | Clinical Monitoring Plan |
| COC | Certificate of Confidentiality |
| CONSORT | Consolidated Standards of Reporting Trials |
| CRF | Case Report Form |
| DCC | Data Coordinating Center |
| DHHS | Department of Health and Human Services |
| DSMB | Data Safety Monitoring Board |
| DRE | Disease-Related Event |
| EC | Ethics Committee |
| eCRF | Electronic Case Report Forms |
| FDA | Food and Drug Administration |
| FDAAA | Food and Drug Administration Amendments Act of 2007 |
| FFR | Federal Financial Report |
| GCP | Good Clinical Practice |
| GLP | Good Laboratory Practices |
| GMP | Good Manufacturing Practices |
| GWAS | Genome-Wide Association Studies |
| HIPAA | Health Insurance Portability and Accountability Act |
| IB | Investigator's Brochure |
| ICH | International Conference on Harmonization |
| ICMJE | International Committee of Medical Journal Editors |
| IDE | Investigational Device Exemption |
| IND | Investigational New Drug Application |
| IRB | Institutional Review Board |
| ISM | Independent Safety Monitor |
| ISO | International Organization for Standardization |
| ITT | Intention-To-Treat |
| LSMEANS | Least-squares Means |
| MedDRA | Medical Dictionary for Regulatory Activities |
| MOP | Manual of Procedures |
| MSDS | Material Safety Data Sheet |
| NCT | National Clinical Trial |
| NIH | National Institutes of Health |
| NIH IC | NIH Institute or Center |
| OHRP | Office for Human Research Protections |
| PI | Principal Investigator |
| QA | Quality Assurance |
| QC | Quality Control |
| SAE | Serious Adverse Event |
| SAP | Statistical Analysis Plan |
| SMC | Safety Monitoring Committee |
| SOA | Schedule of Activities |
| SOC | System Organ Class |
| SOP | Standard Operating Procedure |
| UP | Unanticipated Problem |
| US | United States |
