# Supplementary material for: Nicotine flux as a powerful tool for regulating nicotine delivery from e-cigarettes: Protocol of two complimentary randomized crossover clinical trials
Source: PLoS One. 2023 Sep 21;18(9):e0291786. doi: 10.1371/journal.pone.0291786 (PMC10513228; doi:10.1371/journal.pone.0291786)
Supplement: S3 Appendix — (PDF) [file pone.0291786.s004.pdf]

<u>Consent to participate in a research study</u>

Nicotine flux, a potentially powerful tool for regulating nicotine delivery from electronic cigarettes: significance of nicotine flux to the rate of nicotine delivery and subjective effects

Principal investigator (PI): Dr. Soha Talih
Address: American University of Beirut
Beirut, Lebanon
Phone: (01) 350 000, ext: 3627
Mobile: 03-939064

Site where the study will be conducted: American University of Beirut

You are being asked to participate in a clinical research study conducted at the American University of Beirut. Please take time to read the following information carefully before you decide whether you want to take part in this study or not. Feel free to ask the investigator if you need more information or clarification about what is stated in this form and the study as a whole.

A. Project Description, Aims, and Recruitment Strategy

Nicotine flux, the rate of nicotine emissions per unit time, is a performance measure that can be used to evaluate and compare relative nicotine delivery from electronic nicotine delivery systems (ENDS) (also known as e-cigarettes) and potentially other nicotine delivery products. The main aim of this study is to assess the influence of nicotine flux and nicotine form on subjective effects in natural use environment. Users will be asked to puff on an ENDS device and subjective effects related to dependence will be assessed (product liking, nicotine craving). The ENDS device will be attached to a previously validated sampling device to collect a fraction of the aerosol generated during each puff to verify actual nicotine flux and form and measure exposure to pulmonary toxicants. This sampling device also records puffing behavior (puff volume, duration, interpuff interval) and does not interfere with the user experience of using the ENDS device. Five ENDS use sessions will be conducted at the AUB Aerosol Research Laboratory (ARL). Each session will be carried out during a different study visit and will differ by flux and form with five subjective measures (nicotine dependence, drug effects, product liking, and craving) measured during every visit. Each session consists of 2 bouts (10 puffs + 60 min as you please) with 2 fluxes (18, 35µg/s) x 2 forms (protonated, freebase) and a 0 nicotine condition.

Regulating ENDS products to minimize addiction is a public health priority. A promising approach is one that limits the speed at which ENDS devices deliver nicotine. This research is being conducted to collect much needed data to test the hypotheses that nicotine flux and form predict the speed and dose of delivery to the brain, as well as the magnitude of subjective effects relevant to addiction. This study will provide regulators a powerful empirically tested metric to limit the risk profile of ENDS products. The involvement of human subjects in this study is unavoidable, as the produced data of actual puffing behavior and subjective effects is directly needed by researchers and regulatory agencies worldwide and thus there is no alternative way to achieve the goal of this study.

American University of Beirut
Institutional Review Board
27 June 2022
APPROVED

American University of Beirut
Institutional Review Board
20 December 2022
APPROVED

15-09-2021

Flyers will be posted on Social Media. A survey form will appear for screening after participants scan the QR code or use the provided link. Participants can also call the number on the flyer or send an email to the email address on the flyer, we will schedule a phone conversation with them or we will share with them a survey access link and code by email, to screen for eligibility by asking them among other questions if they are dual ENDS and tobacco cigarette users, above 18 years old and if, in case they are women, are pregnant or breastfeeding. If they are eligible, they will be asked to come to AUB where we will explain to them the study aims, procedure, and risks and benefits, after which they will be asked to sign the informed consent. Before coming to the lab they will be asked to stop smoking for at least 12 hours before the session. Abstinence will be tested using a Carbon Monoxide breath analyzer in which one blows to verify that they haven't smoked prior to arrival. In case of sudden pregnancy, participants will be withdrawn from the study due to the risks of smoking in pregnancy.

Eligible participants will be asked to visit the lab five times. A member of the research team will give you a tablet to answer questionnaires. The data that will be collected include: basic demographics, nicotine dependence, use patterns of ENDS and tobacco cigarettes as well as sensations felt during/after the smoking session.

The questionnaires will take approximately 30 minutes. The total duration of each visit will take approximately 3 hours. Visits will be separated by 48 hours washout periods for each participant. The duration of the subject participation depends on how often each subject can attend and can last between 2 weeks and 6 months.

Approximate number of subjects to be recruited: 130

Duration of the study: 3 years.

The schematic representation below roughly illustrates the steps of each visit:

B. Risks and Benefits

You have the right to withdraw your consent or discontinue participation at any time for any reason. Your decision to withdraw will not involve any penalty or loss of benefits to which you are entitled.

American University of Beirut
Institutional Review Board
27 June 2022
APPROVED

American University of Beirut
Institutional Review Board
20 December 2022
APPROVED

Discontinuing participation in no way affects your relationship with AUB.
The investigator may end your participation at any time.

Financial compensation of 50 USD/session for 5 sessions will be given to you for your participation in this study.
You will not be asked to pay any costs for participating in the study.

Participating in this study poses a minimal risk since you will be smoking an ENDS device similar to the one you use on regular basis. This device and its liquid will be stored using a stringent condition protocol and used liquids will be discarded each day. Moreover, the puffing recording device does not interfere with your use of the ENDS device, and no additional behavior is required.

C. Confidentiality

If you agree to participate in this research study, the personal information will be kept confidential. Unless required by law, only the study PI and designee, the ethics committee and inspectors from governmental agencies will have direct access to the collected data.

To secure the confidentiality of your responses, your name and other identifying information will never be attached to your answers.
All participant files and information will be stored in a locked cabinet and all virtual data will be stored in a password protected computer that is kept secure. Data access is limited to the PI and researchers working directly on this project. If there is any breach in data safety, a detailed report will be sent to the IRB.

All data will be destroyed responsibly after the required retention period (usually three years). Your privacy will be maintained in all published and written data resulting from this study. Your name or other identifying information will not be used in our reports or published papers. You may abstain from answering any question that might cause you distress.

D. Contact Information

1) If you have any questions or concerns about the research you may contact Dr. Soha Talih, 01350000 ext 3627, email: st38@aub.edu.lb
2) If you have any questions, concerns or complaints about your rights as a participant in this research, you can contact the following office at AUB:

Biomedical Institutional Review Board
AUB
PO BOX: 11-0236 F15
Riad El Solh, Beirut, Lebanon 1107 2020
Tel: 00961 1 374374, ext: 5445
Fax: 00961 1 374374
Email: irb@aub.edu.lb Direct Line: 000961 1 738024

American University of Beirut
Institutional Review Board
27 June 2022
APPROVED

American University of Beirut
Institutional Review Board
20 December 2022
APPROVED

15-09-2021

E. Participant rights

Participation in this study is voluntary. You are free to leave the study at any time without penalty. Your decision not to participate in no way influences your relationship with AUB.

Investigator's Statement:

I have reviewed, in detail, the informed consent document for this research study with _________ _________________________ (name of patient, legal representative, or parent/guardian), the purpose of the study, and its risks and benefits. I have answered to all the patient's questions clearly. I will inform the participant in case of any changes to the research study.

___________________________ ___________________

Name of Investigator or designee Signature

____

Date & Time

Patient's Participation:

I have read and understood all aspects of the research study and all my questions have been answered. I voluntarily agree to be a part of this research study and I know that I can contact Dr. Soha Talih at (01) 350 000, ext: 3627 or 03-939064 or any of his/her designee involved in the study in case of any questions. If I feel that my questions have not been answered, I can contact the Institutional Review Board for human rights at (01) 350000 or (01) 374374, ext: 5445. I understand that I am free to withdraw this consent and discontinue participation in this project at any time, even after signing this form, and it will not affect my care or benefits. I know that I will receive a copy of this signed informed consent.

_______________________________ ______________________________

Name of Patient or Legal Representative or Parent/Guardian Signature

____

Date & Time

_______________________________ _______________________________

Witness's Name (if patient, representative or parent do not read) Witness's Signature

____

Date & Time

American University of Beirut
Institutional Review Board
27 June 2022
APPROVED

American University of Beirut
Institutional Review Board
20 December 2022
APPROVED

15-09-2021
