# Supplementary material for: Nicotine flux as a powerful tool for regulating nicotine delivery from e-cigarettes: Protocol of two complimentary randomized crossover clinical trials
Source: PLoS One. 2023 Sep 21;18(9):e0291786. doi: 10.1371/journal.pone.0291786 (PMC10513228; doi:10.1371/journal.pone.0291786)
Supplement: S4 Appendix — (PDF) [file pone.0291786.s005.pdf]

IRB Proposal

Nicotine flux, a potentially powerful tool for regulating nicotine delivery from electronic cigarettes: significance of nicotine flux to the rate of nicotine delivery and subjective effects

Principal investigator:

Soha Talih, PhD
Assistant Research Professor
Department of Mechanical Engineering
American University of Beirut
PO Box 11-0236, Riad El-Solh 1107 2020
Beirut, Lebanon
Telephone Number: +961-1-350000, ext. 3627
Email: st38@aub.edu.lb

Team members:

- Eliana Hanna
- Pia Maria Ghanime
- Ahmad El-Hellani
- Farid Talih
- Martine El-Bejjani
- Alan Shihadeh
- Najat Saliba
- Luna Geagea

1. Abstract

Electronic nicotine delivery systems (ENDS) heat and vaporize a nicotine-containing liquid to produce an aerosol that can deliver nicotine to the blood and the brain. ENDS use has increased rapidly in the last decade, especially among youth: over 20% of US high school students are current ENDS users, and there is evidence of nicotine dependence in this population. Federal legislation has been proposed that would restrict ENDS liquid nicotine concentration to make ENDS "significantly less addictive and appealing to youth." However, these and other efforts to curb addiction by limiting nicotine liquid concentration are unlikely to succeed because nicotine emissions from ENDS depend on multiple variables. To achieve the intended public health aims,

American University of Beirut
Institutional Review Board
27 June 2022
APPROVED

regulations targeting addiction must focus on nicotine delivery, not nicotine concentration. While nicotine delivery cannot be regulated directly, the rate at which an ENDS emits nicotine, the "nicotine flux", can be regulated and, importantly, predicted based on a few device design and operating variables. However, to date there is no empirical evidence demonstrating the relationship between flux and delivery, nor between flux and the subjective effects that support nicotine dependence. Closing this gap is essential for providing an effective framework for regulating ENDS. In Aim1 (at Yale University), we will examine the relationship between nicotine flux, nicotine form, and the rate and dose of nicotine delivery. In the clinical lab at Yale University, participants will puff on ENDS devices under conditions that differ by flux and form, while arterial blood is sampled in high time resolution. In Aim2 (at the American University of Beirut), we will assess the relationship between nicotine flux, form, and subjective effects. At AUB, participants will use ENDS devices with varying nicotine fluxes and forms. Dependency measures, such as urge to smoke, craving, and abstinence, will be assessed. The outcome will indicate the degree to which nicotine flux/form influence subjective effects related to dependency, puffing intensity, and exposure to toxicants. In summary, this project will provide the empirical evidence needed for public health agencies to use nicotine flux as an encompassing and convenient construct to regulate nicotine delivery from ENDS.

2. Aims

This study has two aims:
1. **Examine the influence of nicotine flux and nicotine form on the rate and dose of nicotine delivery to arterial blood.** At the Yale School of Medicine, we will measure arterial nicotine concentrations over discrete time-periods (baseline, 3xpost-puff, 60sec post-use) in a within-subject study with 15 participants who will undergo 2 ENDS use sessions that differ by nicotine form (protonated, freebase).
Because Aim1 will be conducted at Yale University, we will focus in this proposal on Aim2 which will be conducted at AUB.

2. Assess the influence of nicotine flux and nicotine form on subjective effects. At AUB, we will assess subjective effects (e.g. product liking, nicotine craving) and puffing topography for 130 participants who will undergo 5 ENDS use sessions consisting of 2 bouts (10 puffs + 60min *ad libitum*) with 2 fluxes (35, 70µg/s) x 2 forms (protonated, freebase) and a 0 nicotine condition. In addition, we will use a state-of-the-art device (NIDA 1R01DA025659) to sample *in situ* a fraction of the aerosol generated during each puff to verify actual nicotine flux and form, and measure exposure to pulmonary toxicants (carbonyls). *We hypothesize that increasing nicotine flux and protonated nicotine will result in greater reductions of nicotine craving, and lower puffing intensity and carbonyl exposure.*

3. Background

Historically, nicotine yield has served as the metric for characterizing (and mischaracterizing) the nicotine dose delivered by different combustible cigarette products.[1] Yield is defined as

American University of Beirut
Institutional Review Board
27 June 2022
APPROVED

the mass of nicotine emitted through the mouth end of a tobacco product per unit of consumption (e.g., mg/cig).[2] Because combustible cigarettes are relatively standardized products with similar use session durations (i.e., burn time), the nicotine yield of combustible cigarettes is also an indicator of nicotine flux. ENDS, on the other hand, vary widely in liquid reservoir capacity and power, and can be used sporadically over several days, during which hundreds of puffs can be drawn; there is no clearly defined unit of consumption around which yield can be compared across products.[3] In this respect, ENDS are more like motor vehicles, which come in a wide range of shapes and sizes, and which can be driven indefinitely if fuel and other supplies are available. Motor vehicles are subject to US Environmental Protection Agency (EPA) emission standards and requirements. To regulate fuel consumption, the US EPA focuses on performance measures, like the rate of fuel consumption, expressed in miles-per-gallon (MPG), rather than the size of the fuel tank (gallons).[4] Analogously, the amount of nicotine held by an ENDS device or the total nicotine yield cannot be used as a stand-in for nicotine delivery, any more than the size of a motor vehicle fuel tank indicates how efficiently it uses fuel or how far it will travel in its lifetime. Nicotine flux, the rate of nicotine emissions per unit time, is a performance measure that can be used to evaluate and compare relative nicotine delivery from ENDS and potentially other nicotine delivery products.[5, 6] Fig 1 shows the extent to which ENDS vary in terms of nicotine flux,[7-11] and thus potentially abuse liability, from the equivalent of a nicotine patch to double that of a combustible cigarette.[12-27] *To the extent that flux is demonstrated empirically to predict the nicotine delivery to blood and brain and impact subjective measures related to dependency, it may be used as a measure of nicotine delivery and, thus, abuse liability across ENDS and other nicotine delivery products.* ***At the American University of Beirut, we will try to demonstrate that nicotine flux is directly associated with subjective measured related to nicotine dependency.***

4. Methods

The purpose of Aim2 (at AUB) is to test the influence of nicotine flux and form on subjective effects related to dependency.[28] Aim2 will test the subjective effects of two nicotine fluxes (18 and 35µg/s) coupled with two ratios of nicotine form (100% free-base or 100% protonated). Additionally, a 0mg/mL nicotine concentration condition, will be used for comparison making a total of 5 conditions (Fig 2). Hence, each participant will attend the lab for five different visits that differ by nicotine flux and/or form in random order. All sessions will be double-blind. Similar to Aim1, before each visit, participants will be instructed to abstain from nicotine/tobacco and/or ENDS use for ≥12 h. Following a one-hour observation period, participants will use Subox Mini C in a 10-puff directed (30sec inter-puff interval) bout.[29, 30] This observation period is necessary because has been shown to cut participants' nonadherence to the abstinence condition to <10% (Spindle et al., 2018). One hour after the first bout, participants will be instructed to puff on the device for 60-min *ad libitum*. Subjective measures (i.e., nicotine dependence, drug effects, product liking, and craving) will be administered 5 times/session: 5 min before and 5 min after onset of the directed bout and 5 min before,

American University of Beirut
Institutional Review Board
27 June 2022
APPROVED

halfway into and 5 min after onset of the *ad libitum* bout. We note that this study cannot be accomplished as part of Aim1 because the highly invasive methods used in that study may interfere with subjective effects and the cost at the required scale would be prohibitive. In Aim2, we will also use REALTIME to trap a fraction of the aerosol emitted by the ENDS during each puff generated by the user (Fig 3), which affords the opportunity to examine a secondary hypothesis that increasing flux will result in both reduced puffing intensity and reduced exposure to pulmonary toxicants.

Statistical analysis: For the primary analysis of flux-subjective effects relationship, we will first evaluate the intra-class correlation coefficients of subjective measures within each tested condition, then within forms (3 fluxes: 0, 35, and 70ug/sec and 5 assessments for each condition), to inform the degree of correlation of subjective measures within the same flux condition and within the same form. We will then use linear mixed effect model to assess how the subjective measures change across the 5 time points (i.e., the 5 measurements around the directed and *ad libitum* bouts) for each nicotine flux, and across fluxes, and between forms. The analyses will look at each level separately and gradually build the significant levels/components of the model. They will also include graphical representation of how subjective assessments change in time for each condition and across conditions (through splines and LOWESS plots of the subjective parameters in relation to time assessments for each flux condition, and in relation to the different concentrations for the various conditions tested for each form). Structure of correlations and modeling of timing of questionnaire for each condition will be assessed using model fit criteria. Similarly, predictors of interest, including sex, age, history of smoking, and baseline dependency scores will be investigated.

Sample size: With alpha<0.05 and an ICC of 0.4, we estimated requiring 65 subjects to detect for each form, and with 90% power, a small effect size of 0.2 in the changes of subjective assessments for each form. To integrate both the repeated assessments for each form and the comparison between the two forms and with the 0 nicotine level condition, following a two-level design (first level being the 5 repeated time point assessments within a condition; and second level being the cluster of 5 conditions form/flux combinations per subject), we estimate a total sample size of 130 subjects to detect with 85% power an effect size of 0.5; hence aim 2 will recruit 130 participants. We will continue recruitment until we achieve the targeted sample size.

Materials

Clinical setting of Aim2. Users will be asked to puff on an ENDS device with the designated nicotine fluxes and forms, and subjective effects related to dependence will be measured. The ENDS device will be attached to a previously validated (NIDA 1R01DA025659) real-time in-situ sampling (REALTIME) device that unobtrusively samples the aerosol exiting the ENDS mouthpiece during each puff for subsequent chemical assays; the device also records puffing topography (puff volume, duration, interpuff interval). Aerosol samples collected using REALTIME will be analyzed for nicotine yield and form, and carbonyl emissions (please see the outcome measures and the analytical lab work sections for details). In this manner mouth-level

American University of Beirut
Institutional Review Board
27 June 2022
APPROVED

exposure to nicotine and pulmonary toxicants for each individual participant will be assessed. Liquid consumption for each participant use session will be determined by pre- and post-weighing the ENDS device.

OUTCOME MEASURES. The outcome measures include subjective effects related to dependency, puff topography records, amount of liquid consumed, nicotine (yield, flux, and form) and carbonyl compound exposure.

DIAGNOSIS AND DEPENDENCE MEASURES. In the first visit to the clinic, participants will complete several surveys to assess their nicotine dependence. These includes the PROMIS Nicotine Dependence Scale[31] that assesses the severity of nicotine dependence on cigarettes and the corresponding 4-item E-Cigarette Dependence Scale (EDS) that assesses dependence on ENDS.[32-34] In addition, they will complete the Fagerstrom Test of Nicotine Dependence questionnaires.[35]

DRUG EFFECTS. Product liking, craving, and the drug effects will be assessed on a subjective scale (0-100, not at all-extremely) immediately following product use. Product liking will be assessed by the Electronic Cigarette Specific Effects questionnaire,[36] general labeled magnitude scale (gLMS),[37] and labeled hedonic scale (LHS).[38] Craving (QSU)[39, 40] and withdrawal symptoms (Minnesota Nicotine Withdrawal Scale)[41] will be assessed at baseline and following ENDS use. The Drug Effect Questionnaire (DEQ) will measure acute effects consisting of seven items: drug strength, high, feeling stimulated, good effects, bad effects, wanting more drugs, and drug liking.[42, 43]

INCLUSION/EXCLUSION CRITERIA. All the participants must be healthy and above 18 years of age. In addition, participants must be willing to provide informed consent, attend the lab, and abstain from tobacco/nicotine as required. History of chronic disease or uncontrolled psychiatric condition is exclusionary, as is history of or active cardiovascular disease, low/high blood pressure, seizures, regular use of a prescription medication (except vitamins/birth control), and past month use of cocaine, opioids, benzodiazepines, or methamphetamines. Individuals who report using marijuana >15/30 days will be excluded. Women will be excluded if they are breast-feeding or pregnant at screening. Participants intending to quit tobacco/nicotine use in the next 30 days will be excluded and referred to cessation treatment. Prior to each session, participants will be instructed to abstain from nicotine/tobacco and/or ENDS use for ≥12h. Additionally, all participants will undergo a one-hour observation period prior to each study session during which no nicotine/tobacco product will be permitted.[30] We will also verify pre-session tobacco abstinence using expired air CO (BreathCO monitor, Vitalagraph). Given that most ENDS users are dual users of ENDS and combustible cigarettes,[44] we will recruit dual users only to avoid a potential confounding variable. A dual ENDS and tobacco user reports daily use of ENDS (≥ 3 mg/ml nicotine) or cigarettes (any frequency) AND someday use (≥ 3 days/week) of ENDS (≥ 3 mg/ml nicotine) or cigarettes (any frequency) for the past 3 months or longer.

HISTORY/DEMOGRAPHICS. Demographic data including age, gender, socio-economic status, marital status, educational, and occupational levels will be assessed using a screening survey. The collected information will be used to ascertain eligibility. The PROMIS Global Health measure (10

American University of Beirut
Institutional Review Board
27 June 2022
APPROVED

items) will provide an assessment of a participant's assessment of his/her health (physical, emotional, and social).[45]

Analytical lab work. We will conduct the analytical lab work at the AUB Aerosol Research Laboratory (ARL) and the Analytical and Atmospheric Laboratory (AAL). The AUB team has developed a multidisciplinary framework for aerosol generation, sampling, and analysis. To mitigate participant risk prior to onset of the study, we will verify the model's predictions by generating and measuring nicotine emissions using ALVIN and the actual ENDS devices and liquids that will be used in the clinical lab. Four technologies developed at the ARL are crucial for the completion of this project:

ALVIN. The Aerosol Lab Vaping Instrument (ALVIN) is a puffing robot that powers the ENDS via a dedicated feedback-controlled power circuit that precisely regulates voltage, while continuously monitoring and logging the electrical current, and therefore the power during each puff.[46] Also, ALVIN can reproduce previously recorded individual human puffing behavior in detail resolved to milliseconds. ALVIN has been used in several studies.[7, 22, 47, 48]

ETOP. Puff topography will be measured with "eTop", an instrument developed and manufactured by our team at AUB specifically for ENDS puffing topography. In brief, the instrument senses flow-induced pressure drop across an orifice that is incorporated into the mouthpiece. [49] The pressure drop is sensed by a pressure transducer whose output voltage is amplified, digitized, and sampled. The orifice dimensions and pressure-sensing transducer were designed to provide sensitivity sufficient to ensure valid measurements at puff velocities as low as 3mL/s. eTop utilizes a convenient automated calibration protocol that ensures accuracy and precision for each individual use instance. Currently, eTop technology is in use at 17 university research labs in the USA, including the YSM, and the ARL provides remote user support on demand.

REALTIME. Developed by our group, REALTIME (R01DA025659) consists of a computer-controlled flow meter, pump, sampling filter, and sampling bag.[50] The flow meter monitors the flow rate continuously in the mouthpiece. When a puff-induced non-zero flow is detected, the fast-response, miniature pump diverts a portion of the aerosol through a small diameter sampling probe installed in the mouthpiece. The pump is controlled (using the same closed-loop, self-learning algorithm used in [46]) to ensure that the aerosol is always sampled proportionally. During sampling, the pump draws the aerosol through a quartz filter that traps the particulate phase and a DNPH-coated cartridge that traps carbonyl compounds; the remaining gas phase is exhausted into an inert sampling Tedlar bag. By using the combination of filter, cartridge, and bag, the aerosol particulate and gas phases can be analyzed for nicotine and carbonyl compounds.[50]

The AAL is equipped with Gas Chromatography-Flame Ionization Detector (GC-FID) and High-performance Liquid Chromatography-Mass Spectrometry (HPLC-MS) to conduct all nicotine and carbonyl analysis. All ENDS liquids needed for this project will be prepared from standard chemicals (i.e., PG, VG, and nicotine) by experienced staff in the AAL. The optimized analytical

American University of Beirut
Institutional Review Board
27 June 2022
APPROVED

methods in AAL were shown to be useful in the analysis of nicotine and carbonyl emissions from ENDS in several projects.[7, 48]

Nicotine and carbonyls. The AAL has optimized and validated a simple liquid-liquid extraction analytical method for quantifying the total and individual forms of nicotine.[47] Nicotine in the aerosol is measured by immersing the filter pads collected in 6 mL water and shaking for 30 minutes. Then, 6mL toluene is added to extract free-base nicotine. The last step is repeated twice to ensure complete extraction. A solution of NaOH (200µL) is then added to the mixture to convert protonated into free-base nicotine. Nicotine is thereafter extracted using toluene. Total nicotine will be quantified by summing free-base and protonated nicotine. Nicotine analysis will be done on GC-FID. Carbonyl compounds will be determined using the method described in [7, 51]. In brief, derivatized carbonyl species are extracted from the 2,4-dinitrophenylhydrazine (DNPH) cartridges in 90/10 (vol/vol) ethanol/acetonitrile and quantified by HPLC equipped with an ultraviolet detector (HPLC-DAD). We will assess 14 carbonyl species: formaldehyde, acetaldehyde, acetone, acrolein, proprionaldehyde, crotonaldehyde, methacrolein, butyraldehyde, benzaldehyde, valeraldehyde, tolualdehyde, hexaldehyde, glyoxal, and methylglyoxal.

ENDS device, liquid, preparation and conditioning. We will use the Subox Mini C ENDS device because this device has proven to efficiently deliver nicotine to blood in our previous work.[36] Liquids will be prepared from a stock of analytical grade 30/70 PG/VG liquid. This ratio is prevalent in ENDS devices, including JUUL.[11] We predict with our mathematical model that for a Subox Min C operating at 15W with a 30/70 PG/VG liquid, nicotine concentrations of 1, 12, 17, and 25mg/mL will provide fluxes of 3, 35, 50, and 70µg/sec, respectively. Protonated nicotine liquids will be prepared by adding benzoic acid to the stock liquid of a given concentration.[11] The predicted nicotine fluxes for the Subox Mini C will be verified at AUB using ALVIN. A stringent device conditioning protocol will be used to ensure reproducibility in all studies, as we have reported in our publications.[52] All liquids will be stored in the dark at 5°C in sealed containers.

Quality assurance. QA/QC procedures will be followed during liquid preparation and analysis: validating methods, accuracy and precision, blanks, spikes, recovery, and limit of detection. Personnel training, safety and chemicals disposal, and chain of custody follow GLP and published protocols; the AUB Safety Office ensures compliance.

5. Budget

We summarize the budget in the list below:

A. Senior/key personnel: $198,780
B. Other personnel: $164,121
C. Equipment: NA
D. Travel: $15,000
E. Other direct costs

American University of Beirut
Institutional Review Board
27 June 2022
APPROVED

1. Materials and supplies: $67,131
2. Miscellaneous: $4,600
3. Supply shipping costs: $1,500
4. Publication costs: $4,000
5. Consultant services: $6,000
6. Sub-award costs: $673,786
7. Participant recruitment: $2,000
8. Participant payments: Each participant will be paid $250 in total for completing the study ($50 per visit).). Funds were requested to pay 146 subjects for participation ($36,500).
9. Participant transportation: Funds are requested to cover some transportation costs ($3,650).

Total costs per year:

Project year	Direct Costs in $	Total costs in $
1	299,906	461,578
2	299,784	461,390
3	299,754	461,344
Total	899,444	1,384,313

6. Timeline

Using our years of experience conducting similar studies, we have made a detailed realistic 3-year timeline for the completion of the project considering: inter-dependencies, equipment maintenance, and outages, and availability of resources and personnel to this project (Table 1). In Q 1-2, the AUB team will build the instruments required for the studies of Aim1&2. The recruitment of ENDS participants for the clinical study of Aim1 (at Yale University) will start in Q2. In the analytical lab, ENDS liquids with different nicotine concentrations and forms will be prepared in Q1&2, and the predicted nicotine flux from these liquids will be confirmed using ALVIN. Then, the clinical study at YSM will start and continue till the end of quarter 11. Recruitment for the clinical study of Aim2 at AUB will occur in Q2-11. Data collection and REALTIME sampling from Aim2 will take place during the whole project life similar to Aim1. Meetings between the study team members will be scheduled bi-monthly, and data dissemination will start towards the end of Q12.

American University of Beirut
Institutional Review Board
27 June 2022
APPROVED

	Table 1. Project Timeline	Year 1				Year 2				Year 3			
	Analytical lab work / Clinical lab work / Communication and data dissemination	1	2	3	4	5	6	7	8	9	10	11	12
	Analytical lab - Technology development												
Aim1	Clinical lab - Hire & train personnel												
	Analytical lab - liquid preparations												
	Analytical lab - aerosol sampling and nicotine measurements												
	Clinical lab - Recruitment (n=15)												
	Clinical lab - Follow-up												
	Clinical lab - Collect data												
	Clinical lab - Send topography data to AUB												
	Analytical lab - Topography data analysis												
Aim2	Clinical lab - Hire & train personnel												
	Clinical lab - Recruitment (n=130)												
	Clinical lab - Collect data												
	Analytical lab - RINS and topography data analysis												
	Bi-Monthly conference calls (AUB and Yale)												
	Preliminary presentations and manuscript presentations												
	Data analysis and manuscript-write up												

7. Ethical considerations

Informed consent: Since the research is of minimal risks: after screening participants through the QR code, on the phone or through the survey access link provided by flyer/email for eligibility, informed consent will be taken during the first visit in the clinical lab by the PI or the study coordinator after explaining the risks and benefits of the study. Participation will be entirely voluntary, and participants will have the right to withdraw their consent and continue participation at any time without penalty. Participants will be compensated with a $50 per visit (5 visits total). Finally, refusal to participate or withdraw from the study will involve no penalty or loss of benefits to which the participant is otherwise entitled, and neither will affect the relationship with AUB.

Confidentiality and privacy: To maintain privacy and confidentiality, identifying data will not be used or presented in the written data analysis resulting from the study.

8. Co-Investigators

Below is a list of the Co-investigators involved in this study, a detailed description is provided in the section that follows.

**Institution**	**Co-investigators**
**AUB**	Ahmad El-Hellani, PhD
	Farid Talih, MD
	Martine El-Bejjani, PhD
	Alan Shihadeh, ScD
	Najat Saliba, PhD
**Yale University**	Stephen Baldassarri, MD

More information on the study investigators:

American University of Beirut
Institutional Review Board
27 June 2022
APPROVED

This project brings together the efforts of three junior investigators that have at least five years of experience in ENDS tobacco product research, one senior researcher that is highly experienced in clinical trials related to addiction, a quantitative methods expert, and one senior tobacco regulatory science investigator. *Dr. Soha Talih (PI)* is a mechanical engineer who developed a physics-based mathematical model that predicts ENDS nicotine emissions. She has co-authored > 25 peer-reviewed articles on aspects of ENDS design, physics, emissions, and user behavior. Dr. Talih has extensive experience in project coordination and time management, mathematical modeling, data analysis and dissemination of results that she built in 5+ years of experience in managing the P50/U54DA036105 efforts at AUB. *Dr. Ahmad El-Hellani (Co-I)* is an organic/analytical chemist that has developed analytical methods needed for quantitative analysis of toxicant emissions from ENDS. He has co-authored > 20 peer-reviewed articles that elucidated some of the basic mechanisms that take place during ENDS activation. Both Drs. Talih and El-Hellani have worked in the transdisciplinary team of the Center for the Study of Tobacco Products (CSTP) for more than five years (P50/U54DA036105), and have written reports for the WHO Study Group on Tobacco Product Regulation (TobReg) and have presented their work in several international conferences. *Dr. Stephen Baldassarri (Co-I)* is a physician-scientist who works on the biomedical and behavioral aspects of ENDS use at the Yale School of Medicine (YSM). He is currently funded by NIDA to study the factors that influence ENDS nicotine delivery to the arterial blood and brain. He is co-Investigator of the NIDA/FDA funded Yale Center for the Study of Tobacco Product Use and Addiction (YCSTP) EVALI project supplement, which seeks to more fully understand the EVALI epidemic through active surveillance, toxicology, and biologic analysis of patient samples. *Dr. Farid Talih (Co-I)* is a psychiatrist and director of the addiction program at the AUB Medical Center (AUBMC). Dr. Talih has experience in multidisciplinary research and clinical trials on addiction and mental health including the collection of relevant blood biomarkers and the use of objective measurement tools (e.g., actigraphy). He has conducted several studies related to smoking products such as waterpipe and cannabis in which biomarkers were collected with 3 and 6 months follow up periods, and is currently involved in launching a study with Dr. El-Hellani on IQOS use patterns. *Dr. Martine Elbejjani (Co-I)* is an assistant professor of epidemiology at the Clinical Research Institute and director of the Research Education Unit at the Faculty of Medicine and AUBMC. Her research expertise are in neuro-epidemiology and in integrating advanced statistical methods to test neurobiological hypothesis of brain, cognitive, and mental health at the community level. She has conducted several investigations incorporating longitudinal and repeated data analyses on the relation of tobacco smoking with health outcomes, including cerebral blood flow, global and candidate brain region volumes, and other physiological processes, such as muscle and bone outcomes. In addition, she has extensive expertise in both primary data collection and analysis of repeated and multi-level data. *Dr. Alan Shihadeh* (Co-I) is a professor of mechanical engineering and directs the AUB arm of the CSTP TCORS. He is an aerosol scientist who over the past 20 years pioneered technologies and methods to study the physics, chemistry, and behavioral aspects of emerging tobacco products, including ENDS. He has advised WHO, FDA, and other public agencies on tobacco control issues.

American University of Beirut
Institutional Review Board
27 June 2022
APPROVED

Dr. Najat Saliba (Co-I) is an analytical chemist who specializes in the chemistry of airborne particles, including tobacco smoke. She has adapted analytical methods for examining toxicants in waterpipe tobacco smoke and has published more than 70 refereed articles, of which 20 address tobacco products. She has advised the WHO on tobacco control.

9. References

1. Maron, D.J. and S.P. Fortmann, *Nicotine yield and measures of cigarette smoke exposure in a large population: are lower-yield cigarettes safer?* Am J Public Health, 1987. **77**(5): p. 546-9.
2. Jarvis, M.J., et al., *Nicotine Yield From Machine-Smoked Cigarettes and Nicotine Intakes in Smokers: Evidence From a Representative Population Survey.* JNCI: Journal of the National Cancer Institute, 2001. **93**(2): p. 134-138.
3. Schroeder, M.J. and A.C. Hoffman, *Electronic cigarettes and nicotine clinical pharmacology.* Tobacco Control, 2014. **23**(suppl 2): p. ii30-ii35.
4. EPA. *Automotive Trends Report*. 2018; Available from: https://www.epa.gov/automotive-trends/highlights-automotive-trends-report#:~:text=The%20new%20vehicle%20emission%20rate,achieved%20in%20model%20year%202018.
5. Eissenberg, T. and A. Shihadeh, *Nicotine flux: a potentially important tool for regulating electronic cigarettes.* Nicotine Tob Res, 2015. **17**(2): p. 165-7.
6. Shihadeh, A. and T. Eissenberg, *Electronic cigarette effectiveness and abuse liability: predicting and regulating nicotine flux.* Nicotine Tob Res, 2015. **17**(2): p. 158-62.
7. El-Hellani, A., et al., *Nicotine and Carbonyl Emissions From Popular Electronic Cigarette Products: Correlation to Liquid Composition and Design Characteristics.* Nicotine Tob Res, 2018. **20**(2): p. 215-223.
8. Havel, C.M., et al., *An Electronic Cigarette Vaping Machine for the Characterization of Aerosol Delivery and Composition.* Nicotine Tob Res, 2017. **19**(10): p. 1224-1231.
9. Farsalinos, K.E., et al., *Protocol proposal for, and evaluation of, consistency in nicotine delivery from the liquid to the aerosol of electronic cigarettes atomizers: regulatory implications.* Addiction, 2016. **111**(6): p. 1069-76.
10. Goniewicz, M.L., et al., *High exposure to nicotine among adolescents who use Juul and other vape pod systems ('pods').* Tob Control, 2019. **28**(6): p. 676-677.
11. Talih, S., et al., *Characteristics and toxicant emissions of JUUL electronic cigarettes.* Tobacco Control, 2019. **28**(6): p. 678-680.
12. Herzok B and K. P. *Nielsen: Tobacco 'All Channel' Data 1/27*. Wells Fargo Equity Research Reports. 2018 Accessed Feb 11, 2021]; Available from: https://1lbxcx1bcuig1rfxaq3rd6w9-wpengine.netdna-ssl.com/wp-content/uploads/2018/02/Nielsen-Tobacco-All-Channel-Report-Period-Ending-1.27.18.pdf.

American University of Beirut
Institutional Review Board
27 June 2022
APPROVED

13. Piadé, J.J., et al., *Toxicological assessment of kretek cigarettes: Part 2: kretek and American-blended cigarettes, smoke chemistry and in vitro toxicity.* Regul Toxicol Pharmacol, 2014. **70 Suppl 1**: p. S15-25.
14. Goel, R., et al., *A Survey of Nicotine Yields in Small Cigar Smoke: Influence of Cigar Design and Smoking Regimens.* Nicotine Tob Res, 2018. **20**(10): p. 1250-1257.
15. Agnew-Heard, K.A., et al., *Multivariate Statistical Analysis of Cigarette Design Feature Influence on ISO TNCO Yields.* Chem Res Toxicol, 2016. **29**(6): p. 1051-63.
16. Bodnar, J.A., et al., *Mainstream smoke chemistry analysis of samples from the 2009 US cigarette market.* Regul Toxicol Pharmacol, 2012. **64**(1): p. 35-42.
17. Schmeltz, l., et al., *On the Chemistry of Cigar Smoke: Comparisons between Experimental Little and Large Cigars.* Beiträge zur Tabakforschung International/Contributions to Tobacco Research, 1976. **8**(6): p. 367-377.
18. Pickworth, W.B., et al., *Cigarillo and Little Cigar Mainstream Smoke Constituents from Replicated Human Smoking.* Chem Res Toxicol, 2018. **31**(4): p. 251-258.
19. Rickert, W.S., et al., *Characterization of tobacco products: a comparative study of the tar, nicotine, and carbon monoxide yields of cigars, manufactured cigarettes, and cigarettes made from fine-cut tobacco.* Prev Med, 1985. **14**(2): p. 226-33.
20. Darrall, K.G. and J.A. Figgins, *Roll-your-own smoke yields: theoretical and practical aspects.* Tobacco Control, 1998. **7**(2): p. 168-175.
21. Marcilla, A., et al., *Comparison between the mainstream smoke of eleven RYO tobacco brands and the reference tobacco 3R4F.* Toxicol Rep, 2014. **1**: p. 122-136.
22. Salman, R., et al., *Free-Base and Total Nicotine, Reactive Oxygen Species, and Carbonyl Emissions From IQOS, a Heated Tobacco Product.* Nicotine Tob Res, 2019. **21**(9): p. 1285-1288.
23. Farsalinos, K.E., et al., *Nicotine Delivery to the Aerosol of a Heat-Not-Burn Tobacco Product: Comparison With a Tobacco Cigarette and E-Cigarettes.* Nicotine Tob Res, 2018. **20**(8): p. 1004-1009.
24. Bekki, K., et al., *Comparison of Chemicals in Mainstream Smoke in Heat-not-burn Tobacco and Combustion Cigarettes.* J uoeh, 2017. **39**(3): p. 201-207.
25. El Hourani, M., et al., *Comparison of CO, PAH, Nicotine, and Aldehyde Emissions in Waterpipe Tobacco Smoke Generated Using Electrical and Charcoal Heating Methods.* Chem Res Toxicol, 2019. **32**(6): p. 1235-1240.
26. Shihadeh, A. and R. Saleh, *Polycyclic aromatic hydrocarbons, carbon monoxide, "tar", and nicotine in the mainstream smoke aerosol of the narghile water pipe.* Food Chem Toxicol, 2005. **43**(5): p. 655-61.
27. Shiffman, S., et al., *Using Nicotine Gum to Assist Nondaily Smokers in Quitting: A Randomized Clinical Trial.* Nicotine Tob Res, 2020. **22**(3): p. 390-397.
28. Jensen, K.P., et al., *Differential effects of nicotine delivery rate on subjective drug effects, urges to smoke, heart rate and blood pressure in tobacco smokers.* Psychopharmacology, 2020. **237**(5): p. 1359-1369.

American University of Beirut
Institutional Review Board
27 June 2022
APPROVED

29. Lopez, A.A., et al., *Effects of Electronic Cigarette Liquid Nicotine Concentration on Plasma Nicotine and Puff Topography in Tobacco Cigarette Smokers: A Preliminary Report.* Nicotine Tob Res, 2016. **18**(5): p. 720-3.
30. Spindle, T.R., et al., *Effects of electronic cigarette liquid solvents propylene glycol and vegetable glycerin on user nicotine delivery, heart rate, subjective effects, and puff topography.* Drug and Alcohol Dependence, 2018. **188**: p. 193-199.
31. Edelen, M.O., *The PROMIS® Smoking Assessment Toolkit—Background and Introduction to Supplement.* Nicotine & Tobacco Research, 2014. **16**(Suppl_3): p. S170-S174.
32. Morean, M.E., S. Krishnan-Sarin, and S. S.O'Malley, *Assessing nicotine dependence in adolescent E-cigarette users: The 4-item Patient-Reported Outcomes Measurement Information System (PROMIS) Nicotine Dependence Item Bank for electronic cigarettes.* Drug & Alcohol Dependence, 2018.
33. Morean, M.E., et al., *Psychometric Evaluation of the E-cigarette Dependence Scale.* Nicotine & Tobacco Research, 2018. **21**(11): p. 1556-1564.
34. Morean, M.E., et al., *Psychometric evaluation of the Patient-Reported Outcomes Measurement Information System (PROMIS)Nicotine Dependence Item Bankfor use with electronic cigarettes.* Nicotine & Tobacco Research, 2019. **22**(11): p. 2123-2123.
35. NIDA. *NIDA CTN Common Data Elements. Instrument: Fagerstrom Test For Nicotine Dependence (FTND)*. Available from: https://cde.drugabuse.gov/instrument/d7c0b0f5-b865-e4de-e040-bb89ad43202b.
36. Hiler, M., et al., *Effects of electronic cigarette heating coil resistance and liquid nicotine concentration on user nicotine delivery, heart rate, subjective effects, puff topography, and liquid consumption.* Exp Clin Psychopharmacol, 2020. **28**: p. 527-539.
37. Rosbrook, K. and B.G. Green, *Sensory Effects of Menthol and Nicotine in an E-Cigarette.* Nicotine Tob Res, 2016. **18**(7): p. 1588-95.
38. Lim, J., A. Wood, and B.G. Green, *Derivation and evaluation of a labeled hedonic scale.* Chem Senses, 2009. **34**(9): p. 739-51.
39. Toll, B.A., N.A. Katulak, and S.A. McKee, *Investigating the factor structure of the Questionnaire on Smoking Urges-Brief (QSU-Brief).* Addict Behav, 2006. **31**(7): p. 1231-9.
40. Tiffany, S.T. and D.J. Drobes, *The development and initial validation of a questionnaire on smoking urges.* Br J Addict, 1991. **86**(11): p. 1467-76.
41. Hughes, J.R. and D. Hatsukami, *Signs and Symptoms of Tobacco Withdrawal.* Arch Gen Psychiatry., 1986. **43**(3): p. 289-294.
42. Sofuoglu, M., et al., *Rapid nicotine clearance is associated with greater reward and heart rate increases from intravenous nicotine.* Neuropsychopharmacology, 2012. **37**(6): p. 1509-16.
43. Morean, M.E., et al., *The drug effects questionnaire: psychometric support across three drug types.* Psychopharmacology, 2013. **227**(1): p. 177-192.
44. CDC, *QuickStats: Cigarette Smoking Status Among Current Adult E-cigarette Users, by Age Group — National Health Interview Survey, United States, 2015.* MMWR Morb Mortal Wkly Rep, 2016. **65**: p. 1177.

American University of Beirut
Institutional Review Board
27 June 2022
APPROVED

45. Hays, R.D., et al., *Development of physical and mental health summary scores from the patient-reported outcomes measurement information system (PROMIS) global items.* Quality of Life Research, 2009. **18**(7): p. 873-880.
46. Shihadeh, A. and S. Azar, *A closed-loop control "playback" smoking machine for generating mainstream smoke aerosols.* J Aerosol Med, 2006. **19**(2): p. 137-47.
47. El-Hellani, A., et al., *Free-Base and Protonated Nicotine in Electronic Cigarette Liquids and Aerosols.* Chem Res Toxicol, 2015. **28**(8): p. 1532-7.
48. Talih, S., et al., *Effects of user puff topography, device voltage, and liquid nicotine concentration on electronic cigarette nicotine yield: measurements and model predictions.* Nicotine Tob Res, 2015. **17**(2): p. 150-7.
49. Shihadeh, A., C. Antonios, and S. Azar, *A portable, low-resistance puff topography instrument for pulsating, high-flow smoking devices.* Behav Res Methods, 2005. **37**(1): p. 186-91.
50. Katurji, M., et al., *Direct measurement of toxicants inhaled by water pipe users in the natural environment using a real-time in situ sampling technique.* Inhalation toxicology, 2010. **22**(13): p. 1101-1109.
51. Al Rashidi, M., A. Shihadeh, and N.A. Saliba, *Volatile aldehydes in the mainstream smoke of the narghile waterpipe.* Food Chem Toxicol, 2008. **46**(11): p. 3546-9.
52. Talih, S., et al., *Hot Wires and Film Boiling: Another Look at Carbonyl Formation in Electronic Cigarettes.* Chem Res Toxicol, 2020. **33**(8): p. 2172-2180.

American University of Beirut
Institutional Review Board
27 June 2022
APPROVED
