# Supplementary figures and images for: Nicotine flux as a powerful tool for regulating nicotine delivery from e-cigarettes: Protocol of two complimentary randomized crossover clinical trials
Source: PLoS One. 2023 Sep 21;18(9):e0291786. doi: 10.1371/journal.pone.0291786 (PMC10513228; doi:10.1371/journal.pone.0291786)

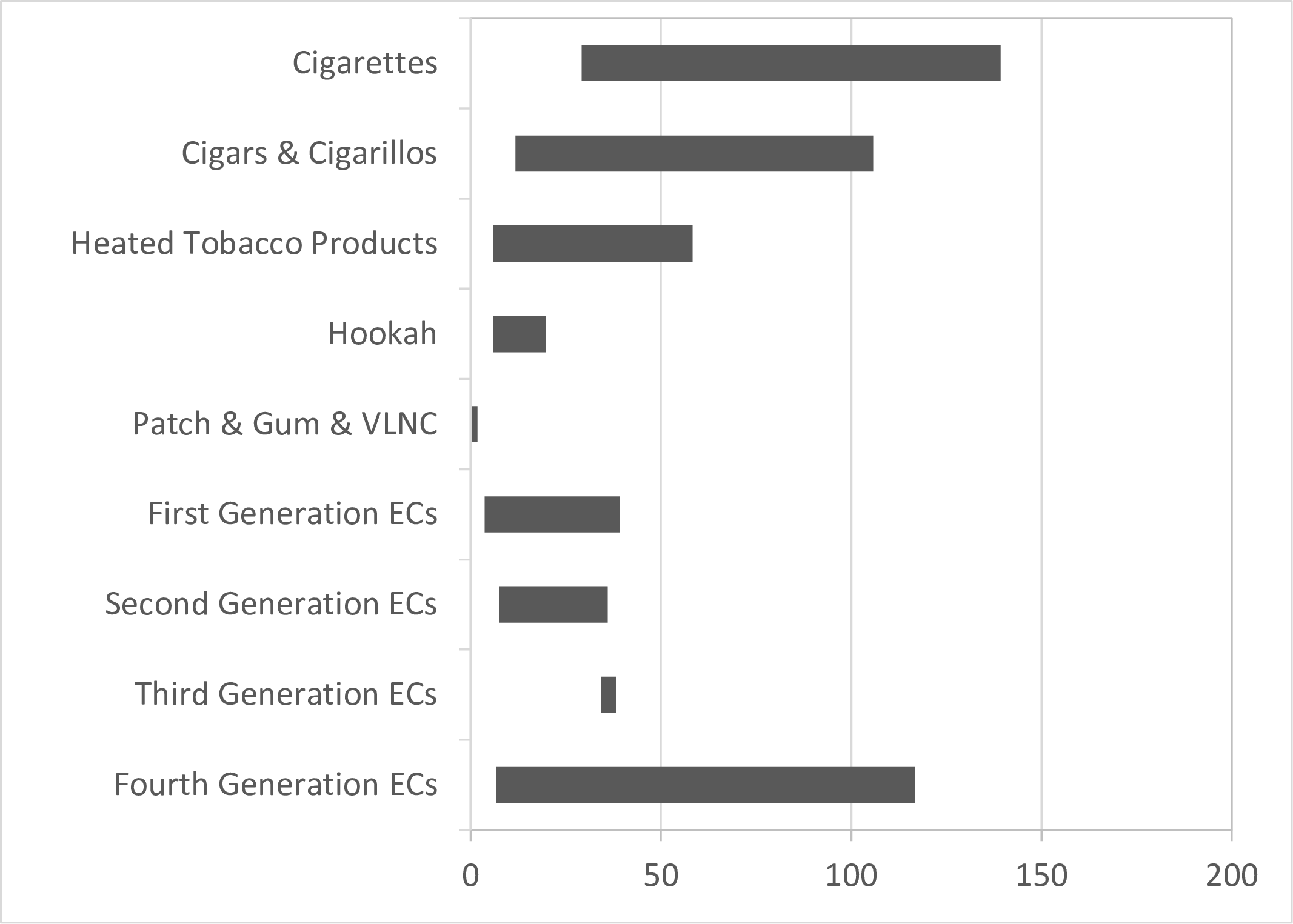

Supplement: S1 Fig — Generations of e-cigarettes were determined according to. (TIF) [file pone.0291786.s006.tif]
